# Striking the right balance of intermolecular coupling for high-efficiency singlet fission†
†Electronic supplementary information (ESI) available: Additional steady-state absorption spectra, sample structural characterization, and nanosecond and femtosecond transient absorption spectra and associated modelling details. See DOI: 10.1039/c8sc00293b


**DOI:** 10.1039/c8sc00293b

**Published:** 2018-06-01

**Authors:** Ryan D. Pensack, Andrew J. Tilley, Christopher Grieco, Geoffrey E. Purdum, Evgeny E. Ostroumov, Devin B. Granger, Daniel G. Oblinsky, Jacob C. Dean, Grayson S. Doucette, John B. Asbury, Yueh-Lin Loo, Dwight S. Seferos, John E. Anthony, Gregory D. Scholes

**Affiliations:** a Department of Chemistry , Princeton University , Princeton , New Jersey 08544 , USA . Email: gscholes@princeton.edu; b Department of Chemistry , University of Toronto , Toronto , Ontario M5S 3H6 , Canada; c Department of Chemistry , The Pennsylvania State University , University Park , Pennsylvania 16802 , USA; d Department of Chemical and Biological Engineering , Princeton University , Princeton , New Jersey 08544 , USA; e Department of Chemistry , University of Kentucky , Lexington , Kentucky 40506 , USA . Email: anthony@uky.edu; f Andlinger Center for Energy and the Environment , Princeton University , Princeton , New Jersey 08544 , USA; g Department of Chemical Engineering and Applied Chemistry , University of Toronto , Toronto , Ontario M5S 3E5 , Canada

## Abstract

Singlet fission is a process that splits collective excitations, or excitons, into two with unity efficiency. This exciton splitting process, unique to molecular photophysics, has the potential to considerably improve the efficiency of optoelectronic devices through more efficient light harvesting. While the first step of singlet fission has been characterized in great detail, subsequent steps critical to achieving overall highly-efficient singlet-to-triplet conversion are only just beginning to become well understood. One of the most elementary suggestions, which has yet to be tested, is that an appropriately balanced coupling is necessary to ensure overall highly efficient singlet fission; that is, the coupling needs to be strong enough so that the first step is fast and efficient, yet weak enough to ensure the independent behavior of the resultant triplets. In this work, we show how high overall singlet-to-triplet conversion efficiencies can be achieved in singlet fission by ensuring that the triplets comprising the triplet pair behave as independently as possible. We show that side chain sterics govern local packing in amorphous pentacene derivative nanoparticles, and that this in turn controls both the rate at which triplet pairs form and the rate at which they decay. We show how compact side chains and stronger couplings promote a triplet pair that effectively couples to the ground state, whereas bulkier side chains promote a triplet pair that appears more like two independent and long-lived triplet excitations. Our results show that the triplet pair is not emissive, that its decay is best viewed as internal conversion rather than triplet–triplet annihilation, and perhaps most critically that, in contrast to a number of recent suggestions, the triplets comprising the initially formed triplet pair cannot be considered independently. This work represents a significant step toward better understanding intermediates in singlet fission, and how molecular packing and couplings govern overall triplet yields.

## Abstract 2

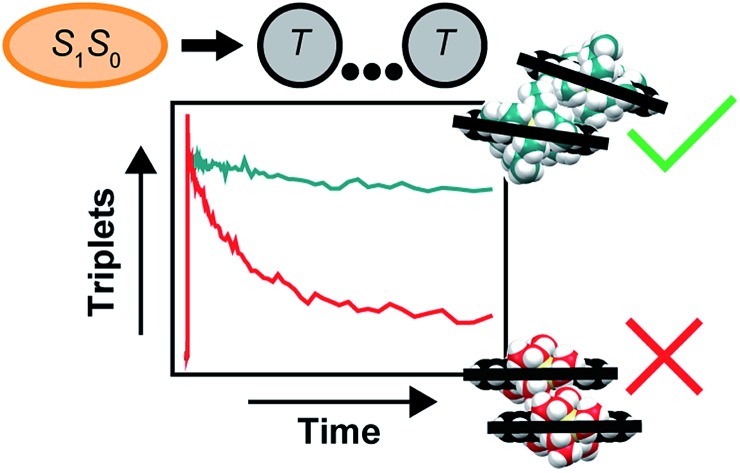
Bulky side chains promote a molecular packing and intermolecular coupling that effect high-efficiency singlet fission in amorphous solids of pentacene derivatives.

## Introduction

Singlet fission is an exciton splitting process in molecular materials that has attracted considerable attention because of its fundamental significance and technological potential. Incorporation of a singlet fission sensitizer into a photovoltaic cell, for example, more effectively harvests the solar spectrum and can boost the maximum device efficiency by greater than 30% (ref. 8). In order for the process to be useful, however, high overall singlet-to-triplet conversion efficiencies in singlet fission are imperative. The accepted model of singlet fission^9^,^10^ is shown below:

1 S_1_S_0_ ⇆ TT ⇆ T_1_ + T_1_

and involves the conversion of one light-absorbing, singlet exciton into an overall singlet, correlated triplet pair which subsequently decoheres to form two fully independent triplet excitations.^9^,^11^,^12^ Various authors^5^,^13^–^18^ have proposed a modified version of this kinetic scheme which additionally accounts for triplet pair separation:

2 S_1_S_0_ ⇆ [TT ⇆ T··· T] ⇆ T_1_ + T_1_

where the symbols TT and T···T are used to describe nascent and separated triplet pair intermediates, respectively. More specifically, we use the term nascent triplet pairs to describe triplet pairs populated immediately following the initial fission (internal conversion) step, and the term separated triplet pairs, *i.e.*, T···T, to describe the product of the dissociation, or spatial separation, of nascent triplet pairs.^1^,^5^,^12^–^20^

The majority of work to date has emphasized the first step of singlet fission,^20^–^64^ *i.e.*, triplet pair formation,^65^ while detailed studies of subsequent steps, such as triplet pair separation,^1^,^2^,^4^,^5^,^15^–^20^,^66^–^79^ have only recently elicited interest and are far fewer. Although understanding how to produce triplet pairs efficiently is important in moving toward the practical implementation of singlet fission, it is also important to ensure that the resultant triplets are long lived. In this context, it will be essential to satisfy one of the two following conditions: (i) both nascent and separated triplet pairs exhibit the desirable attributes of independent triplet excitations, *i.e.*, they are long-lived because relaxation to the ground state is spin forbidden (*i.e.*, T_1_ ↛ S_0_), or (ii) nascent triplet pairs efficiently convert into spatially separated triplet pairs, which can be assumed to exhibit many of the properties of independent triplet excitations.^5^,^12^,^66^ While overall quantitative triplet yields have been reported in selected crystalline material systems,^18^,^27^,^80^ losses are ubiquitous in disordered condensed-phase assemblies of chromophores, such as amorphous solids^18^,^32^ and solution-phase covalently-tethered molecules and oligomers.^3^,^4^,^68^,^70^,^81^–^87^ To date, there have been no reports of triplet pairs in covalently-tethered molecules or oligomers exhibiting a lifetime equivalent to that of isolated-chromophore triplet excitations.

Central to addressing these losses is a better understanding of the nature of the triplet pair. The emerging consensus from experiment is that the triplet pair comprises two individual triplet excitations;^1^–^7^ in this view, understanding the interactions (both spin and electronic) between triplets is considered central to understanding losses. More recently, this view has been developed further to acknowledge a unique, although unresolved, aspect of the triplet pair which is that it has “dual singlet-triplet optical character”, as has been reported in concentrated solutions,^88^ in the solid state,^5^ and in covalently-tethered molecular pairs.^6^ In contrast to the view that the triplet pair comprises two individual triplet excitations, *ab initio* calculations have indicated that the triplet pair is well described as a single entity with substantial doubly-excited electronic character that can effectively couple to the ground state.^66^,^68^,^89^ While differences between experiment and theory remain, the studies above could be unified by considering a more fundamental view where losses can be explained by considering how molecular packing influences the extent to which the constituent triplets act independently, either through ground-state intermolecular packing geometries^9^,^66^ or through those introduced *via* excited-state processes such as excimer relaxation.^61^,^68^,^90^

**Fig. 1:**
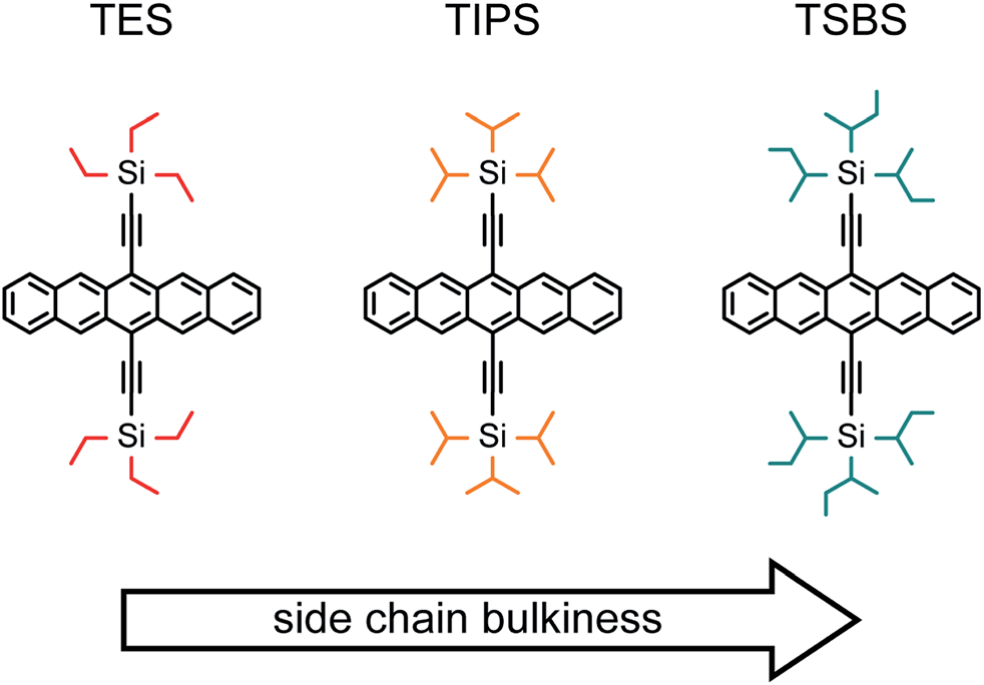
Chemical structures of the pentacene derivatives studied in this work arranged in order of increasing side chain bulkiness.

In this work, we show how overall highly-efficient singlet fission can be achieved in amorphous pentacene derivative nanoparticles through judicious tailoring of side chain sterics. We show that efficient singlet fission can be achieved with a coupling strong enough to ensure rapid and efficient triplet pair formation yet weak enough to ensure the independent nature of the triplets comprising the triplet pair. The three pentacene derivatives chosen for this study—6,13-bis(triethylsilylethynyl)pentacene (TES-Pn), 6,13-bis(tri*iso*propylsilylethynyl)pentacene (TIPS-Pn), and 6,13-bis(tri*sec*butylsilylethynyl)pentacene (TSBS-Pn)—are shown in Fig. 1, arranged in order of increasingly bulky side chains. Pentacene derivatives serve as exemplar chromophores because singlet fission is exoergic and each individual step of the process can be resolved. As a model of singlet fission in a disordered environment, we chose to study amorphous nanoparticles of these pentacene derivatives. Amorphous solids (*i.e.*, nanoparticles and films) lack the conformational flexibility in other disordered media, such as solution-phase covalently-tethered molecular pairs, yet retain the propensity to adopt multiple packing configurations. Although various strategies have been implemented to vary molecular packing in amorphous systems, such as by embedding molecules in an inert host matrix to increase the intermolecular spacing between chromophores,^91^ we (and others^61^) posited that side chains—which are known to play a profound role in the packing^92^–^95^—could be used as an incisive handle to influence local molecular packing, couplings relevant to singlet fission, and ultimately singlet fission dynamics.

## Results and discussion

### Singlet fission in amorphous pentacene derivative nanoparticles

**Fig. 2:**
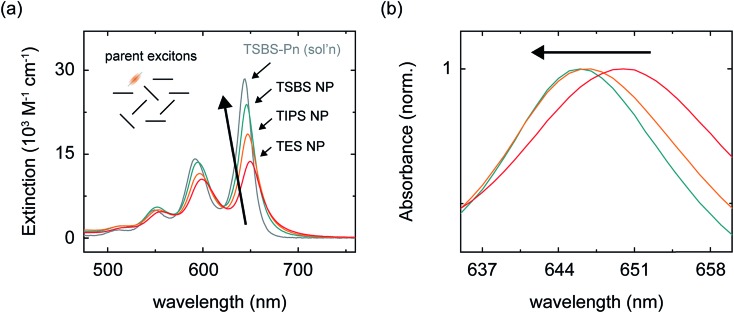
(a) Extinction spectra of the aqueous amorphous pentacene derivative nanoparticle suspensions and of a dilute solution of TSBS-pentacene in toluene. The inset shows a schematic representation of parent singlet excitons in the amorphous pentacene derivative nanoparticles, which we take to be completely absent of long-range order but to have the possibility of local structural order between nearest neighbors (see example main text and ref. 97). (b) Normalized absorption spectra of the aqueous amorphous pentacene derivative nanoparticle suspensions highlighting the peak position of the origin band of the lowest-energy singlet transition.

We prepared nanoparticles of these pentacene derivatives by rapidly injecting a concentrated solution of the compound dissolved in a “good” solvent (tetrahydrofuran) into a vigorously stirring solution of a “bad” solvent (water).^96^ This flash precipitation results in spherical nanoparticles with a diameter ranging from *ca.* 80–90 nm (Section S2†). Nanoparticles of these pentacene derivatives are amorphous, as evidenced by comparing their absorption spectra to that of amorphous and crystalline material (Section S3†). Here, we consider an amorphous material to be completely absent of long-range structural order but with the possibility of local, short-range order between nearest neighbors (for more details see footnote 97). The absorption spectra of the nanoparticles are similar to that of dilute solutions of the compounds in toluene (Fig. 2a and Section S3†) indicating that the chromophores in the nanoparticles are weakly coupled and that parent singlet excitons, *i.e.*, those accessed directly through vertical excitation, are primarily localized.

We hypothesized that increasingly bulky side chains would inhibit the close approach of two molecules. Fig. 2 shows that, consistent with this hypothesis, the absorption spectra of the amorphous nanoparticles look more and more like that of the isolated chromophore as side chains become increasingly bulky. This is because excitonic coupling and mass density—which can be evaluated *via* trends in the relative amplitude and peak positions of the vibronic bands comprising the lowest-energy singlet transition (for a full discussion see Section S4†)—decrease with increasingly bulky side chains. Specifically, Fig. 2a shows that as side chain bulkiness increases, the peak extinction associated with the origin vibronic band and the relative amplitude of the 0–0 and 0–1 vibronic bands both trend toward that of the isolated chromophore. In contrast, as side chains become more compact, we find that the peak extinction is reduced and the ratio of the amplitudes of the 0–0 and 0–1 vibronic bands is significantly smaller than that of the isolated chromophore, the latter observation indicating that a large fraction of the chromophores comprising the nanoparticles tend to adopt an H-type aggregate packing arrangement.^98^,^99^ Fig. 2b shows that as side chain bulkiness increases the vibronic origin band of the lowest-energy singlet transition blueshifts and trends towards that of the isolated chromophore as well. This subtle blueshift can be assigned to a smaller average effective mass density in the amorphous nanoparticles comprising pentacene derivatives with bulkier side chains.^100^ These results support our hypothesis that increasingly bulky side chains decrease excitonic coupling and prevent the close approach of two molecules in the amorphous nanoparticles.

**Fig. 3:**
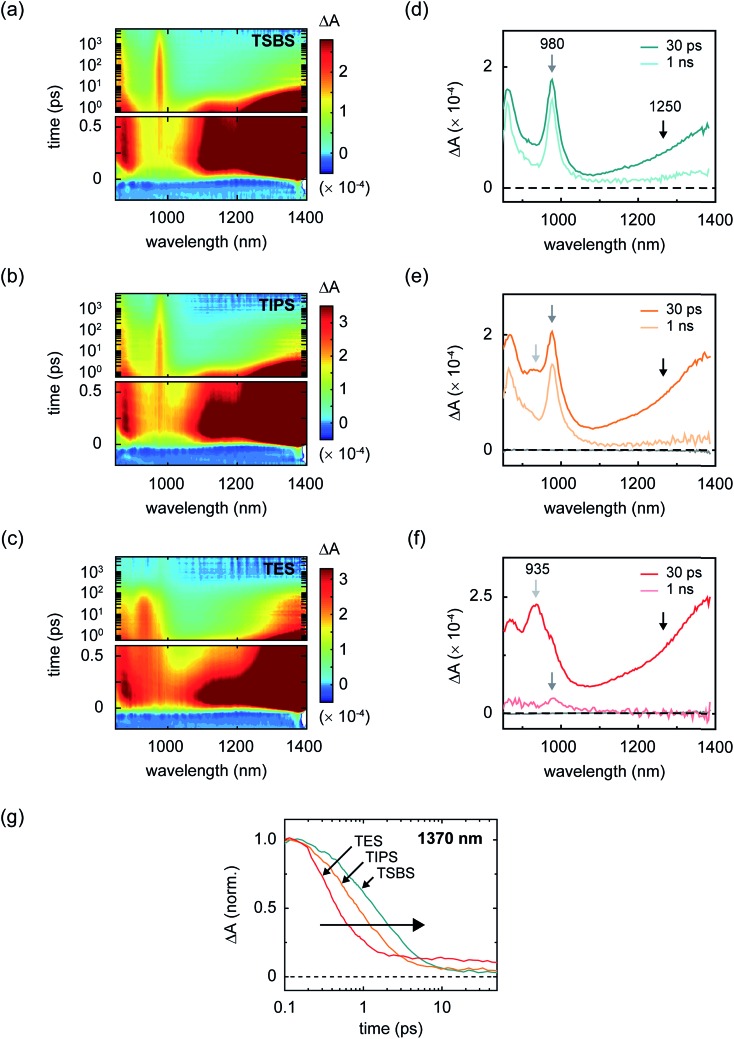
Transient near-infrared absorption of the amorphous pentacene derivative nanoparticles. (a–c) Surface plots of the transient near-infrared absorption of amorphous nanoparticles of TSBS-, TIPS-, and TES-pentacene. The parent singlet features appear off scale in order to highlight additional spectral features appearing at intermediate- and long-time delays. The transient measurements were performed with an incident pump fluence of *ca.* 190 μJ cm^–2^. The scale bar is indicated. (d–f) Selected transient absorption spectra at intermediate and long delay times for TSBS-, TIPS-, and TES-pentacene. Light grey, grey, and black arrows at 935, 980, and *ca.* 1250 nm highlight spectral features discussed in the main text. (g) Semilog plot of the transient absorption kinetics of the signal appearing in the vicinity of parent singlet exciton for the TSBS-, TIPS-, and TES-pentacene nanoparticles. The data were normalized to the signal amplitude at the time origin of the measurement, and shifted in time by *ca.* 100 fs to facilitate presentation.

Although increasingly bulky side chains tend to decrease excitonic coupling and mass density, all of the materials are found to undergo singlet fission (Fig. 3a–c). Parent singlet excitons in the amorphous pentacene derivative nanoparticles, monitored through a spectral band peaking in the near-infrared at 1400 nm (Section S5†), decay rapidly on a picosecond and sub-picosecond timescale in all samples. Concomitant with the decay of the parent singlet excitons, prominent triplet photoinduced absorption bands are observed (Fig. 3d–f and Section S6†), evidencing singlet fission as the primary excited-state decay pathway. We find that the decay is the fastest in the TES-Pn nanoparticles (Fig. 3g), indicating that the first step of singlet fission becomes faster as side chains become more compact and the molecules become closer. A faster initial step of singlet fission means that the largely H-type packing conformation adopted in the TES-Pn nanoparticles results in a larger matrix element coupling the parent singlet exciton and nascent triplet pair.^34^ A large matrix element coupling the parent singlet exciton and nascent triplet pair is often considered desirable for highly efficient singlet fission, and it is expected that maximizing this matrix element correspondingly maximizes triplet pair yields. We note that although several factors could potentially complicate a direct interpretation of the results in terms of the intrinsic timescale of triplet pair formation, including the concentration of singlet fission sites in the amorphous nanoparticles and singlet diffusion to them,^20^,^32^,^61^ qualitative arguments can be made such that our interpretation regarding coupling strengths relevant to the initial step of singlet fission still holds (Section S7†).

To estimate the yield of nascent triplet pairs in the amorphous pentacene derivative nanoparticles, we adopted the same approach that we used previously.^18^ Specifically, we estimate the nascent triplet pair yield according to a simple kinetic analysis, where we: (i) take the fluorescence decay of isolated chromophores (Section S8†) to account for all excited-state decay pathways competitive with singlet fission in the amorphous pentacene derivative nanoparticles,^101^ and (ii) take the rate of triplet pair formation as equivalent to the rate of decay of parent singlet excitons, which was determined by mathematically modeling the decay of the near-infrared singlet photoinduced absorption band (Section S9†). The results of the analysis indicate that, because of the picosecond and sub-picosecond triplet pair formation timescales in nanoparticles of these pentacene derivatives, maximizing the matrix element for triplet pair formation has little effect on the initial triplet pair yield; that is, we estimate essentially unity triplet pair yields in all cases (Table 1). Thus, although increasing side chain bulkiness decreases the rate of triplet pair formation, this has a negligible influence on the initial triplet pair yield.

**Table 1:** Triplet pair yield in amorphous pentacene derivative nanoparticles *via* a simple kinetic analysis^*a*^ ^,^^*b*^ ^,^^*c*^

Compound	*τ*_TPF_ (ps)	*Φ*_TT_ (%)
TES-Pn	0.42 ± 0.03	99.97
TIPS-Pn	1.23 ± 0.06	99.90
TSBS-Pn	2.8 ± 0.5	99.77

^*a*^The triplet pair formation (TPF) time constant was obtained by averaging over at least three independent measurements (and sample preparations). See *e.g.* Section S9. The limits represent an analysis of a single standard deviation of the time constants obtained from the fits.

^*b*^This estimate takes into account all unimolecular decay processes *via* the isolated chromophore fluorescence lifetime measured in toluene. All compounds exhibit an isolated chromophore fluorescence lifetime of *ca.* 12 ns (Section S8).

^*c*^More significant digits than an error analysis would allow are provided for the estimated triplet pair quantum yields to better facilitate comparison of results between samples.

In order for singlet fission to be practical, the resultant triplet pair excitation must be long lived so that it can be harvested efficiently. In this context, a reasonable benchmark to achieve for the triplet pair lifetime is that of isolated-chromophore triplet excitations. In the case of the present compounds, isolated-chromophore triplet excitations exhibit a lifetime of *ca.* 10 μs independent of side chain substituent (Section S10†) and, in the context of singlet fission sensitization, such a lifetime can be considered reasonably long lived.^102^ In the amorphous pentacene derivative nanoparticles, we observe a triplet pair lifetime that clearly falls short of this benchmark value. Namely, we find that although the initial yield of triplet pairs is essentially quantitative in all samples, losses at longer times indicate that a non-negligible fraction of triplet pairs decay on a timescale ≪10 μs (Fig. 3d–f). These losses are especially striking in nanoparticles comprising the compound with the most compact side chain, in which case triplet pairs also form the fastest. This finding is consistent with pioneering work on covalently-tethered pentacene dimers where more rapid triplet pair formation has been generally observed to correlate with more rapid triplet pair decay.^3^,^81^ Such losses are also strikingly reminiscent of those observed early on in aggregates of the carotenoids zeaxanthin^25^ and astaxanthin.^103^

**Fig. 4:**
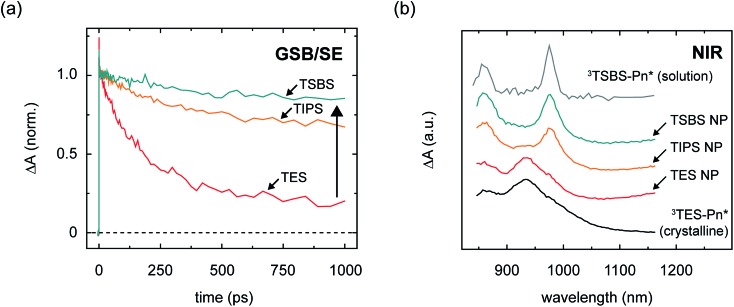
Transient kinetics and spectra of different singlet fission intermediates in amorphous pentacene derivative nanoparticles. (a) Transient absorption kinetics of TSBS-, TIPS-, and TES-pentacene nanoparticles in the visible spectral region. The transient absorption kinetics were obtained at a wavelength associated with overlapping ground-state bleach and stimulated emission features. The data were normalized to the maximum signal amplitude in the vicinity of *ca.* 3–50 ps. (b) Triplet photoinduced absorption spectrum of solution-phase TSBS-pentacene, transient absorption spectra of the nascent triplet pair intermediates in amorphous TSBS-, TIPS-, and TES-pentacene nanoparticles, and triplet photoinduced absorption spectrum of a crystalline film of TES-pentacene. The spectra have been offset for clarity of presentation.

In order to quantitatively evaluate the losses in the amorphous pentacene derivative nanoparticles, we performed transient absorption measurements in the visible spectral region (Section S11†). The visible spectral region is particularly advantageous for this purpose because the ground-state bleach feature can be used to evaluate the extent to which triplet pairs relax to the ground state, and assay losses incurred over the course of singlet fission. Fig. 4a shows the time evolution of the ground-state bleach feature and shows that substantial losses are apparent at long times in the nanoparticles comprising the compact side chain pentacene derivative; on the other hand, increasing the bulkiness of the side chain results in a drastic alleviation of these losses.^104^ Assaying the losses at a timescale of 1 ns, which is roughly the timescale of triplet pair separation in the amorphous pentacene derivative nanoparticles,^18^,^105^ we determine a long-lived triplet pair yield of 17, 64, and 81% for the amorphous TES-, TIPS-, and TSBS-Pn nanoparticles, respectively, which suggest potential independent triplet yields of 34, 128, and 162%, respectively (Table 2).

**Table 2:** Fraction of long-lived triplet pairs in amorphous pentacene derivative nanoparticles *via* ground-state bleach analysis^*a*^

Compound	*Φ*_TT_@1 ns (%)	Potential *Φ*_T_1__ (%)
TES-Pn	17	34
TIPS-Pn	64	128
TSBS-Pn	81	162

^*a*^The fraction of long-lived triplet pairs (*i.e.*, those with a lifetime ≫ 1 ns) were determined by taking the ratio of the ground-state bleach area at a timescale of 1 ns to that at 3–5 ps, 5–10 ps, and 10–30 ps for the amorphous TES-, TIPS-, and TSBS-pentacene derivative nanoparticles, respectively.

Correlated with the alleviation of the triplet pair losses, Fig. 4b shows that with increasing side chain bulkiness the intermediate time-delay photoinduced absorption spectra appear more and more like the triplet–triplet absorption spectrum of the isolated chromophore. Thus, losses in the amorphous nanoparticles are circumvented as the triplets comprising the triplet pair have spectra that look more like isolated-chromophore triplets. The TES- and TSBS-Pn nanoparticles, in fact, exhibit two distinct triplet photoinduced absorption bands, one which resembles isolated-chromophore triplets, and the other which is redshifted with respect to isolated-chromophore triplets and better resembles that of triplet excitons photogenerated in crystalline material (Section S13†).

Fig. 3d–f and 4b additionally show that, perhaps counterintuitively, increasing side chain bulkiness does not continuously vary the near-infrared triplet pair photoinduced absorption band. Rather, side chain bulkiness changes the relative amount of two distinct triplet pair photoinduced absorption bands, indicating that side chain bulkiness varies the relative amount of two distinct triplet pair populations.^106^ This is especially obvious when considering the intermediate-time delay transient near-infrared spectrum of the TIPS-Pn nanoparticles (Fig. 3e). Given that we can accurately model the intermediate-time delay spectrum of the TIPS-Pn nanoparticles using the intermediate-time delay spectra of the TSBS- and TES-Pn nanoparticles (Section S14†), we conclude that two distinct triplet pair populations are present in the amorphous pentacene derivative nanoparticles. Combined with our understanding of how the photoinduced absorption spectral changes are correlated with losses in these samples, we assign the redshifted photoinduced absorption to short-lived triplet pairs (TT_S_) and the photoinduced absorption that resembles that of isolated-molecule triplets to long-lived triplet pairs (TT_L_).

**Fig. 5:**
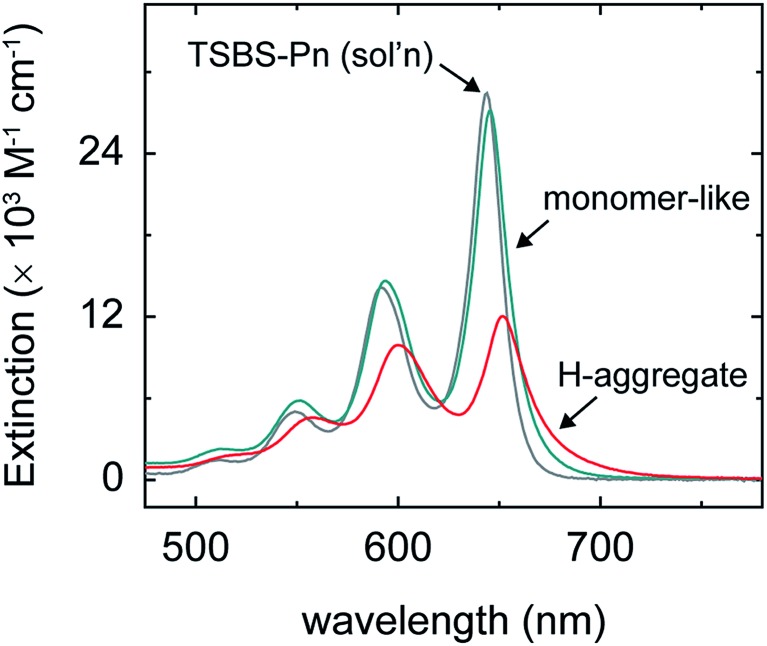
Extinction spectrum of a dilute solution of TSBS-pentacene in toluene plotted along with the “pure” extinction spectra associated with dimer pair sites in the amorphous pentacene derivative nanoparticles comprising weakly-coupled monomer-like and H-aggregated chromophores.

The discovery of distinct short- and long-lived triplet pair populations in the amorphous pentacene derivative nanoparticles enables us to learn more about the nature of the parent singlet excitons from which the triplet pairs originate. As shown above, the (nascent) triplet pair populations in these samples are accurately modeled as linear combinations. Given that we can also model the steady-state absorption spectra of the amorphous nanoparticle suspensions, which are representative of primary excitations or parent singlet excitons, as linear combinations with a similar modeling ratio as that determined for the triplet pair populations (Section S15†), we conclude that the two distinct triplet pair populations are generated *via* fission through two distinct parent singlet exciton populations. Making an additional assumption that the long-lived triplet pair yields (Table 2) can be used to derive the absolute population fractions in the different samples,^107^ we can obtain the pure spectra of the parent singlet excitons through a linear equation analysis (Fig. 5 and Section S16†). The spectra resulting from the analysis indicate that there are two types of parent singlet excitons leading to singlet fission in the amorphous pentacene derivative nanoparticles: (i) one comprising weakly-coupled chromophores that exhibit steady-state absorption spectra remarkably similar to isolated-chromophore singlet excitations (*i.e.*, S_1_S_0_ (m)), and (ii) another comprising weakly-coupled chromophores with steady-state absorption spectra that exhibit signatures of H-aggregation (*i.e.*, S_1_S_0_ (H)). Thus we show how side chain bulkiness sensitively varies the relative fraction of weakly-coupled chromophores in the amorphous pentacene derivative nanoparticles that adopt either a monomer-like or H-aggregate packing arrangement. Due to the lack of long-range order in amorphous material, singlet fission is thought to occur at dimer pair sites with packing arrangements suitable for singlet fission.^20^,^32^,^61^ Based on these results, we posit that the two sets of chromophores that adopt either H-aggregate or monomer-like packing arrangements are representative of the dimer pair sites responsible for short- and long-lived nascent triplet pair populations, respectively.

We can shed additional insight into the nature of the molecular packing at the different dimer pair sites by carefully evaluating the near-infrared photoinduced absorption spectra associated with the different triplet pairs. As shown above, side chain bulkiness sensitively varies the relative fraction of the two distinct dimer pair sites and, as a result, the relative fraction of the two distinct nascent triplet pair populations. The near-infrared photoinduced absorption of the short-lived triplet pair population was shown to be redshifted with respect that of the long-lived triplet pair population (Fig. 4b). The near-infrared triplet pair photoinduced absorption spectrum exhibits a redshifted triplet photoinduced band because of considerable orbital overlap between molecules, which is known to sensitively influence this particular transition (Section S17†), while the near-infrared triplet pair photoinduced absorption of the TSBS-Pn nanoparticles, which looks like isolated chromophore triplet excitations, reveals the absence of orbital overlap between molecules.

To conclude this section, the dimer pair sites where the short- and long-lived nascent triplet pairs are populated show some evidence for the presence and absence of orbital overlap, respectively. We therefore assign the molecular packing as contact and non-contact, respectively. We note that a recent theory study sheds additional insight on how molecular packing and intermolecular coupling influences these transient absorption signals.^108^

### Nature and dynamics of the triplet pair populations

**Fig. 6:**
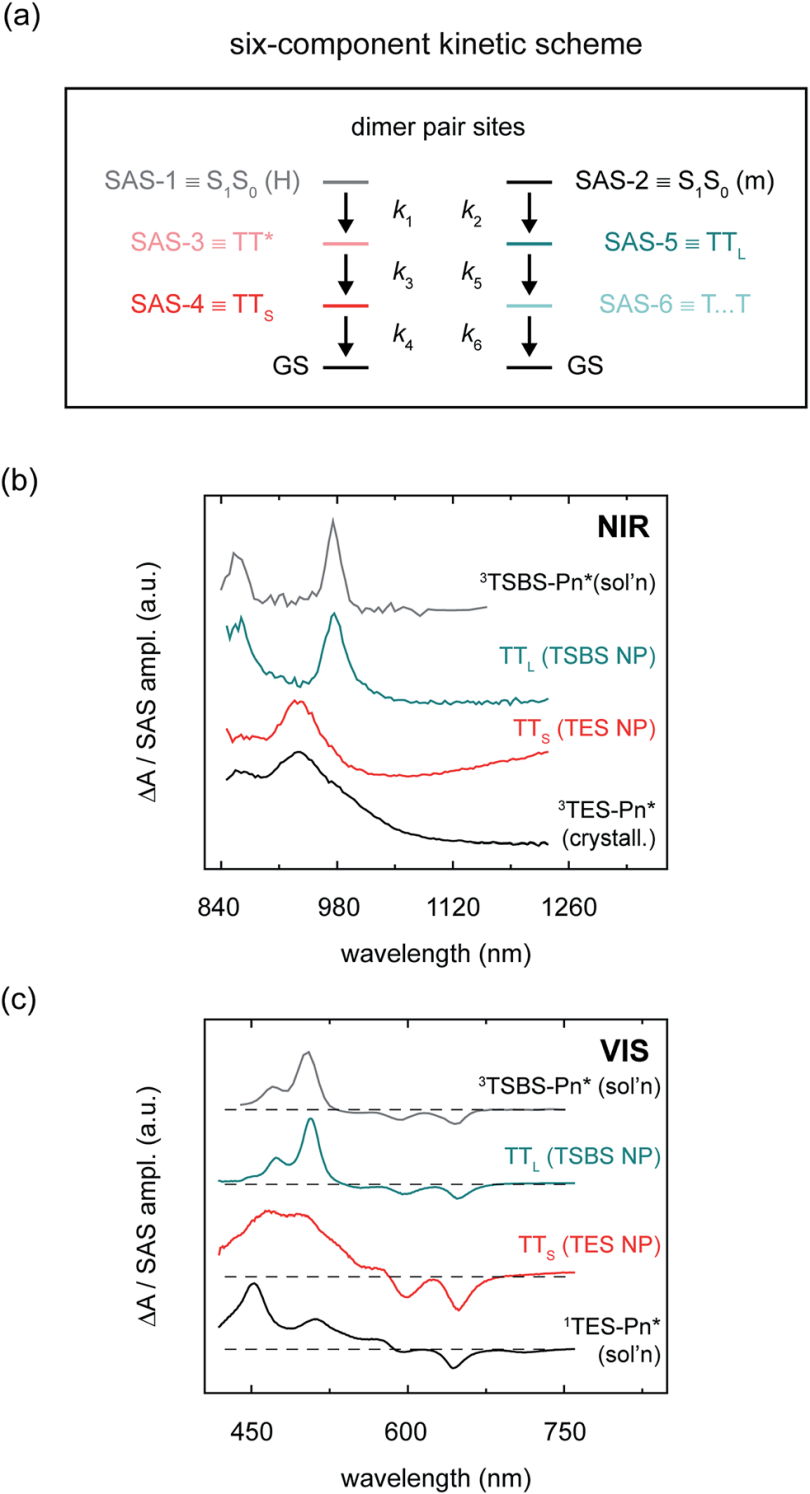
(a) Six-component kinetic scheme used for global and target analysis of amorphous pentacene derivative nanoparticles. For each set of sub-populations, the state corresponding to each component is listed alongside each SAS-n label. (b) Near-infrared species-associated spectra of short- and long-lived triplet pairs compared with solution-phase TSBS-Pn and crystalline TES-Pn triplet photoinduced absorption spectra. (c) Visible species-associated spectra of short- and long-lived triplet pairs compared with solution-phase TSBS-Pn triplet and TES-Pn singlet photoinduced absorption spectra.

Having identified two distinct sets of sub-populations in the amorphous pentacene derivative nanoparticles, we are now in a position to better understand their nature and dynamics. To do this, we must first disentangle how the different sub-populations contribute to the transient absorption signal. As shown above, the measured transient absorption data are well fitted by a linear combination of the spectrotemporal response of the two sets of sub-populations. In the presence of such sample heterogeneity, direct interpretation of the transient data provides only limited physical insight. To overcome this limitation, we performed a global and target analysis of the transient absorption data according to a six-component kinetic scheme (Fig. 6a). Global and target analyses are powerful approaches capable of disentangling the spectral and dynamical characteristics of overlapping populations.^109^,^110^ That is, a global target analysis derives “pure” spectra and kinetics for the individual sub-populations from the “mixed” spectrotemporal dataset. Global target analysis has been applied to model singlet fission dynamics of a number of chemical and material systems, including those that are disordered.^3^–^6^,^50^,^54^,^83^,^86^,^111^–^117^ Unlike previous models of singlet fission dynamics in disordered systems which have exclusively accounted for sequential dynamics (and have thus ignored potential sample heterogeneity),^3^,^4^,^6^,^25^,^68^,^81^–^83^,^85^,^91^,^114^,^117^,^118^ the six-component kinetic scheme presented here accounts for the parallel decay of two sets of sub-populations. Parallel decay pathways can especially be manifested in systems where multiple packing arrangements or conformations exist within the potential energy landscape^119^,^120^ and additionally where the underlying sub-populations exhibit overlapping ground-to-excited-state absorption spectra (see *e.g.*Fig. 5).

As shown above, side chain bulkiness provides a sensitive means in which to control the relative fraction of two distinct dimer pair sites in the amorphous pentacene derivative nanoparticles comprising monomer-like and H-aggregated chromophores (and thus control the relative fraction of the two distinct sub-population sets). In the six-component kinetic scheme, four of the six components account for the two distinct sets of sub-populations generated at these dimer pair sites, *i.e.*, short- and long-lived parent singlet excitons and nascent triplet pairs (labelled S_1_S_0_ (m), S_1_S_0_ (H), TT_S_, and TT_L_). The final two components account for dynamics of the different nascent triplet pair populations. One component accounts for the dissociation (or spatial separation) of long-lived nascent triplet pairs (*i.e.*, T···T).^5^,^18^ The sixth and final component accounts for intermolecular structural relaxation that immediately follows the formation of short-lived nascent triplet pairs (*i.e.*, TT*). We provide a more detailed justification of the full six-component kinetic scheme in the ESI (Section S18);† the primary results of the analysis are displayed in Fig. 6b and c and Table 3, and the complete set of species-associated spectra obtained for the different samples are presented in Section S19.†

**Table 3:** Six-component global target analysis of transient absorption of amorphous pentacene derivative nanoparticles (all time constants in units of ps)^*a*^ ^,^^*b*^

Compound	Dimer pair sites					
	H-aggregate			Monomer-like		
	*τ*_S_1_S_0_(H)_	*τ*_TT*_	*τ*_TT(S)_	*τ*_S_1_S_0_(m)_	*τ*_TT(L)_	*τ*_T···T_
TES-Pn	≤0.1	13	150	0.4	640	≫8000
TIPS-Pn	0.2	15	200	1.1	960	≫8000
TSBS-Pn	≤0.1	10	310	1.7	2300	≫8000

^*a*^As described in detail in the main text, the components SAS-1, SAS-3, and SAS-4 are assigned to the H-aggregate sub-populations S_1_S_0_ (H), TT*, and TT_S_, respectively, and the components SAS-2, SAS-5, and SAS-6 are assigned to the monomer-like sub-populations S_1_S_0_ (m), TT_L_, and T···T, respectively.

^*b*^Nanosecond transient absorption measurements indicate that spatially separated triplets exhibit a lifetime of *ca.* 10 μs (Section S20), *i.e.*, which is essentially equivalent to that of isolated-chromophore triplet excitations (Section S10).

We first discuss the microscopic rate constants associated with each individual sub-population derived from the global target analysis, which leads to one of the primary results of this work—triplet pairs that form quickly are directly correlated with those that decay quickly, while triplet pairs that form more slowly are directly correlated with those that are much longer lived. As can be seen in Table 3, parent singlet excitons formed at sites comprising H-aggregate dimer pairs, *i.e.*, SAS-1 or S_1_S_0_ (H) in the global target analysis, decay faster as compared with parent singlet excitons formed at dimer pair sites comprising monomer-like chromophores, *i.e.*, SAS-2 or S_1_S_0_ (m). Table 3 also shows that nascent triplet pairs populated at sites comprising H-aggregate dimer pairs have a very short lifetime. Their lifetime is evident through the time constant associated with SAS-4, or TT_S_, which ranges from *ca.* 150–300 ps for the different samples.^121^ Such a short lifetime serves to explain the extensive losses observed on the nanosecond timescale as a result of this sub-population (Fig. 4a and Table 2).

In contrast, the nascent triplet pairs populated at dimer pair sites comprising monomer-like chromophores spatially separate on a nanosecond timescale (*i.e.*, SAS-5 or TT_L_) and thereafter exhibit a lifetime ≫ 8 ns, well beyond the timescale of the femtosecond transient absorption measurement (*i.e.*, SAS-6 or T···T). In fact, nanosecond transient absorption measurements evidence that these triplet excitations recover the lifetime of the isolated-chromophore triplets; that is, they exhibit a lifetime of *ca.* 10 μs independent of side chain bulkiness (Section S20†). Thus, in the amorphous pentacene derivative nanoparticles side chain bulkiness is an effective means in which to inhibit the close approach of two molecules such that the triplet pairs begin to exhibit properties of isolated-chromophore triplet excitations.

Two additional results relevant to the dynamics of nascent triplet pairs emerge from the analysis. The first result pertains to the separation (or dissociation) of long-lived nascent triplet pairs (*i.e.*, *τ*_TT(L)_). Namely, Table 3 shows that the timescale of triplet pair separation in the amorphous nanoparticles increases in the series TES-Pn < TIPS-Pn < TSBS-Pn. This can be rationalized by considering that the timescale of triplet transfer, which is extremely sensitive to orbital overlap,^100^,^122^ is expected to increase as mass density decreases. We showed above that the average effective mass density decreases in the amorphous nanoparticles in the order TES-Pn > TIPS-Pn > TSBS-Pn. Thus, our observation that triplet pair separation (and by extension, triplet transfer^5^,^18^–^20^,^75^) is the slowest in the amorphous TSBS-Pn nanoparticles (and *vice versa* for the amorphous TES-Pn nanoparticles) is wholly consistent with the aforementioned trend in average effective mass density. The second result pertains to the intermolecular structural relaxation step that follows the formation of short-lived nascent triplet pairs (*i.e.*, *τ*_TT*_). The *τ*_TT*_ time constant, which we attribute to intermolecular structural relaxation following the formation of short-lived nascent triplet pairs, can be interpreted as arising from the formation of a state having a significantly lower energy than that of either the parent singlet exciton or the separated triplet pair. Such a scenario would initially confer a substantial amount of excess electronic energy to the system, which necessarily would deposit itself into the nuclear kinetic energy degrees of freedom.^123^ The subsequent transfer of this energy to the surrounding environment can lead to a two-step formation process exactly analogous to that proposed for excimer excitons,^124^,^125^ which has been attributed to intermolecular structural relaxation.^126^–^129^ That is, the molecules comprising the exciton exhibit subtle changes in their relative intermolecular geometries so as to result in slight differences in their electronic structure and, in turn, transient absorption spectra.^130^,^131^

The observation that the time constant associated with intermolecular structural relaxation following the formation of short-lived nascent triplet pairs is essentially independent of side chain bulkiness (Table 3) is consistent with a mechanism in which excess nuclear kinetic energy of the excitonic molecular pair is deposited into the surrounding molecules, as this would depend on the number of nuclear degrees of freedom of both the system and environment. Increasing side chain bulkiness (*i.e.*, with the addition or removal of a few atoms from the side chain) would contribute a relatively small change in the additional number of nuclear degrees of freedom, and so is expected not to substantially influence the kinetics of this process. Why the state energy of short-lived triplet pairs would be relaxed appreciably with respect to that of long-lived triplet pairs is expounded upon in the following section.

We next seek additional insights into the nature of the different nascent triplet pair populations through a detailed analysis of their species-associated spectra. The “pure” near-infrared spectra derived in the six-component global target analysis for the short- and long-lived nascent triplet pair populations are displayed in Fig. 6b. These spectra further evidence that, with increasing side chain bulkiness, nascent triplet pairs exhibit properties of isolated-chromophore triplets. Fig. 6b shows that, consistent with our observations in the first section of this paper, the origin band of the triplet near-infrared absorption peaks at *ca.* 935 and 980 nm for the short- and long-lived nascent triplet pair populations, respectively. That is, compact side chains cause the triplet near-infrared absorption to redshift, whereas bulky side chains prevent the close approach of two molecules such that the triplet photoinduced absorption band strongly resembles that of isolated-chromophore triplets (Fig. 4b). Fig. 6c presents additional evidence supporting the interpretation that bulky side chains prevent the close approach of two molecules, and that this promotes a triplet pair that exhibits properties of isolated-chromophore triplets. Fig. 6c shows that, in the visible spectral region, the transient absorption of nascent triplet pairs populated at dimer pair sites comprising monomer-like chromophores very much resembles that of isolated-chromophore triplets. Fig. 6c also shows that, quite surprisingly, the visible transient absorption of short-lived triplet pairs does not resemble that of crystalline triplets as we saw above in the near-infrared spectral region (Fig. 4b) but rather better resembles that of isolated-chromophore singlet excitations. Taken together, these results indicate that the photoinduced absorption spectrum of short-lived triplet pairs in the near-infrared exhibits a transition clearly reminiscent of crystalline triplet excitations, whereas the photoinduced absorption of the short-lived triplet pairs in the visible spectral region more strongly resembles that of isolated-chromophore singlet excitations.

**Fig. 7:**
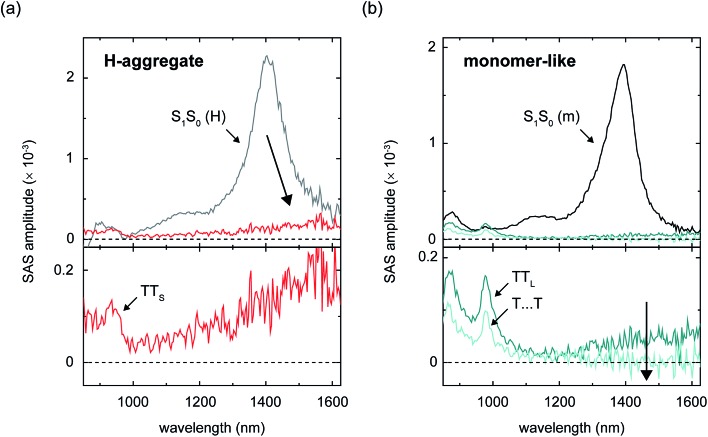
Near-infrared species-associated spectra derived from a six-component global target analysis of amorphous TIPS-Pn films associated with the set of parent singlet excitons and nascent triplet pairs populated at dimer pair sites comprising chromophores that are (a) H-aggregated and (b) monomer-like. The species-associated spectrum for the short-lived nascent triplet pair preceding intermolecular structural relaxation (*i.e.*, SAS-3 or TT*) has been omitted for clarity. The large arrows highlight the signal amplitude in the vicinity of *ca.* 1600 nm that is apparent in the nascent triplet pair populations, but not in the spatially separated triplet pairs.

To further elucidate the electronic structure of the different nascent triplet pairs, we performed transient absorption measurements on amorphous films of TIPS-Pn. Because water absorbs strongly above 1400 nm,^132^ we are unable to measure spectra beyond this wavelength in the aqueous nanoparticle suspensions; thus, amorphous films^133^,^134^ were investigated which enable us to measure the transient absorption spectra (and probe the characteristics of the triplet pair populations) over an extended spectral range slightly beyond *ca.* 1600 nm.^135^Fig. 7 displays the near-infrared species-associated spectra for the parent singlet exciton and nascent triplet pair populations in amorphous TIPS-Pn films derived according to the same six-component global target analysis used to model the transient absorption of the amorphous nanoparticle suspensions (Fig. 6a). The complete set of species-associated spectra for the films and time constants derived from the global target analysis are reported in Section S21† (see also footnote ref. 136). Critically, Fig. 7 shows that as parent singlet excitons fission into nascent triplet pairs, the photoinduced absorption associated with parent singlet excitons with an origin band that peaks at *ca.* 1400 nm (see Section S5†) transitions to a less intense, featureless, broadened, and redshifted feature centered at *ca.* 1600 nm which is apparent in the photoinduced absorption spectra of both short- and long-lived nascent triplet pair populations. We previously showed that these features do not result from either charge carriers or photodimers,^18^ and as such are intrinsic to the electronic structure of the different triplet pair populations.

Fig. 6b and c and 7 thus highlight the so-called “dual singlet-triplet optical character” of triplet pairs, originally reported in concentrated solutions of a tetracene derivative^88^ and nanoparticle suspensions of pentacene derivatives,^5^ that has also been observed in covalently-tethered tetracene and pentacene pairs.^3^,^6^,^81^ A simple, coherent and unifying explanation of the origin of this dual singlet-triplet optical character, however, has yet to be provided.

### Exciton theory description of triplet pairs

We can use exciton theory to account for the seemingly conflicting observations presented above in Fig. 6 and 7. This simple, qualitative description provides a satisfactory explanation of the dual singlet-triplet optical character observed for triplet pairs.^3^,^5^,^6^,^81^,^88^ Namely, in Fig. 6b and c we saw that short-lived nascent triplet pairs exhibited a prominent triplet photoinduced absorption band in their near-infrared transient absorption spectra, yet their transient visible absorption more strongly resembled that of isolated-chromophore singlet excitations. Furthermore, in Fig. 7 we noted the simultaneous observation of photoinduced absorption features in the transient near-infrared absorption that can be associated with triplet excitations and a weak, structureless, broadened and redshifted photoinduced absorption in the vicinity of the parent singlet induced absorption.

We propose that the same five electronic configurations generally applied to calculate singlet fission rates^9^,^10^—that is, exciton-resonance (ER), charge-resonance (CR), and doubly-excited (D) configurations (see Section S22†)—are additionally capable of describing the electronic structure of singlet fission intermediates, *i.e.*, correlated triplet pair states. In fact, simulations of molecular electronic structure have used these same five two-molecule electron configurations to describe excimer exciton emission in molecular crystals.^137^ For calculations of the electronic structure of collective excitations (involving two molecules), that is, in addition to the electronic states describing the isolated molecule, it is necessary to include all five electronic configurations available to the dimer pair. According to this description of triplet pairs and their spectra, we can explain the photoinduced absorption of nascent triplet pairs (Fig. 6b and c, and 7) as overall singlet excitons that have appreciable configuration mixing between the five two-molecule electronic configurations identified above, heavily weighted toward doubly excited D configurations. It is notable that the amplitude of the long-wavelength feature in short-lived nascent triplet pairs is increased in comparison with long-lived nascent triplet pairs (Fig. 7), which would suggest a higher weighting of ER configurations in the description of its wavefunction. We further find that spatially separated triplet pairs more closely resemble isolated-chromophore triplet excitations than long-lived nascent triplet pairs (Fig. 7b), a result consistent with *ab initio* work indicating that triplet pairs comprise essentially 100% D configurations as they spatially separate.^66^,^138^

We can explain the presence or absence of ER configurations in the electronic structure of nascent or spatially separated triplet pairs, respectively, by considering the spatial extent of the triplet pair in its different forms. That ER configurations (*i.e.*, derived from isolated-chromophore singlet excitations) would contribute to the electronic structure of nascent triplet pairs is sensible in that ER configurations contribute a stabilizing resonance interaction to the overall description of the triplet pair. It makes sense then that this stabilizing resonance interaction is possible only immediately after the triplet pair has formed when the excitation energy comprising its electronic wavefunction is spatially proximate. The case discussed above contrasts to spatially separated triplet pairs in which the electronic excitations are not spatially proximate. In that case stabilizing ER resonance interactions contribute negligibly to the wavefunction because they are mediated by orbital overlap and therefore the interaction diminishes steeply with intermolecular separation.

This hypothesis regarding the contribution of ER configurations as a function of separation of the triplet excitations has significant implications for understanding singlet fission. First, it suggests that nascent triplet pairs cannot be considered to comprise two independent, yet interacting triplet excitations. This viewpoint is only appropriate once the excitations have separated spatially. Second, although it has been suggested that separating the singlet fission process into distinct steps is artificial,^9^,^10^ the results reported here (and elsewhere, see *e.g.*ref. 5) provide strong validation that the singlet fission process can indeed be separated into three distinct steps of triplet pair formation, dissociation, and decoherence. The critical distinction made clear in the present work is that the triplet pair electronic wavefunction is not formally separable until after it has dissociated. In other words, parent singlet excitons and nascent triplet pairs each comprise a single electronic (excitonic) wavefunction that is not separable, whereas separated triplet pairs that exhibit properties of independent triplet excitations comprise a single, but separable electronic wavefunction (even though its spin eigenfunction is quantum mechanically entangled across both excitations). It is not until the spatially separated triplet pairs have decohered that one can consider the triplet excitations as comprising two independent particles with formally separable (independent) electronic and spin eigenfunctions.^12^

An additional aspect of the electronic structure of the nascent triplet pairs that warrants discussion is how molecular packing influences their state energy. As highlighted above, the global target analysis requires an additional component to accurately model the data, which we assign to a short-lived triplet pair population with a significantly relaxed energy as compared with that of parent singlet excitons, long-lived or separated triplet pairs. We recently identified similar dynamics and changes in transient spectral signatures associated with excimer relaxation, in which the state energy of the resultant excimer exciton is significantly reduced with respect to the parent exciton.^131^

One way to explain the low triplet pair state energy is that the ground-state molecular packing causes the triplet pair states to couple strongly. Smith and Michl suggested a simple criterion, for example, that if the singlet, triplet, and quintet triplet pair states are roughly degenerate (within a few cm^–1^) that the triplet pair will resemble isolated-chromophore triplets and will readily dissociate, whereas if the singlet, triplet, and quintet triplet pair states exhibit drastically different energies (*i.e.*, of the order of thousands of cm^–1^) then the triplet pair will not resemble isolated-chromophore triplets and will not readily dissociate.^9^ The results of this work are, in this respect, consistent with this suggestion. Namely, we observe long-lived nascent triplet pairs that strongly resemble isolated-chromophore triplet excitations and that dissociate, and we also observe short-lived nascent triplet pairs that do not resemble isolated-chromophore triplets and that do not dissociate (Fig. 6b and c, 7, and Table 3). Additional discussion of this possibility can be found in the ESI (Section S23).†

Another interpretation is that the nascent triplet pair has relaxed to an excimer geometry and that this has changed its state energy considerably. Excimer excitons are known to have significantly reduced energies with respect to their parent excitons.^139^ Thus, excimer relaxation could cause the energy of the short-lived nascent triplet pair to be significantly reduced with respect to parent singlet excitons, long-lived or separated triplet pairs. In addition to a significantly reduced state energy (as inferred from the additional component required in the global target analysis), the spectral signatures of short-lived nascent triplet pairs further evidence that they may have relaxed to an excimer geometry. Namely, we observe broad and structureless spectral features in both visible and near-infrared transient absorption spectra of these populations (Fig. 6c and 7a). Broad and structureless photoinduced absorption features are classical examples of molecular ‘excimer’ excited-state absorption.^61^,^68^,^140^ As already mentioned, the two-step dynamics we observe for the short-lived nascent triplet pairs is strikingly similar to the two-step excimer relaxation reported for several classical molecular ‘excimer’ forming materials that has also been associated with structural relaxation.^124^–^129^ It is not unreasonable to conclude then that excimer relaxation, known to be detrimental to singlet fission,^61^,^68^,^90^ is hindered by side chain sterics, which promote the formation of long-lived triplet pairs and thus high independent triplet yields (with a similar thesis drawn in a recent, related work^61^).

It is an intriguing result that the short-lived nascent triplet pair strongly resembles isolated-chromophore singlet excitations, which indicates a significant incorporation of ER configurations (*i.e.*, derived from isolated-chromophore singlet excitations) in its electronic structure that may additionally act to stabilize this state. We also showed that long-lived nascent triplet pairs are populated at dimer pair sites comprising monomer-like chromophores where molecules pack less cofacially and exhibit a non-contact packing arrangement whereas short-lived nascent triplet pairs are populated at dimer pair sites comprising chromophores exhibiting signatures of a more cofacial, contact-type packing arrangement (Sections S16 and S17†). We further speculate that, as a result of this latter molecular packing arrangement, that either the energies of the triplet pair states of different spin multiplicities differ significantly or the molecules are ‘pre-associated’ in a manner that promotes the parent singlet exciton to relax to a triplet pair in an excimer geometry (for an explanation of the term ‘pre-associated’, see *e.g.*ref. 141). It is interesting that neither of these scenarios are generally encountered in the equilibrium-structure crystalline form of 6,13-substituted derivatives of these materials (see ref. 5 and 18 and Section S13†). Thus, as we previously suggested,^20^ pentacene derivatives are especially versatile singlet fission chromophores, capable of quantitative singlet fission in the form of a variety of different chemical structures^5^,^18^,^20^ and solid-state phases.

**Fig. 8:**
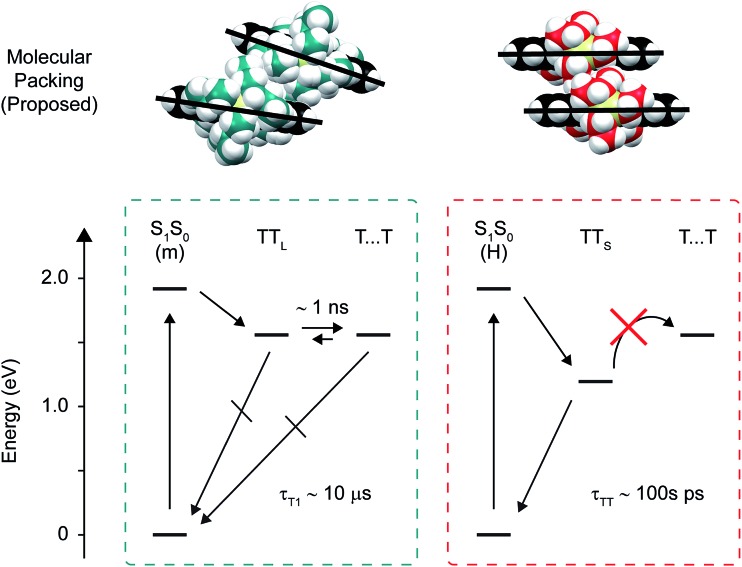
Proposed molecular packing and state diagram describing singlet fission in the amorphous pentacene derivative nanoparticles. (Upper panel) Schematic molecular packing structures proposed for the monomer-like and H-aggregate dimer pair sites, illustrating how more bulky side chains push the molecules farther apart. (Bottom panel) State diagrams depicting singlet fission at the monomer-like and H-aggregate dimer pair sites. The energy for the triplet pair was estimated as 2 × *E*_T_1__,^9^ or 1.56 eV, where *E*_T_1__ was taken to be 0.78 eV which is the energy Zirzlmeier *et al.* measured for the origin band of the phosphorescence spectrum of TIPS-Pn.^81^

Pentacene derivatives are exemplar singlet fission chromophores because they have an optimal energy-level structure, such that triplet pair formation is exoergic (see *e.g.* footnote ref. 142). This means that small couplings are capable of promoting rapid (picosecond and sub-picosecond) formation of triplet pairs and that triplet pairs can be produced with high efficiencies (Table 1). The key to using these overall singlet states for efficient carrier multiplication is to ensure that they survive long enough after their production. We suggested that, with the right molecular packing, a triplet pair can form that has properties like two independent triplet excitations. While additional information on the exact molecular packing needed to avoid losses is highly desirable, we have shown in amorphous solids of pentacene derivatives that a co-facial (H-aggregate) packing arrangement is largely responsible for these losses, and that side chain sterics can be used to inhibit the close approach of two molecules, which suppresses this packing arrangement, circumvents losses, and promotes overall highly-efficient singlet fission (Fig. 8).

### Mechanism of triplet pair decay

Having clarified many aspects of the nature and dynamics of the different triplet pair populations, we now proceed to shed more insight on their decay mechanism. In this section, we show that, in contrast to a number of other decay mechanisms that have been proposed, nascent triplet pairs decay to the ground state primarily *via* a highly effective nonradiative (internal conversion) process.

**Fig. 9:**
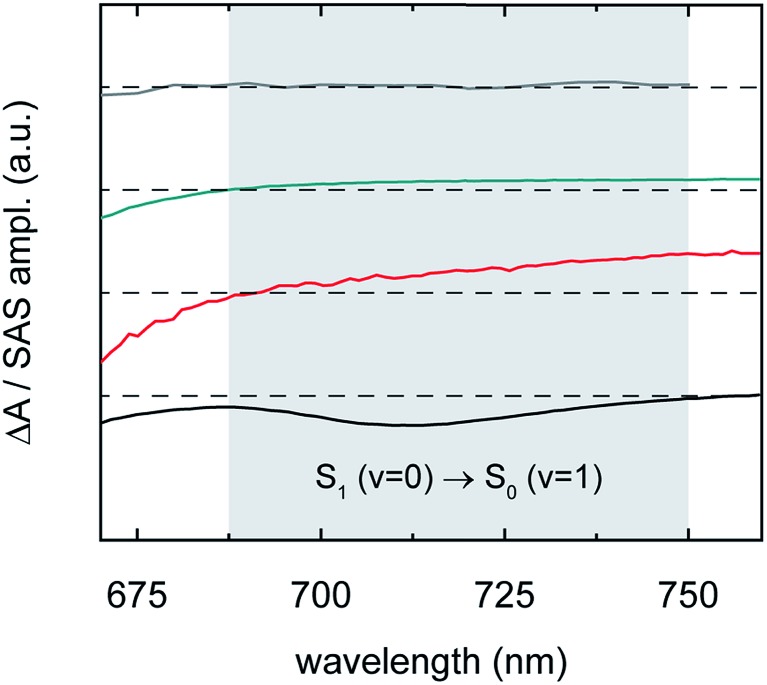
Transient absorption spectrum of isolated-chromophore TSBS-Pn triplet excitations (grey), species-associated spectrum of long-lived nascent triplet pairs derived from amorphous TSBS-Pn nanoparticles (cyan), species-associated spectrum of short-lived nascent triplet pairs derived from amorphous TES-Pn nanoparticles (red), and transient absorption spectrum of isolated-chromophore TSBS-Pn singlet excitations (black). The data, reproduced from Fig. 6c, are displayed highlighting a narrow range in the visible spectral region in the vicinity of the second vibronic (0–1) stimulated emission band of parent singlet excitons (grey region).

We first highlight that, in contrast to recent suggestions,^7^,^143^ nascent triplet pairs in pentacene derivative solids do not emit light, *i.e.*, radiative (emissive) decay is suppressed. Consistent with this interpretation we find that the transient spectra of nascent triplet pairs lacks a stimulated emission band. Fig. 9 shows, for example, the transient spectra presented in Fig. 6c over a narrow spectral window spanning the 0–1 vibronic stimulated emission band of parent singlet excitons; it is found that whereas the transient absorption spectrum of parent singlet excitons exhibits a prominent stimulated emission band, the transient absorption spectra of short-lived nascent triplet pairs, long-lived nascent triplet pairs, and isolated-chromophore triplet excitations do not. This is an especially interesting observation for the short-lived nascent triplet pairs, whose transient absorption spectra appear to otherwise very closely resemble that of isolated-chromophore singlet excitations (Fig. 6b and c and 7). Although ER configurations play a role in describing the electronic structure of nascent triplet pairs, these results indicate that their electronic structure is largely determined by D configurations such that nascent triplet pairs are not of the right symmetry to couple to the ground state and emit light. We note that the electronic structure of these trialkylsilylethynyl-substituted pentacenes (*E*_T_1__ = 0.78 eV;^81^*E*_TT_ ≈ 2 × *E*_T_1__ = 1.56 eV; and, in the present context, *E*_S_1_S_0__ ≈ 1.91 eV) does not support appreciable mixing of ER configurations and, in contrast to recent suggestions,^144^–^146^ does not support an S_1_S_0_ ↔ TT equilibrium.

Additional evidence indicating nascent triplet pairs do not emit light is found when comparing the results of steady-state fluorescence quantum yield measurements with fluorescence quantum yields estimated *via* transient absorption spectroscopy. More specifically, we find an excellent correlation between measured fluorescence quantum yields and those estimated according to the rates of decay of the parent singlet exciton population measured *via* transient absorption (Section S24†). Taken together, these results indicate that nascent triplet pairs are non-emissive and that all light emitted by the amorphous pentacene derivative nanoparticles originates from parent singlet excitons. Nascent triplet pairs thus must decay to the ground state *via* another mechanism nonradiatively.

**Fig. 10:**
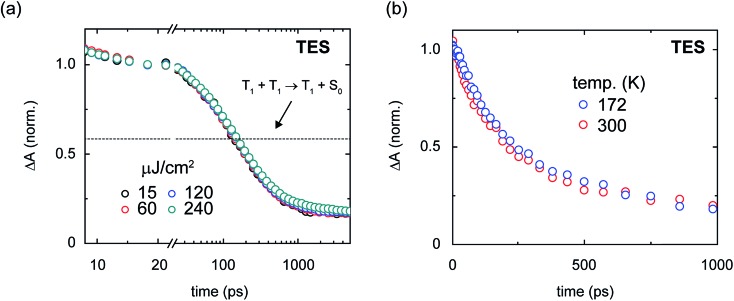
Fluence- and temperature-dependence of the transient absorption of the amorphous TES-pentacene nanoparticles. (a) Fluence-dependence of amorphous TES-pentacene nanoparticles. The data were normalized over the range from 15–25 ps. A single trace overlays the data indicating the asymptotic population amplitude expected for geminate triplet–triplet annihilation. (b) Temperature-dependence of amorphous TES-pentacene nanoparticles. Two measurements were performed at temperatures of *ca.* 172 and 300 K.

We can gain insight into how triplet pairs decay nonradiatively *via* excitation fluence- and temperature-dependent transient absorption measurements. Because the nonradiative decay of triplet pairs has been generally suggested to occur through recombination,^1^–^4^,^6^,^7^,^68^,^70^,^82^,^84^,^85^,^116^,^117^,^147^ or more precisely, an annihilation process, we first performed fluence-dependent transient absorption on the amorphous pentacene derivative nanoparticle suspensions to test for signs of bimolecular triplet–triplet annihilation. Fig. 10a shows the fluence-dependent transient absorption of the amorphous TES-Pn nanoparticles. We chose to study the amorphous TES-Pn nanoparticles initially because nanoparticles of this compound comprise largely short-lived nascent triplet pairs responsible for the losses observed in the amorphous pentacene derivative nanoparticle suspensions. Fig. 10a shows that bimolecular triplet–triplet annihilation can be ruled out as a nonradiative decay mechanism because the transient absorption of the amorphous TES-Pn nanoparticle suspensions is clearly independent of incident pump fluence. We find that the transient absorption of the amorphous TIPS- and TSBS-Pn nanoparticles also are largely independent of incident pump fluence (see ref. 18 and Section S25†), thus ruling out bimolecular triplet–triplet annihilation as a nonradiative decay mechanism in amorphous pentacene derivative nanoparticles more generally.

The emerging consensus that the decay of triplet pairs can be attributed to an annihilation process has its origins from the covalently-tethered dimer literature, where it has generally been presumed that triplet pairs decay nonradiatively in this manner. The nonradiative decay has been presumed to occur primarily *via* a geminate recombination or annihilation process because of the spatial confinement of triplet pairs on single covalently-tethered dimers.^1^–^4^,^6^,^7^,^68^,^70^,^82^,^84^,^85^,^117^,^147^ Given that there is no bimolecular, or nongeminate, triplet–triplet annihilation in the amorphous pentacene derivative nanoparticles on the timescale of the transient absorption measurements presented in this work, we can test this assertion by comparing how the transient absorption signal behaves with that expected for the case where geminate triplet–triplet annihilation is entirely responsible for the decay. In the event that geminate triplet–triplet annihilation is responsible for the decay, we can predict the transient absorption signal by considering a simple stoichiometric analysis for the geminate annihilation process (Section S26†). In brief, given that only triplet pairs are formed in the amorphous pentacene derivative nanoparticles (Table 1) and assuming that geminate triplet–triplet annihilation occurs through a high-lying triplet state, a simple stoichiometric argument indicates that geminate triplet–triplet annihilation cannot cause the transient absorption signal to decay by more than half its initial value. Fig. 9a shows that the transient absorption signal in the amorphous TES-Pn nanoparticles decays well below the asymptotic limit expected for two adjacent triplets annihilating. Thus, we can rule out geminate annihilation as a nonradiative decay mechanism for nascent triplet pairs. Perhaps most critically, the inability to ascribe the decay of the transient absorption signal to geminate triplet–triplet annihilation is fully consistent with the general picture developed in this work that the triplets comprising the nascent triplet pair cannot be considered independently.

In an attempt to measure the binding energy of the triplet pair, we additionally performed temperature-dependent transient absorption measurements on the amorphous pentacene derivative nanoparticles. Assuming that short-lived triplet pairs contribute to some extent to the long-time triplet pair yields and assuming kinetic competition between the measured time constants for the decay of the short-lived triplet pair and its dissociation of *ca.* 150 and 640 ps (Table 2), we could expect to observe a temperature dependence of the long-time triplet pair yield. Fig. 10b shows that the dynamics of nascent triplet pairs in amorphous TES-Pn nanoparticles are essentially invariant to temperature. This result indicates that additional factors may be preventing a direct measurement of the binding energy of the nascent triplet pair. Additional factors, such as structural and energetic disorder, which are likely present to a large degree in these amorphous solids, could, as has recently been suggested,^7^ facilitate triplet pair dissociation and obscure a direct measurement of the binding energy of the triplet pair. We note that the transient absorption of amorphous TSBS-Pn nanoparticles also is largely invariant of temperature (Section S27†), suggesting that structural and energetic disorder may be obscuring a direct measurement of the nascent triplet pair binding energy in this sample as well. An overlapping contribution from thermally-activated dissociation of long-lived triplet pairs may be an additional factor complicating a measurement of the nascent triplet pair binding energy in these particular samples.

The results presented here indicate that nascent triplet pairs are best represented as single entities, or non-separable states, that efficiently couple to the ground state *via* a highly effective nonradiative (internal conversion) process. How molecular packing influences triplet pair decay to the ground state^69^,^148^ therefore represents an important criterion impacting the longevity of triplet pairs, which should be considered and optimized for singlet fission to be realized in practical applications.

## Conclusion

In summary, we prepared amorphous nanoparticle suspensions of a series of trialkylsilyl-substituted pentacene derivatives and investigated the influence of side chain bulkiness on their singlet fission dynamics. While triplet pair formation was quantitative in nanoparticles of all compounds, we found that side chain bulkiness sensitively varied the relative population fraction of dimer pair sites where nascent triplet pairs either completely decayed back to the ground state (*i.e.*, quantitative losses) or completely dissociated into separated triplet pairs (*i.e.*, quantitative yields). In order to evaluate the singlet fission dynamics along with the nature of the different singlet fission intermediates, we found it necessary to account for the parallel decay pathways identified in the amorphous pentacene derivative nanoparticles *via* a global target analysis. A six-component kinetic scheme was developed to account for two distinct sets of parent singlet exciton and nascent triplet pair populations along with dynamics associated with the short- and long-lived nascent triplet pair populations (assigned to intermolecular structural relaxation and triplet pair separation, respectively). While nascent triplet pairs were found to exhibit “dual singlet–triplet optical character”, separated triplet pairs were found to strongly resemble isolated-chromophore triplet excitations. The former result indicates that nascent triplet pairs cannot be approximated as two independent electronic excitations, as is often invoked,^1^–^7^ but rather must be treated as a single entity. We proceeded to explain the dual singlet-triplet optical character and single-particle nature of the nascent triplet pair electronic wavefunction by invoking a simple exciton theory description. Finally, we found that fluence- and temperature-dependent transient absorption measurements further support the interpretation that triplet pairs must be considered as a single electronic excitation and additionally indicate that triplet pairs decay exclusively *via* a nonradiative internal conversion mechanism.

It is worthwhile to consider the implication of these results on losses in singlet fission. As we have shown, it is incorrect to view the nascent triplet pair as two individual and interacting triplets. The nascent triplet pair must be considered electronically as a single distinct particle (*i.e.*, a singlet exciton comprising a single, inseparable electronic wavefunction); we cannot consider the triplet pair electronically as two particles until the triplets comprising the triplet pair have spatially separated. This can be considered to be a realization, in some sense, of the suggestion that it is arbitrary to divide the singlet fission process into multiple steps,^9^ although we believe that the singlet fission process can indeed be divided into multiple steps.^5^,^12^ Viewing the singlet fission process in this framework, we have shown that molecular packing, or conformation, is an important property to consider in addition to triplet–triplet interactions, which have been a target of recent transient absorption^4^ and magnetic-based measurements.^117^,^149^–^151^ Our results show that it is important to consider how molecular packing arrangements (and more generally, molecular conformations) determine how effectively nascent triplet pairs couple to the ground state, a topic we consider to be of especial interest for future work.

We additionally showed that while side chain bulkiness may minimally impact initial triplet pair yields, they may have a drastic effect on long-time, independent triplet yields *via* their influence on molecular packing or conformation. Looking forward, it is interesting to consider that there may be a compromise between enough bulk to maintain high initial triplet pair yields and high long-time, independent triplet yields, but not so much so that triplet pair separation is slowed down appreciably and no longer competitive with internal conversion of the triplet pair to the ground state; in some sense similar to the concept of “kinetic redundancy” reported by Grätzel, Durrant and co-workers in the context of charge separation and recombination in dye-sensitized solar cells.^152^ Thus, judicious tailoring of side chain bulkiness may represent a promising synthetic approach to circumventing detrimental losses in singlet fission in future work.

This work represents an important step toward better understanding losses in singlet fission. It is hoped that the fundamental insights gained in this work serve to bring the phenomenon closer to its practical implementation.

## Experimental methods

### Pentacene derivatives

HPLC-grade (≥99%) TIPS- and TES-pentacene were purchased from Sigma-Aldrich and used as received. TSBS-pentacene was synthesized as reported in the literature.^153^

### Nanoparticle preparation

An 800 μM solution of each pentacene derivative in unstabilized tetrahydrofuran (THF; EMD Millipore, Billerica, Maryland) was prepared. 200 μL of the solution was rapidly injected into a 20 mL glass scintillation vial containing 9.8 mL of vigorously stirring distilled water. A 21 gauge disposable needle (BD, Franklin Lakes, New Jersey) and 1 mL disposable syringe (Henke-Sass Wolf, Tuttlingen, Germany) were used to inject the pentacene derivative/THF solution. The aqueous colloidal nanoparticle suspensions were concentrated by combining four 10 mL batches together and subjecting the total solution to rotary evaporation at 18 mbar and 35 °C for a period of *ca.* 40–45 min.

### Steady-state absorption spectroscopy

Absorption spectra were measured with an Agilent Cary 60 spectrophotometer (Agilent Technologies, Santa Clara, California).

### Femtosecond transient absorption spectroscopy

The femtosecond transient absorption spectrometer has been described in detail previously.^5^ Briefly, measurements were performed with a 1 kHz regeneratively amplified Ti:sapphire laser system (Coherent Libra, Santa Clara, California) that delivers ∼45 fs pulses at ∼800 nm with an average power of ∼4 W. Pump and probe beam paths were generated by placing a beamsplitter at the output of the laser amplifier. A large fraction of the power was used to drive an optical parametric amplifier (Light Conversion OPerA, Vilnius, Lithuania) to convert the 800 nm light to 645 nm, the pump wavelength used for the femtosecond transient absorption measurements. The pump and a small fraction of the power of the laser amplifier were directed towards a commercial transient absorption spectrometer (Ultrafast Systems Helios, Sarasota, Florida). The latter was used to generate a continuum in either the visible (*ca.* 420 to 760 nm) or near-infrared (*ca.* 850 to 1600 nm) spectral region. Optical filters were used to isolate the continuum from the 800 nm radiation. The relative pump and probe polarization was controlled with a combination of a *λ*/2 waveplate and polarizer in the probe beam path situated before the continuum generation crystal. Measurements were performed with pump and probe polarizations oriented at the magic angle at the sample. The nanoparticles suspensions were contained in a 2 mm path length glass spectrophotometer cell (Starna Cells, Inc., Atascadero, California) that included a stir bar to stir the solution over the course of the measurement. The optical density of the samples varied from *ca.* 0.35 to 0.45 at the excitation wavelength. The pump beam spot size was determined by placing a digital CCD camera (Thorlabs Inc., Newton, New Jersey) at focal plane of the probe in the region of pump and probe overlap and analyzing an image obtained using ThorCam software (Thorlabs Inc., Newton, New Jersey). The spot size determined in this manner was *ca.* 180 μm. Pulse energies were measured with an optical power sensor and meter (Coherent Inc., Santa Clara, California). The incident pump fluences for the different measurements are reported where appropriate.

### Nanosecond transient absorption spectroscopy

Nanosecond transient absorption spectroscopy was performed using a home-built laser flash photolysis instrument reported previously.^154^ Briefly, the excitation source was a ∼10 ns pulsed laser operating at 30 Hz generated by pumping a dye laser cavity (Photon Technology International, Edison, New Jersey) using a frequency-doubled Nd:YAG laser (Continuum, San Jose, California). For near-infrared spectroscopy, a pump wavelength and fluence of 649 nm and ∼100 μJ cm^–2^, respectively, were used for the measurements. The output of a tungsten halogen lamp (Spectral Products, Putnam, Connecticut) passing through a 700 nm longpass filter was used as the probe. Spectral resolution (10 nm effective bandwidth) was obtained by dispersing the probe using a monochromator (DK240, Spectral Products, Putnam, Connecticut). The dispersed probe beam was detected using an InGaAs photodiode (Thorlabs, Newton, New Jersey). Samples were prepared at a concentration of 2 × 10^–5^ M in toluene and were flowed over the course of the measurement.

For visible/near-IR spectroscopy, a pump wavelength and fluence of 605 nm and ∼200 μJ cm^–2^, respectively, were used for measurements of isolated-chromophore triplet photoinduced absorption spectra. The output of a tungsten halogen lamp (same as for near-infrared spectroscopy) was used as the probe. Spectral resolution of 10 nm effective bandwidth was obtained by dispersing the probe using a different monochromator (CM110, Spectral Products, Putnam, Connecticut). The dispersed probe beam was then detected using a Si photodiode (Thorlabs, Newton, New Jersey). Dielectric filters were used to block scatter from the pump. Several samples were prepared at a concentration of 2 × 10^–5^ M in toluene and were gently stirred over the course of the measurements. The resulting spectrum was the average of several different samples measured using short laser exposure times to avoid degradation.

## Author contributions

R. D. P., A. J. T., J. E. A., and G. D. S. conceived the project. R. D. P. designed and coordinated experiments. R. D. P. prepared the nanoparticle samples, performed the spectroscopy, and analyzed the data, with additional support from A. J. T., C. G., E. E. O., D. G. O., J. C. D., and G. S. D. G. E. P. prepared films and performed structural characterization. D. B. G. synthesized the materials. R. D. P. wrote the manuscript with input from all co-authors. J. B. A., Y.-L. L., D. S. S., J. E. A. and G. D. S. directed the research.

## Conflicts of interest

There are no conflicts to declare.

## Supplementary Material

Supplementary information
