# Supplementary material for: Striking the right balance of intermolecular coupling for high-efficiency singlet fission
Source: Chem Sci. 2018 Jun 1;9(29):6240–59. doi: 10.1039/c8sc00293b (PMC6062843; doi:10.1039/c8sc00293b)
Supplement: Supplementary file 1 [file SC-009-C8SC00293B-s001.pdf]

Electronic Supplementary Material (ESI) for Chemical Science.
This journal is © The Royal Society of Chemistry 2018

Supporting Information for

Striking the right balance of intermolecular coupling for high-efficiency singlet fission

Ryan D. Pensack,[a] Andrew J. Tilley,[b] Christopher Grieco,[c] Geoffrey E. Purdum,[d] Evgeny E. Ostroumov,[a] Devin B. Granger,[e] Daniel G. Oblinsky,[a] Jacob C. Dean,[a] Grayson S. Doucette,[c] John B. Asbury,[c] Yueh-Lin Loo,[d,f] Dwight S. Seferos,[b] John E. Anthony,[e] and Gregory D. Scholes[a]

[a]Department of Chemistry, Princeton University, Princeton, New Jersey 08544, USA.
[b]Department of Chemistry, University of Toronto, Toronto, Ontario M5S 3H6, Canada.
[c]Department of Chemistry, The Pennsylvania State University, University Park, Pennsylvania 16802, USA.
[d]Department of Chemical and Biological Engineering, Princeton University, Princeton, New Jersey 08544, USA.
[e]Department of Chemistry, University of Kentucky, Lexington, Kentucky 40506, USA.
[f]Andlinger Center for Energy and the Environment, Princeton University, Princeton, New Jersey 08544, USA.

Table of Contents:

S1. Supplementary methods

S2. Diameter of aqueous colloidal TES-, TIPS-, and TSBS-pentacene nanoparticles ranges between 80 and 90 nm

S3. Comparing the electronic absorption spectra of amorphous and crystalline films of TES-, TIPS-, and TSBS-pentacene with that of the nanoparticle suspensions indicates the nanoparticles are amorphous

S4. Variations in excitonic coupling and mass density explain trends in steady-state absorption spectra

S5. Isolated-chromophore singlet excitation and parent singlet exciton transient absorption spectra compare well

S6. Isolated-chromophore triplet excitation and nanoparticle long-time transient absorption spectra compare well

S7. Qualitative arguments can be made that the electronic couplings relevant to triplet pair formation are stronger for the H-aggregated chromophores even after accounting for the concentration of and singlet diffusion to fission sites

S8. Fluorescence lifetimes of dilute solutions of TES-, TIPS-, and TSBS-pentacene in toluene

S9. Parent singlet exciton lifetimes in amorphous TES-, TIPS-, and TSBS-pentacene nanoparticles

S10. Triplet lifetimes obtained for isolated chromophores in dilute toluene solutions of TES-, TIPS-, and TSBS-pentacene

S11. Transient visible absorption of TES-, TIPS-, and TSBS-pentacene nanoparticles

S12. Transient absorption of the pentacene derivative nanoparticles is not influenced by the presence of molecular oxygen

S13. Triplet excitons photogenerated in crystalline TES-pentacene thin films

S14. Transient absorption spectrum of intermediate population in TIPS-pentacene nanoparticles can be modeled with corresponding TES- and TSBS-pentacene data

S15. Modeling the extinction spectrum of the TIPS-pentacene nanoparticles returns similar population fraction as modeling the transient absorption spectra

S16. A linear equation analysis returns the pure spectra of the parent singlet excitons associated with the two distinct dimer pair sites

S17. Orbital overlap has a profound effect on the triplet near-infrared induced absorption

S18. Detailed justification of six-component kinetic scheme

S19. Complete set of species-associated spectra returned from a six-component global target analysis of the transient absorption of all samples

S20. Unimolecular lifetimes of triplet excitations in amorphous TES-, TIPS-, and TSBS-pentacene nanoparticle suspensions

S21. Complete set of species-associated spectra for the amorphous TIPS-Pn films and time constants derived from the global target analysis

S22. Five most basic two-molecule electron configurations

S23. How ground-state molecular packing could influence the triplet pair state energy

S24. Nascent triplet pairs do not emit light

S25. No bimolecular annihilation in amorphous TIPS- or TSBS-Pn nanoparticles

S26. Why geminate triplet-triplet annihilation does not explain losses

S27. Amorphous TSBS-Pn nanoparticles transient absorption is also temperature independent

S28. References

<u>Section S1:</u> Supplementary methods

Dynamic light scattering

Dynamic light scattering measurements were performed on a Malvern Instruments Zetasizer Nano ZS instrument equipped with a Helium Neon laser (λ = 633 nm).

Steady-state fluorescence spectroscopy

Steady-state fluorescence measurements were performed on a Horiba PTI QuantaMaster 400 spectrofluorometer (HORIBA Scientific, Edison, NJ). Fluorescence spectra were recorded with the slit widths of both excitation and emission monochromators set to 3.5 nm bandwidth (1.34 mm slit width). Scan parameters were set to 2 nm steps and 0.25 s integration time. Fluorescence measurements were made on solutions with an optical density of 0.10 or less contained in a 1 cm pathlength quartz cell (Starna Cells, Inc., Atascadero, CA). All reported fluorescence spectra were intensity corrected.

Time-correlated single photon counting

Time-resolved fluorescence measurements were made using a DeltaFlex time-correlated singlet photon counting (TCSPC) spectrometer (HORIBA Scientific, Edison, NJ). The excitation and emission wavelengths were 507 and 650 nm, respectively. The optical density of the sample at the maximum of the lowest-energy electronic transition was ~0.1 or less which corresponded to an optical density of ~0.005 or less at the excitation wavelength. The sample was contained in a 1 cm fluorometer cell (Starna Cells Inc, Atascadero, CA). The instrument response function was obtained by placing an aqueous Ludox colloidal silica suspension, which efficiently scatters the excitation light, into the spectrometer while keeping all settings equivalent to those used for the sample. The instrument response function exhibited a full-width at half maximum of ca. 200 ps, which is representative of the time resolution of the TCSPC spectrometer.

Section S2: Diameter of aqueous colloidal TES-, TIPS-, and TSBS-pentacene nanoparticles ranges between 80 and 90 nm

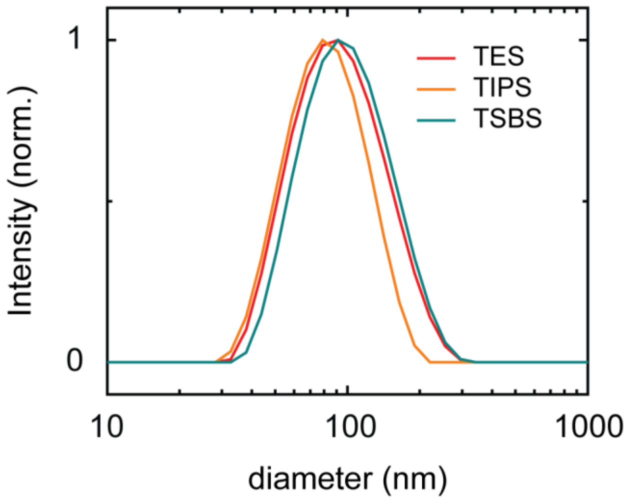

Figure S2. Results of dynamic light scattering measurements on aqueous colloidal TES-, TIPS-, and TSBS-pentacene nanoparticles. The measurements were performed at 25 ^{o}C. The Z-average values for each compound are: TES- (85 nm), TIPS- (75 nm), and TSBS-Pn (91 nm).

Figure S2 shows the results of dynamic light scattering measurements on the aqueous colloidal TES-, TIPS-, and TSBS-Pn nanoparticles that indicate diameters of ca. 85, 75, and 91 nm, respectively. Diameters ranging from ca. 80 – 90 nm for the aqueous colloidal pentacene derivative nanoparticles are in excellent agreement with diameters ranging from ca. 60 – 70 nm previously reported.[1,2] Critically, given that the diameter of the nanoparticles is much smaller than the wavelengths of light used to probe the nanoparticles in this work, optical artifacts associated with scatter are largely absent.

<u>Section S3:</u> Comparing the electronic absorption spectra of amorphous and crystalline films of TES-, TIPS-, and TSBS-pentacene with that of the nanoparticle suspensions indicates the nanoparticles are amorphous

We evaluated the solid-state order of the pentacene derivative nanoparticles by comparing their electronic absorption spectra with that of the respective material in the form of dilute toluene solutions, “as-cast” films, and solvent-vapor annealed films (**Figure S3.1**).

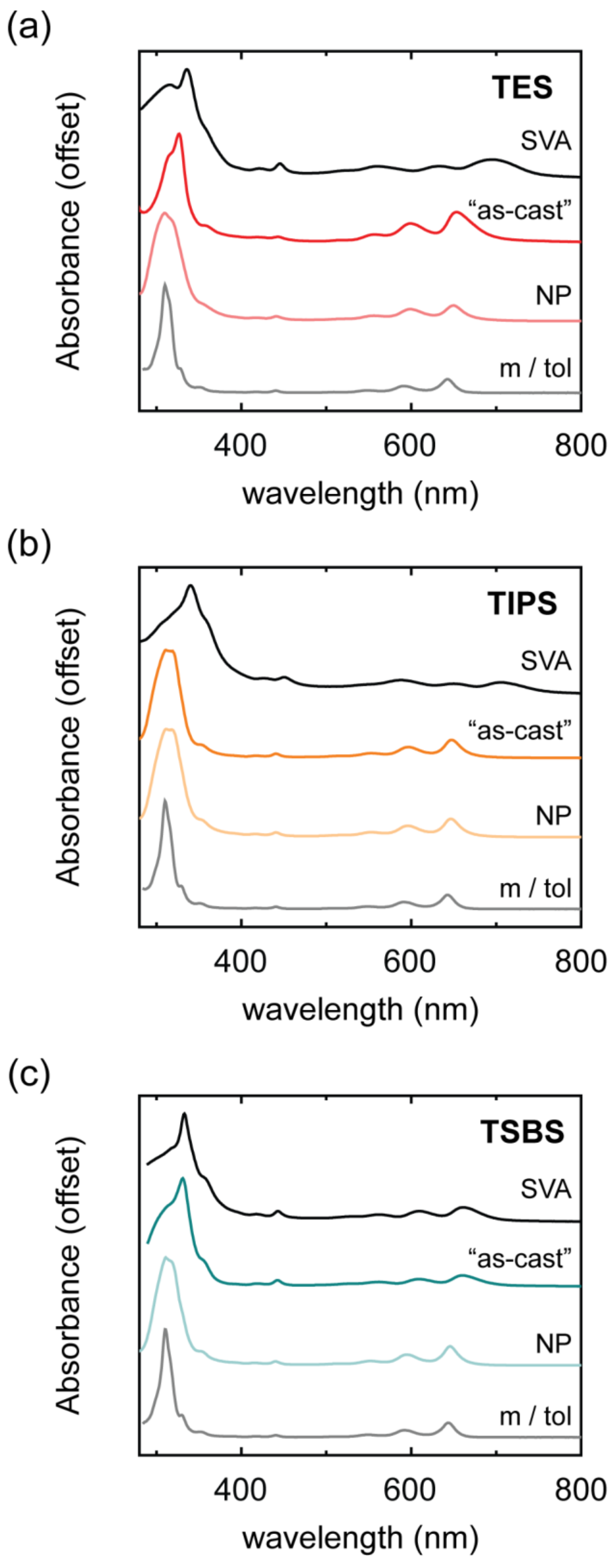

Figure S3.1. Electronic absorption spectra of (a) TES-, (b) TIPS-, and (c) TSBS-pentacene in the form of dilute toluene solutions (grey), aqueous nanoparticle suspensions (light color), “as-cast” films (dark color), and crystalline films (black). For the purpose of best presentation and

comparison, the spectra were normalized to the most intense absorption (appearing in the vicinity of ca. 300-350 nm for all samples), scaled with an equivalent scaling factor, and displaced by a vertical offset.

Figure S3.1 shows that whereas essentially all of the electronic transitions in the absorption spectra of the solvent-vapor annealed films are re-structured as a result of excitonic effects,[3] the corresponding transitions in the absorption spectra of the nanoparticle suspensions more closely resemble that of the respective compound as a dilute toluene solution.

We attempted to compare the absorption spectra of the nanoparticle suspensions directly to that of amorphous material by preparing "as-cast" films of each compound. We first prepared as-cast films of TIPS-Pn where it has been shown that such films can be prepared in an amorphous phase by appropriately tailoring the film deposition conditions,[4] and carefully avoiding crystallization agents.[2] **Figure S3.1b** shows that the absorption spectrum of the TIPS-Pn nanoparticle suspensions nearly identically matches that of the amorphous film over an extended spectral window spanning the ultraviolet through to the near-infrared. The electronic absorption spectrum of TIPS-Pn presented in **Figure S3.1b** encompasses three clearly observable electronic transitions, general to this series of pentacene derivatives: (i) a moderately strong transition appearing in the vicinity of ca. 650 nm, assigned to ^1L_a in Platt's notation, (ii) a relatively weak transition appearing at ca. 450 nm, assigned to ^1L_b in Platt's notation, and (iii) a fully allowed and very strong transition appearing in the vicinity of ca. 300 nm, assigned to ^1B_b in Platt's notation. For a detailed discussion of the electronic transitions of these pentacene derivatives, which are closely analogous to those observed for unsubstituted pentacene, along with a brief discussion of Platt's notation, see e.g. refs. 5 and 6. We previously showed that the electronic absorption spectra of the TIPS-Pn nanoparticle suspensions and amorphous films were essentially indistinguishable in the vicinity of the ^1L_a transition, which strongly suggests the TIPS-Pn nanoparticle suspensions are amorphous (see e.g. ref. 2). We additionally reported time-resolved polarization anisotropy measurements that strongly support this interpretation (see, e.g. ref. 1).

In the present work, we provide additional evidence supporting the interpretation that the TIPS-Pn nanoparticles are amorphous. Specifically, the two strongest electronic transitions in the spectral range in **Figure S3.1b** include the ^1L_a and ^1B_b transitions, which are of moderate and very strong intensity, respectively. These electronic transitions also have transition dipole orientations that are orthogonal. Interestingly, **Figure S3.1b** shows that the stronger electronic transition with an orthogonal transition dipole (i.e., ^1B_b) also does not show signs of strong excitonic effects in the TIPS-Pn nanoparticle suspension; that is, the absorption spectra in the vicinity of the ^1B_b electronic transition in the TIPS-Pn nanoparticle suspensions and amorphous films are essentially equivalent. Thus, we take the fact that both of the strongest electronic transitions of this compound (that also have orthogonal transition dipole moments) are unchanged in the nanoparticle suspensions and amorphous films can be considered as additional evidence supporting our assignment of the solid-state order of TIPS-Pn nanoparticles as amorphous.

We also prepared "as-cast" films of TES- and TSBS-Pn. **Figure S3.2** displays two dimensional grazing-incidence wide-angle X-ray scattering (2D GIWAXS) measurements of the films which indicate that, unlike as-cast TIPS-Pn films that are amorphous,[2,4] both as-cast TES- and TSBS-Pn films are crystalline.

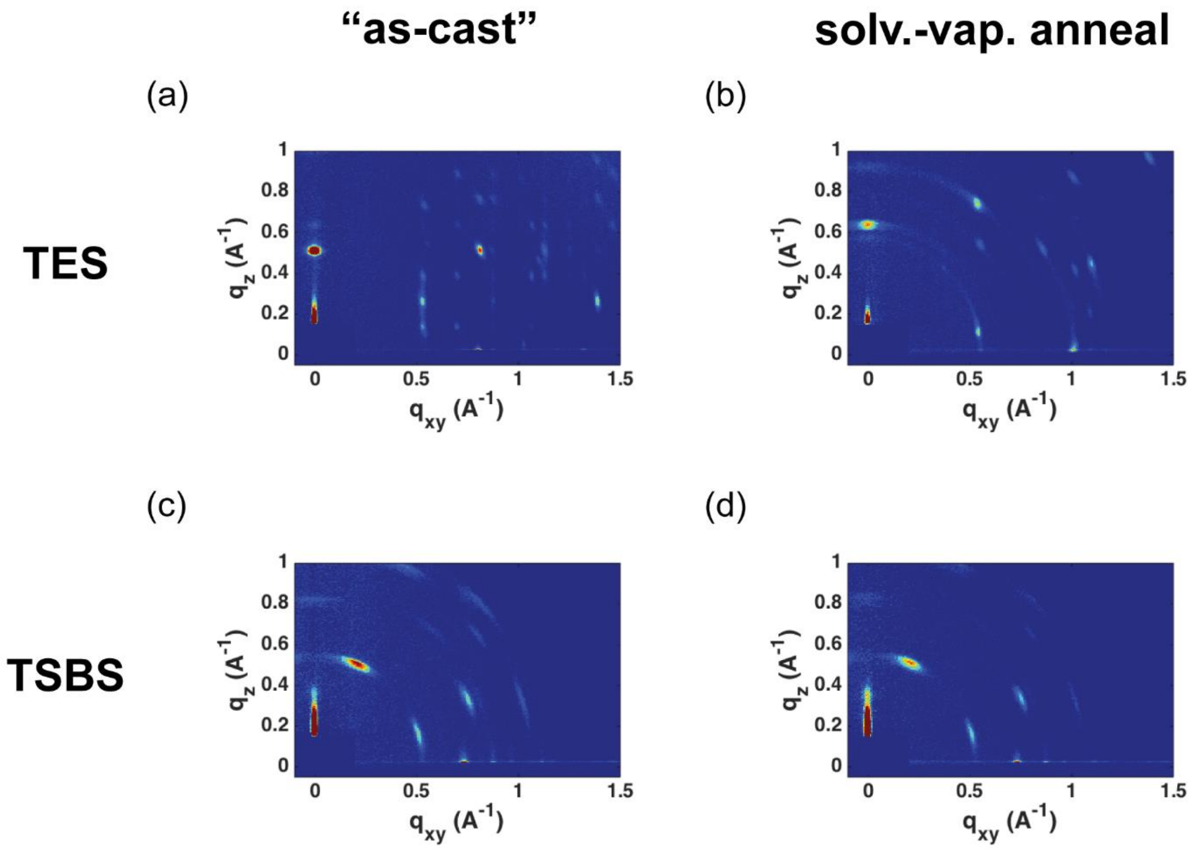

Figure S3.2. Two-dimensional grazing-incidence X-ray diffraction patterns of "as-cast" and solvent-vapor annealed films of (a,b) TES- and (c,d) TSBS-pentacene.

The electronic absorption spectra of the "as-cast" TES- and TSBS-Pn films provide further confirmation of the 2D GIWAXS results. **Figure S3.1c** shows that both ^1L_a and ^1B_b transitions of the "as-cast" TSBS-Pn film are essentially equivalent to that of the solvent-vapor annealed (and crystalline) film. Thus, the electronic absorption spectra further support the 2D GIWAXS measurements; namely, the electronic absorption spectra indicate that the molecules adopt the same molecular-level packing arrangement, which would be anticipated given the equivalent long-range order reported on by the 2D GIWAXS measurements. The 2D GIWAXS measurements indicate that the "as-cast" films of TES-Pn were not amorphous but also showed evidence for crystallinity. In the case of the as-cast TES-Pn films, the 2D GIWAXS measurements (**Figure S3.2a,b**) indicate that two different crystalline forms are adopted, which again is verified at the molecular level through the electronic absorption spectrum. Namely, the electronic absorption spectra of the as-cast and solvent-vapor annealed films of TES-Pn presented in **Figure S3.1b** are different, although each show signs of strong excitonic effects. This is most obvious by considering the ^1L_a transition in the crystalline films, which is redshifted appreciably and exhibits a different absorption strength as compared with both the ^1B_b and ^1L_b transitions. Furthermore, and perhaps most critically, there are clear signatures of strong excitonic effects in the fully allowed ^1B_b transition. Namely, the ^1B_b transition exhibits a considerable redshift and change in transition

intensity in both the as-cast and solvent-vapor annealed films. In fact, the considerable redshift observed in the as-cast film trends towards that of the solvent-vapor annealed film. Thus, the strong excitonic effects observed on the $^{1}L_{a}$ and $^{1}B_{b}$ transitions in the TES-Pn films indicates that their crystalline nature correspond to regular changes in the local molecular-level packing that sensitively impact their electronic absorption spectra. In summary, both the 2D GIWAXS measurements and electronic absorption spectra indicate that as-cast TES- and TSBS-Pn films are crystalline; thus, a direct comparison between the nanoparticle suspensions and amorphous films of these pentacene derivatives is not possible.

In lieu of a direct comparison of the absorption spectrum of the pentacene derivative nanoparticles with that of the respective amorphous films, we can still compare the absorption spectra of the pentacene derivative nanoparticles with that of their respective crystalline films along with that of the amorphous films of TIPS-Pn. Considering that the absorption spectrum of the TSBS-Pn nanoparticle suspension does not very well match that of either the "as-cast" or solvent-vapor annealed film, but much more strongly resembles that of the amorphous TIPS-Pn nanoparticles and films, we assign the solid-state order in the TSBS-Pn nanoparticles as amorphous.

Lastly, we consider the solid-state order of the TES-Pn nanoparticle suspensions. Considering that the crystalline nature of the "as-cast" and solvent-vapor annealed TES-Pn films has a strong influence on their absorption spectra, in order to assign the solid-state order of the TES-Pn nanoparticle suspensions we compare their electronic absorption spectra to that of: (i) the crystalline TES-Pn films, (ii) the dilute TES-Pn in toluene solution, and (iii) the amorphous TIPS-Pn films. **Figure S3.1a** shows that the electronic absorption spectra of the TES-Pn nanoparticle suspensions does not compare well with that of the crystalline films. In fact, the absorption spectrum of the TES-Pn nanoparticle suspensions much more strongly resembles that of the isolated chromophore in dilute toluene solution, clearly indicating that the molecules are not strongly excitonically coupled. While not a direct confirmation of solid-state order, a comparison of the electronic absorption spectrum of the TES-Pn nanoparticle suspensions with that of the amorphous TIPS-Pn films is insightful. Given that the absorption spectrum of the TES-Pn nanoparticle suspensions very closely resembles that of the amorphous TIPS-Pn films (and amorphous TIPS- and TSBS-Pn nanoparticles), we assign the solid-state order of the TES-Pn nanoparticle suspensions as amorphous.

<u>Section S4:</u> Variations in excitonic coupling and mass density explain trends in steady-state absorption spectra

We quantitatively examined the absorption spectra of the amorphous pentacene derivative nanoparticles while considering the molecules different propensities to π-stack. Consistent with the expectation that the molecules would exhibit different propensities to π-stack in the amorphous nanoparticles, we observe considerably different intensities in the vibronic progressions in the absorption spectra of these different pentacene derivative nanoparticles (**Fig. 2a**). This is especially clear when considering that TES-, TIPS-, and TSBS-Pn have essentially the same absorption spectrum when dissolved in a "good" organic solvent, such as toluene (**Figure S4**).

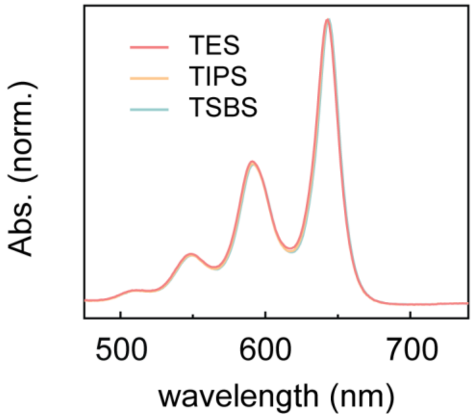

Figure S4. Steady-state absorption spectra of dilute solutions of TES-, TIPS-, and TSBS-pentacene in toluene.

Comparing the nanoparticle spectra to the corresponding toluene solution spectra, we find that the ratio of the amplitude of the 0-0 and 0-1 vibronic bands decreases (or the 0-1 vibronic band gets larger for a constant 0-0 vibronic band) successively in the series TSBS-, TIPS-, and TES-Pn (**Table S4**). These results indicate that as side chains become more compact: (i) the molecules comprising the nanoparticles tend to exhibit H-type aggregation or face-to-face π-stacking,[7–9] and (ii) the average effective excitonic coupling becomes progressively stronger.[7,10] The progressively reduced peak extinction of the origin band of the transition of nanoparticles in the series TES-Pn < TIPS-Pn < TSBS-Pn (**Fig. 2a**) further supports (ii). These observations are consistent with the expectation that chromophores in the amorphous TES-Pn nanoparticles have enhanced π-stacking while chromophores in the amorphous TSBS-Pn nanoparticles have inhibited π-stacking.

Table S4. Ratio of the 0-0 and 0-1 Vibronic Bands and Shift of the Lowest-Energy Singlet Transition

**Compound**	**$A_{0\text{-}0}/A_{0\text{-}1}$**		**Ratio (%)**	**$E_{singlet}$ (cm^{-1})**		**$\Delta E_{singlet}$ (cm^{-1})**
	**monomer**	**nanoparticle**		**monomer**	**nanoparticle**	
TSBS-Pn	2.0	1.8	110	15,520	15,470	50
TIPS-Pn	2.0	1.6	130	15,540	15,440	100
TES-Pn	2.0	1.3	150	15,540	15,370	170

In addition to systematic variations in the vibronic band ratios, we also observe a successively redshifted lowest-energy singlet exciton transition energy (**Fig. 2b**). Specifically, we observe a lowest-energy singlet exciton transition energy that is redshifted compared with the respective compound dissolved in toluene by 50, 100, and 170 cm^{-1} for TSBS-, TIPS-, and TES-Pn nanoparticles, respectively (**Table S4**). A redshift from the gas phase to the solid state arises from changes in the polarization energy due to van der Waals interactions in the solid state.[11] Thus, the redshift that we observe indicates that the van der Waals interactions and polarization energies are both changing as side chain size decreases, likely because smaller side chains are able to form more compact solids with a higher mass density.[12,13] We can therefore infer from the redshifted singlet exciton energy that the nanoparticles of TSBS-Pn are a solid material where the molecules pack in the least compact manner with the lowest mass density, whereas nanoparticles of TES-Pn are a solid where the molecules pack in the most compact manner with the highest mass density.

In summary, the observations reported above are consistent with the expectation that chromophores in the amorphous TES-Pn nanoparticles have enhanced π-stacking and pack in a more compact manner while chromophores in the amorphous TSBS-Pn nanoparticles have inhibited π-stacking and pack in a less compact manner.

<u>Section S5:</u> Isolated-chromophore singlet excitation and parent singlet exciton transient absorption spectra compare well

We assign the photoinduced absorption feature appearing at ca. 1400 nm in the early-time transient near-infrared spectra of the nanoparticles to parent singlet excitons because this feature very well matches that of singlet excitations generated on isolated molecules in dilute toluene solution (**Figure S5**). In solutions of TIPS-pentacene, following light absorption and on the sub-nanosecond timescale primarily singlet excitations are populated.[14] Because the alkyl substituent of the solubilizing group has a negligible influence on the electronic structure of these chromophores (**Figure S3.1**), we presume that primarily singlet excitations are populated in the case of TES- and TSBS-pentacene in toluene, as well. Fluorescence lifetime measurements reported in **Section S8** represent additional evidence supporting this interpretation. For all compounds, the dilute toluene solution and nanoparticle transient absorption spectra exhibit excellent agreement and are nearly indistinguishable. The only exception is the comparison between TES-pentacene in toluene and the amorphous TES-pentacene nanoparticle suspensions; while the overall spectral profiles are in excellent agreement, the relative peak amplitudes are slightly different. This likely results from the stronger average effective excitonic coupling of the chromophores observed in this sample (**Fig. 2a** and **Section S4**). It should be noted that the transient spectra of the nanoparticle suspensions are limited to wavelengths less than 1400 nm due to strong water absorption above this wavelength.

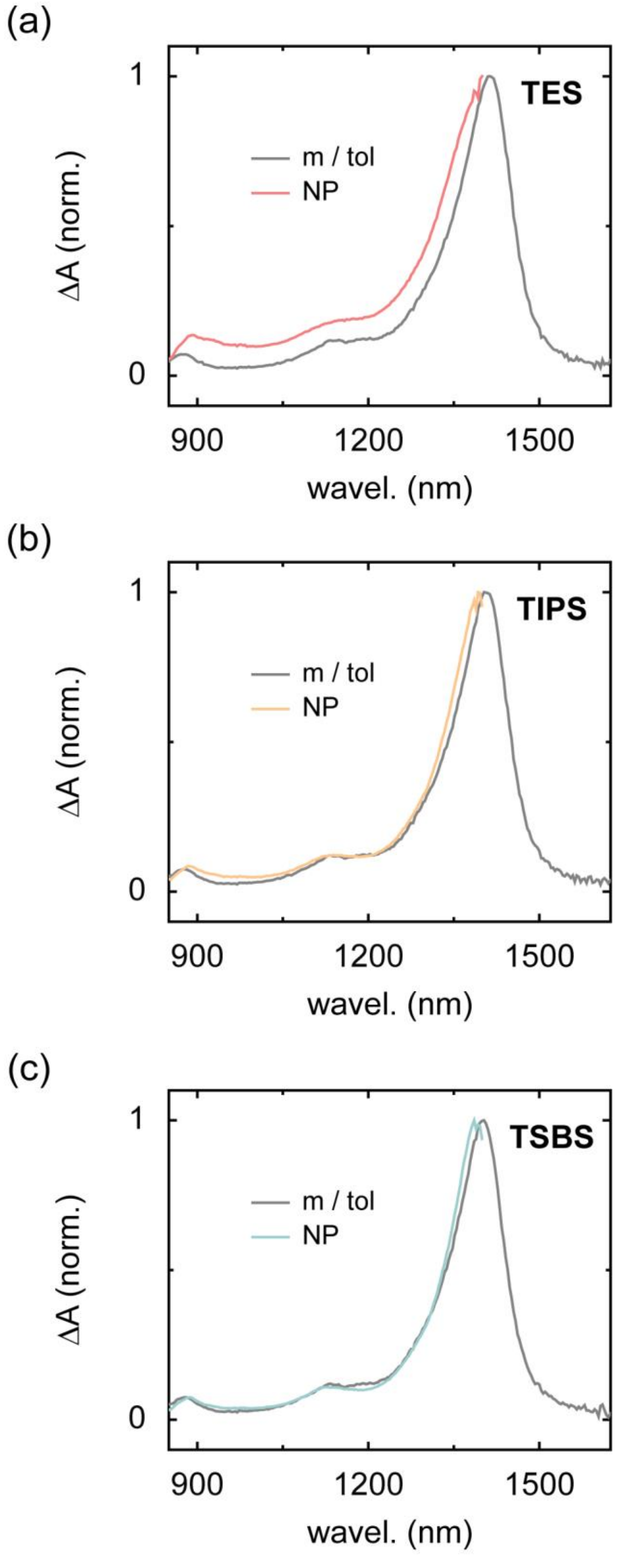

Figure S5. Time-averaged (0 – 150 fs) transient near-infrared absorption spectra of dilute toluene solutions and amorphous nanoparticle suspensions of (a) TES-, (b) TIPS-, and (c) TSBS-Pn. The

measurements were performed with an incident pump wavelength and fluence of ca. 646 nm and 260 μJ/cm^2, respectively.

<u>Section S6:</u> Isolated-chromophore triplet excitation and nanoparticle long-time transient absorption spectra compare well

We assign the photoinduced absorption feature appearing in the transient near-infrared spectra in the nanoparticles at long-time delay to a triplet excited-state absorption because the spectra very well match that of the isolated molecule in dilute toluene solution measured on the several hundred nanosecond timescale (**Figure S6**). On the several hundred nanosecond timescale, singlet excitations on isolated TIPS-pentacene molecules convert into triplet excitations through intersystem crossing.[14] Because the alkyl substituent of the solubilizing group has a negligible influence on the electronic structure of these chromophores (**Sections S4, S5, and S8**), we anticipated and presume that intersystem crossing is the decay pathway responsible for generating triplet excitations in the case of dilute toluene solutions of TES- and TSBS-pentacene, as well.

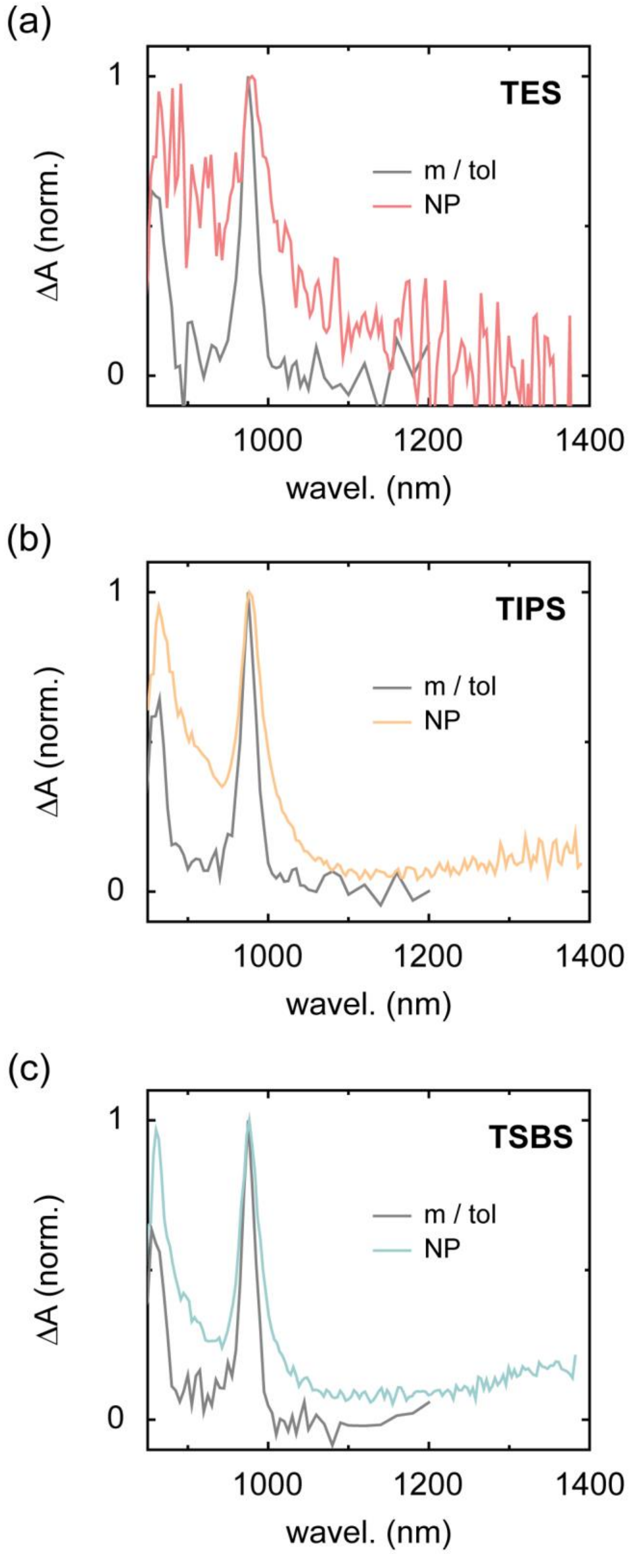

Figure S6. Time-averaged (100 – 500 ns) transient near-infrared absorption spectra of dilute solutions of TES-, TIPS-, and TSBS-pentacene in toluene compared with time-averaged (0.8 – 1.3 ns) transient near-infrared absorption spectra obtained from aqueous TES-, TIPS-, and TSBS-

pentacene nanoparticle suspensions. The dilute toluene solution and aqueous nanoparticle suspension measurements were performed with an incident pump fluence of ca. 100 μJ/cm^2 and 260 μJ/cm^2, respectively.

It is interesting to note that the nanoparticle spectra exhibit slight differences in peak width and vibronic peak ratio, likely from solid-state effects similar to those discussed in detail in **Sections S4 and S17**.

<u>Section S7:</u> Qualitative arguments can be made that the electronic couplings relevant to triplet pair formation are stronger for the H-aggregated chromophores even after accounting for the concentration of and singlet diffusion to fission sites

In prior studies of TT formation in amorphous solids,[1,15,16] the authors observed non-monoexponential TT formation and attributed these complex dynamics to a combination of factors, including both the concentration of and singlet diffusion to SF sites. Here, we challenge several assumptions inherent to these models in the hopes of providing additional insights into the nature of complex, non-monoexponential TT formation dynamics in the amorphous state.

The nature and concentration of SF sites in amorphous material

We first address the nature of the SF sites, which is relevant to the discussion of their concentration. It is often assumed that the pair of molecules at the SF site adopt the molecular packing of a crystalline polymorph of the material that has a strong propensity to form TT. Crystalline material generally (although not always) exhibits strong excitonic peak shifts in their electronic absorption spectra, such that they may possibly be identified using UV/Vis absorption spectroscopy. Given the modest detection sensitivity of UV/Vis absorption spectroscopy (i.e., pph-ppt), however, one could argue that low concentrations of these SF sites may be present and responsible for the majority of the singlet fission dynamics that UV/Vis absorption spectroscopy is unable to detect. Transient absorption spectroscopy, another technique often employed in SF studies, has appreciably higher detection sensitivity of the order of ca. 10^{-5}. We argue, without going into great detail, that this level of detection sensitivity is sufficient to identify and characterize the nature of SF sites in both crystalline *and* amorphous material. Key to the following discussion is that if the SF sites comprise molecules that have adopted the molecular packing of a crystalline polymorph of the material, then TT photogenerated at these SF sites should look like triplets photogenerated in the crystalline material.

Figure S7.1 displays the transient absorption spectra of separated triplet pairs in crystalline material of the TAS-Pn, which are representative of the transient absorption spectra of isolated triplets in crystalline material,[17] along with the transient absorption spectra of nascent triplet pairs in amorphous material of the TAS-Pn, representative of the triplet pairs formed immediately following the decay of primary singlet excitons at the dimer pair sites.

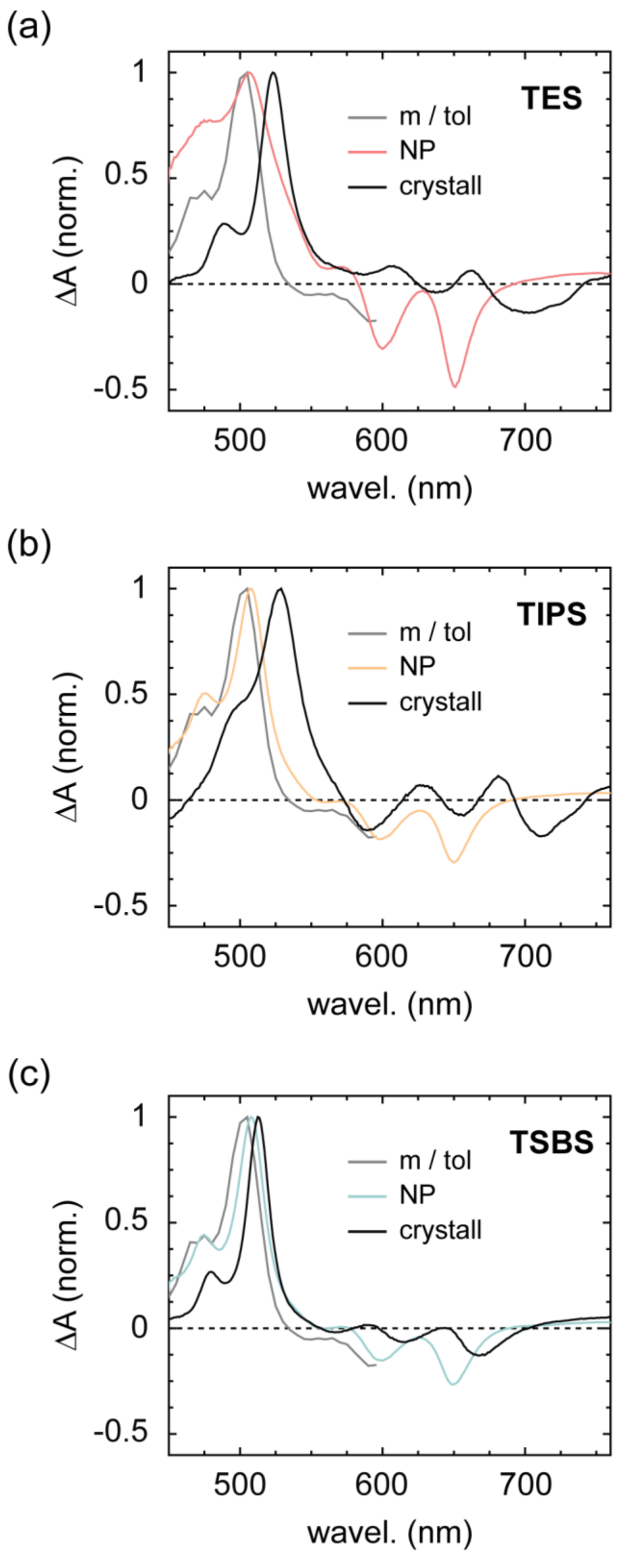

Figure S7.1. Transient absorption spectra of triplet pairs photogenerated in the amorphous pentacene derivative nanoparticles and of triplets photogenerated in crystalline material. The transient absorption spectra for the triplets and triplet pairs in crystalline films and amorphous

pentacene derivative nanoparticles, respectively, were each obtained a time delay of ca. 30 ps. Overlaid in each plot is the transient absorption spectrum of isolated-molecule triplets photogenerated on TIPS-Pn in toluene, which were measured at a time delay of several hundred nanoseconds.

Clearly, the transient absorption spectra of triplet pairs photogenerated in the amorphous material do not match the transient absorption spectra of triplets photogenerated in crystalline material. Rather, they more closely resemble the transient absorption spectra of isolated-molecule triplets photogenerated on TIPS-Pn (which, given the results above in **Sections S4-S6**, we take to be representative of isolated-molecule triplets photogenerated on TES- and TSBS-Pn). Thus, although SF is fast in the material in its crystalline form, we can conclude through the transient absorption spectroscopy that the SF sites in the amorphous pentacene derivative solids is *not* that of the lowest-energy, equilibrium crystalline molecular packing configuration.

It is possible that other crystalline polymorphs, that are not the lowest energy, could be acting as the SF sites in the amorphous material. For example, Dron *et al.* hypothesized that the SF sites in the amorphous state of various diphenylisobenzofuran derivatives might be represented by the α-1 polymorph of the compound, which is not the lowest-energy crystalline state.[16] We suggest that, in a similar logic as above, that a comparison should be made between isolated-molecule triplets photogenerated on this material and the transient absorption spectrum of the triplet pair photogenerated in the amorphous material, and the triplet transient absorption spectrum of the triplets photogenerated on the isolated-chromophore in toluene, α-1 polymorph. If these latter two spectra are to match, this is a direct confirmation that the SF sites are represented by this kinetically-trapped polymorph of DPIBF. In contrast, if the two spectra do not match, this disproves this *particular* polymorph of DPIBF as being representative of the SF site. However, it does not disprove the possibility of another polymorph in which the molecules are weakly electronically coupled.

Another possibility, which we previously suggested,[1] is that a distribution of molecular packing arrangements that gives rise to a distribution of site energies and couplings produces a distribution of TT formation rates. At the most general level, we can express the observed TT formation rate as a sum or integral of all of the rates of the individual constituents, i.e.:

$$A_1: \quad S_1 + S_0 \xrightarrow{k_{SF,1}} T_1 + T_1$$

$$A_2: \quad S_1 + S_0 \xrightarrow{k_{SF,2}} T_1 + T_1$$

$$A_3: \quad S_1 + S_0 \xrightarrow{k_{SF,3}} T_1 + T_1$$

$$\vdots$$

$$A_n: \quad S_1 + S_0 \xrightarrow{k_{SF,n}} T_1 + T_1$$

$$k_{obs} = \sum_n A_n k_{SF,n}$$

This formulation covers a distribution of any arbitrary complexity. For example, a completely random distribution of molecular packing arrangements would give rise to a continuous distribution of TT formation rate constants. One the other hand, if there are preferred geometries, the distribution would be biased in a manner determined by these preferred geometries. If there is a more complex, *bimodal* distribution of preferred geometries, then the observed TT formation rate could be accurately modeled as the weighted-average of the rate constants of the distribution as described above. Thus, it is not essential to assume that amorphous material is a completely random arrangement of chromophores to give rise to a complex, non-exponential TT formation rate.

Elucidating the nature of the SF sites and the nature of their distribution is relevant to a discussion of their concentration. If indeed the SF sites are some complex, but not completely random, distribution of molecular packing arrangements that are not equivalent to the equilibrium crystalline packing arrangement, we can readily visualize many different conformations suitable for singlet fission (several studies have highlighted how robust pentacene derivatives are at singlet fission[1,18]). Within this hypothetical medium, we can also visualize a high density of SF sites. Indeed, in support of this assertion Roberts *et al.* suggested an appreciable concentration of SF sites in amorphous films of the singlet fission chromophore 5,12-diphenyltetracene.[15] It would be ideal to characterize the short-range order inherent to the material. For example, what molecules packing arrangements are suitable for SF, and how many such packing arrangements are there? As noted by Dron *et al.*, however, this type of short-range order is exceedingly difficult if not impossible with to characterize with conventional characterization methods.[16] We therefore defer further discussion of this matter, and assume that the density of SF sites in the amorphous state is sufficiently high so as not to obscure an interpretation of the observed TT formation time directly in terms of the intrinsic SF rate.

In summary, we conclude that, at least for the amorphous pentacene derivative solids studied in this work, the SF sites do not adopt the molecular packing of the lowest-energy crystalline form, that the most likely explanation for the nature of the singlet fission sites is some complex distribution of molecular-level packing arrangements (where, in the main text, we shed further insights into that distribution), and that the density is sufficiently high so as not to obscure an interpretation of the TT formation time in terms of the intrinsic singlet fission dynamics.

Singlet diffusion and TT formation

We next discuss how singlet diffusion impacts an interpretation of the observed TT formation time in terms of the intrinsic SF rate.

We first highlight that one should again keep in mind the timescales of TT formation for a material when considering this matter. In brief, the influence of singlet diffusion will be particularly problematic for materials showing slow TT formation timescales, such as in crystalline tetracene. In a pioneering contribution, Piland *et al.* showed that energy migration within polycrystalline tetracene caused the timescale of TT formation to be faster because singlet diffusion occurred to grain boundaries where the authors suggested TT formation was faster.[19] Thus, the slower that TT formation is the more sensitive the observed TT formation time will be to non-intrinsic phenomena such as singlet diffusion and sample heterogeneity. As we highlighted previously,[17] pentacene derivatives are especially advantageous in the context given the exceptionally rapid picosecond and sub-picosecond TT formation timescales.

With regards to the pentacene derivatives of the present work, TT formation is quite fast. We previously showed that TT formation generally occurs on a timescale of ca. 1 ps for a variety of derivatives in the amorphous state and on a timescale of ca. 100 fs in various materials in their crystalline form.[1,2,17] In the case of crystalline material, competition between singlet diffusion and TT formation will certainly be highly competitive, and it is not too illogical to assume that primary singlet excitons will not diffuse too far before they fission into triplet pairs. Indeed, Pensack *et al.* noted an exceptional lack of singlet-singlet annihilation as a function of incident pump fluence in this material.[2] The question remains, though, does the same argument hold for the amorphous state? And if singlet diffusion does occur, how substantially does it complicate an interpretation of the observed TT formation directly in terms of the intrinsic SF rate? Before proceeding to attempt to address these questions, we note that Pensack *et al.* also tested the fluence-dependence of the transient absorption signal of one of these TAS-Pn derivatives, namely, TIPS-Pn and observed *negligible singlet-singlet annihilation*;[2] thus, even in the amorphous state primary singlet excitons do not diffuse very far before fissioning into triplet pairs.

As an additional means in which to address whether or not diffusion of parent singlet excitons complicates a direct interpretation of the TT formation time in terms of the intrinsic SF dynamics, we performed pump-probe anisotropy measurements on the amorphous nanoparticles (**Figure S7.2**). While preclude a complete analysis of these results from this work, insights can still be gained from these data analyzed in a semi-quantitative manner. Specifically, determining the singlet diffusion (or energy migration) time constant according to the first two time constants of a tri-exponential fit to these data, we find evidence that the timescale of singlet diffusion is

either faster than or equivalent to the timescale of TT formation in the amorphous pentacene derivative nanoparticles (**Table S7**).

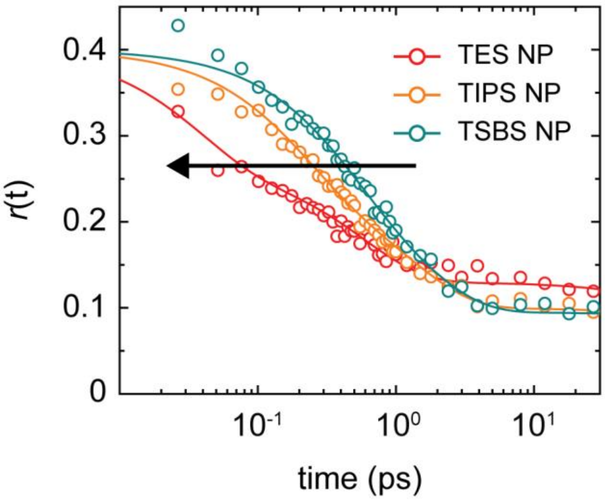

Figure S7.2. Pump-probe anisotropy measurements of amorphous TES-, TIPS-, and TSBS-pentacene nanoparticles. Overlaying the data are tri-exponential fit functions, the first two components of which we assign to early-time (picosecond/sub-picosecond) singlet diffusion (or more formally, energy migration). The time constants reported in **Table S7** reflect the weighted average of these two initial time constants.

Table S7. Time Constants for Triplet Pair Formation and Singlet Diffusion in Amorphous Pentacene Derivative Nanoparticles [a,b]

Compound	τ_{TPF} (ps)	τ_{SD} (ps)
TES-Pn	0.42 ± 0.03	0.3
TIPS-Pn	1.23 ± 0.06	0.8
TSBS-Pn	2.8 ± 0.5	1

[a] Time constants for triplet pair formation were taken from **Section S9**, which reports the same values in terms of the parent singlet exciton lifetime.
[b] Time constants associated with singlet diffusion, or more formally the energy migration of primary singlet excitons, were determined by taking the weighted-average of a bi-exponential fit to the early-time anisotropy decay.

Although singlet diffusion is indeed not *entirely* separable from the intrinsic SF dynamics in these materials, qualitative arguments can still be made. If the rate of singlet diffusion is essentially as fast as the rate of TT formation, this means that the rate of TT formation is either equivalent to or faster than the rate of singlet diffusion. That is, singlet diffusion to SF sites can be considered rate-determining step and the rate of TT formation can be considered as fast or faster than that of singlet diffusion. We observe this scenario for the amorphous TES-Pn nanoparticles, indicating that the observed timescale for TT formation should be taken as an upper bound to the intrinsic timescale of TT formation for the material which comprises primarily H-aggregated dimer

pair sites. In the case of the amorphous TSBS-Pn nanoparticles, on the other hand, the rate of singlet diffusion is appreciably faster than the rate of TT formation. Specifically, we determined a time constant of ca. 1 ps for singlet diffusion and ca. 2.8 ps for triplet pair formation (**Table S7**). Thus, TT formation is considerably slower than singlet diffusion in this system. This means that singlet diffusion is not rate determining the case of the kinetics of this sample. We thus take these results to indicate that the intrinsic rate of TT formation associated with the distribution of largely monomer-like dimer pair sites in the amorphous TSBS-Pn nanoparticles to be larger than the rate of singlet diffusion between the chromophores comprising the distribution.

Concluding this section, we showed that the rate of TT formation in the amorphous TES-Pn nanoparticles, with a large population fraction of H-aggregate dimer pair sites, is the same or faster than the rate of singlet diffusion (which is ca. 0.3 ps) meaning that singlet diffusion is rate determining and that the rate of TT formation should be considered as fast or faster than that of singlet diffusion (i.e., ca. 0.4 ps or faster) while the rate of TT formation in the amorphous TSBS-Pn nanoparticles, with a large fraction of monomer-like dimer pair sites, which is lower than the rate of singlet diffusion (which is ca. 1 ps) to be representative of the intrinsic SF dynamics. These qualitative insights are consistent with our assertion that the electronic matrix element(s) coupling S_1S_0 and TT for the H-aggregate and monomer-like dimer pair sites is (are) stronger and weaker, respectively.

<u>Section S8:</u> Fluorescence lifetimes of dilute solutions of TES-, TIPS-, and TSBS-pentacene in toluene

All of the compounds, as dilute solutions in toluene, exhibit essentially indistinguishable emission spectra (**Figure S8.1**), with a vibronic origin band peaking at ca. 647 nm.

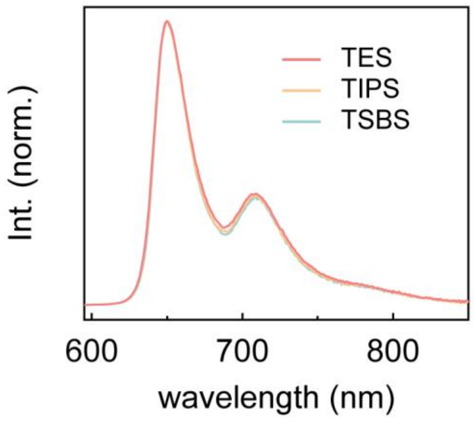

Figure S8.1. Fluorescence spectra of dilute solutions of TES-, TIPS-, and TSBS-pentacene in toluene.

The fluorescence decays measured for the corresponding samples, exciting at 507 nm and detecting at 650 nm, are displayed in **Figure S8.2**. All of the compounds exhibit a fluorescence lifetime of ca. 12 ns.

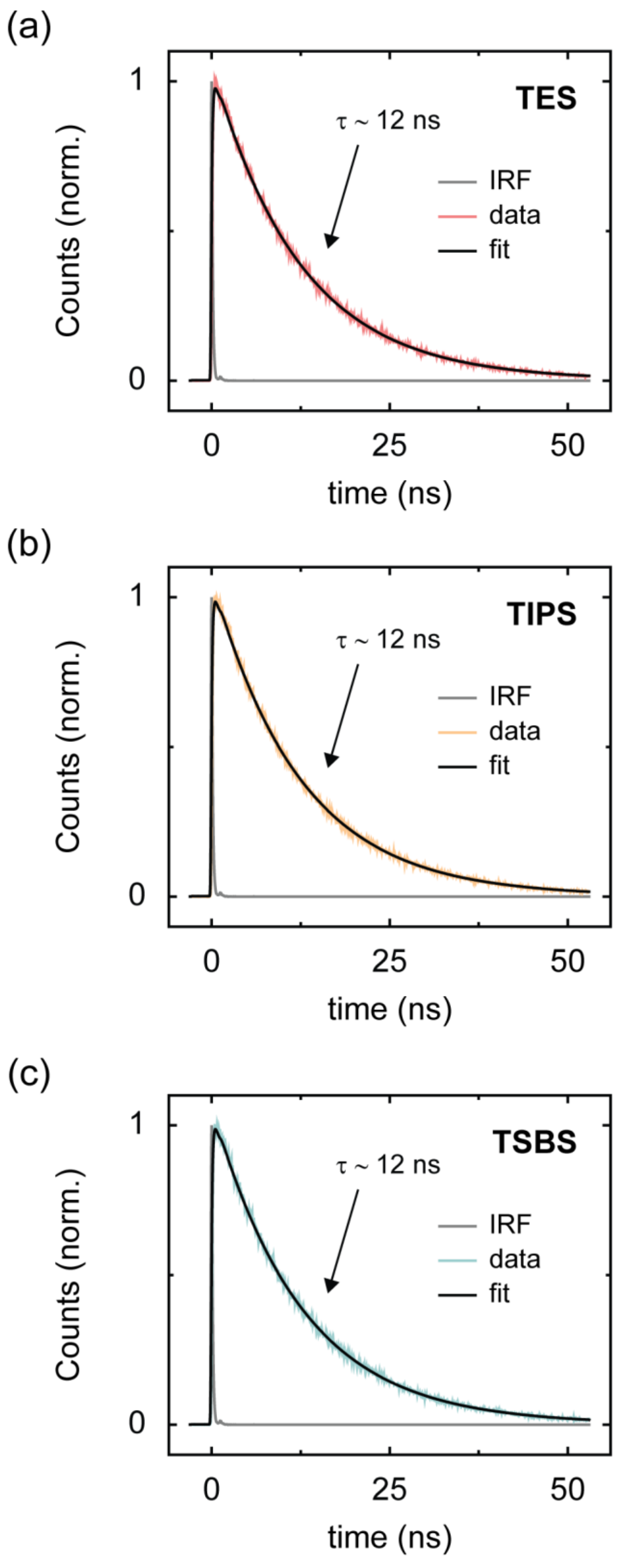

Figure S8.2. TCSPC of TES-, TIPS-, and TSBS-pentacene in toluene. Overlaying the fluorescence decays are best fits determined via convolution with the instrument response function.

<u>Section S9:</u> Parent singlet exciton lifetimes in amorphous TES-, TIPS-, and TSBS-pentacene nanoparticles

In this supporting section, we report on how we derived the parent singlet exciton lifetimes in the amorphous TES-, TIPS-, and TSBS-Pn nanoparticle suspensions. We showed in **Section S5** that a prominent photoinduced absorption band in the near-infrared peaking at ca. 1400 nm can be assigned to parent singlet excitons. In order to determine the parent singlet exciton lifetime in the amorphous TES-, TIPS-, and TSBS-Pn nanoparticle suspensions, we averaged the near-infrared singlet photoinduced absorption band over a spectral region where the triplet photoinduced absorption band, reported in **Section S6**, does not overlap; specifically, we averaged the data over a spectral range from 1100 – 1390 nm. The resultant transient kinetic traces are displayed in **Figure S9**.

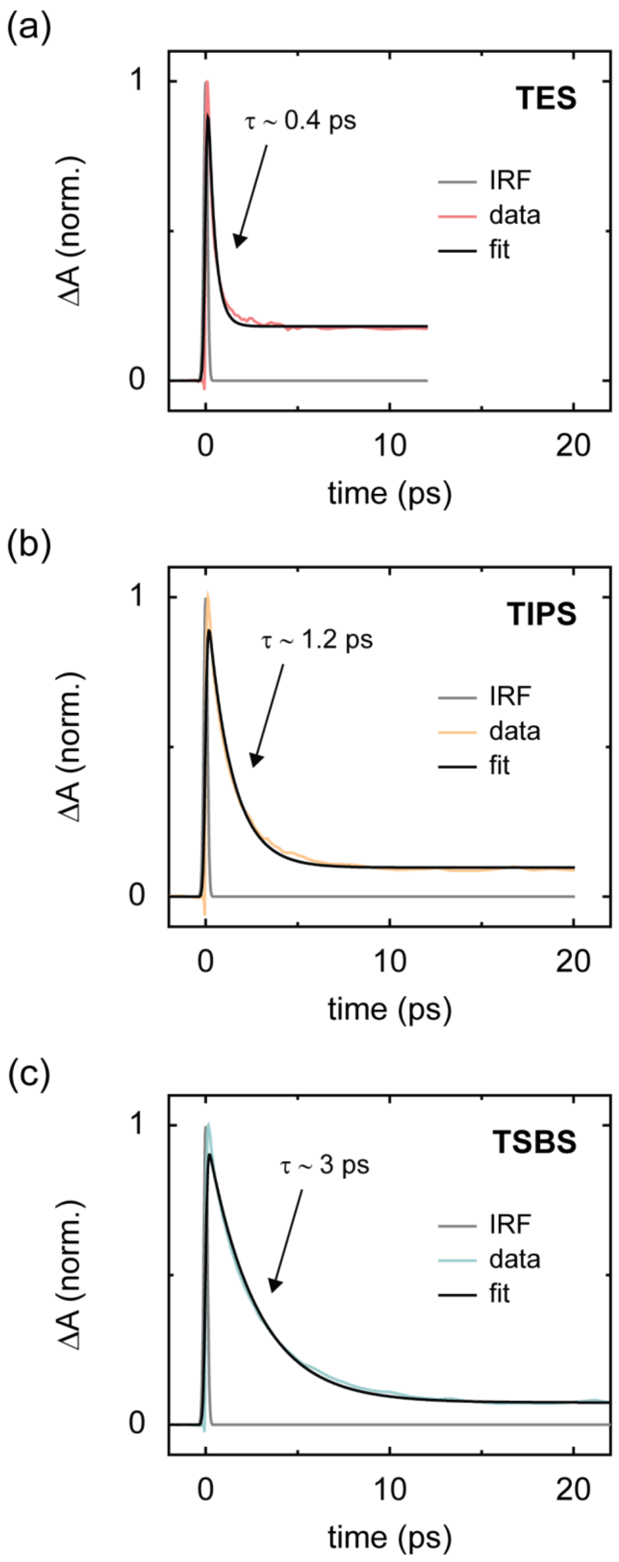

Figure S9. Representative traces for the decay of the near-infrared photoinduced absorption band associated with parent singlet excitons in the amorphous TES-, TIPS-, and TSBS-Pn nanoparticles, including the fits and instrument response function used to model the data. The near-infrared

photoinduced absorption band data were obtained by averaging over the spectral range from 1100 – 1390 nm. Based on the ca. 100 fs time constant we measured previously for the pump pulse duration for the transient absorption spectrometer (see ref. 2), a Gaussian function with a time constant of 100 fs was used as the instrument response function.

Based on the analysis of the decay of the near-infrared photoinduced absorption band associated with parent singlet excitons, we determine lifetimes of 0.42 ± 0.03 ps, 1.23 ± 0.06 ps, and 2.8 ± 0.5 ps for parent singlet excitons in the amorphous TES-, TIPS-, and TSBS-Pn nanoparticles, respectively (**Table S9**). Error limits were determined by performing the analysis on three independently prepared and measured samples.

Table S9. Lifetimes Determined for Parent Singlet Excitons in the Amorphous Pentacene Derivative Nanoparticles [a]

Compound	τ_{S1S0} (ps)
TES-Pn	0.42 ± 0.03
TIPS-Pn	1.23 ± 0.06
TSBS-Pn	2.8 ± 0.5

[a] The parent singlet exciton (S1S0) lifetime was obtained by averaging over at least three independent measurements (and sample preparations). The limits represent an analysis of a single standard deviation of the time constants obtained from the fits.

In the main text, we take the time constant associated with the decay of parent singlet excitons to be equivalent to the time constant associated with triplet pair formation, i.e., $\tau_{TPF} = \tau_{S1S0}$.

Section S10: **Triplet lifetimes obtained for isolated chromophores in dilute toluene solutions of TES-, TIPS-, and TSBS-pentacene**

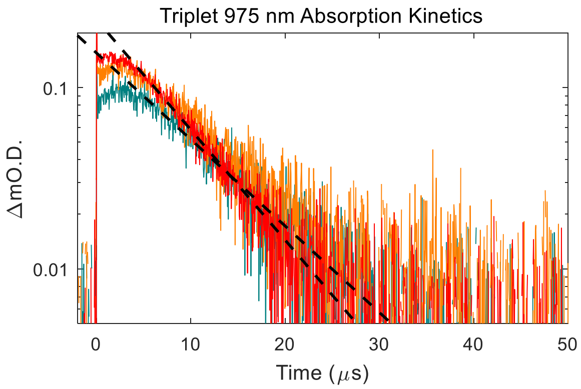

Figure S10. Transient kinetics of triplet excitations in dilute (2×10^{-5} M) toluene solutions of TES-, TIPS-, and TSBS-pentacene. The transient kinetics were obtained at the origin band of the near-infrared transition at ca. 975 nm. The data are plotted in a semilogarithmic fashion. Overlaying the data are dashed lines that correspond to monoexponential fits.

Table S10. Isolated-Chromophore Triplet Lifetimes for Trialkylsilyl-Substituted Pentacene Derivatives [a]

Compound	$\tau_{triplet}$ (μs)
TES-Pn	7.2 ± 0.1
TIPS-Pn	9.7 ± 0.1
TSBS-Pn	9.6 ± 0.2

[a] Errors values were determined from the exponential fitting procedure, and were taken as two standard deviations.

<u>Section S11:</u> Transient visible absorption of TES-, TIPS-, and TSBS-pentacene nanoparticles

Figure S11 displays representative transient visible absorption of the TES-, TIPS-, and TSBS-pentacene nanoparticles (from which the ground-state bleach kinetics in the main text were derived).

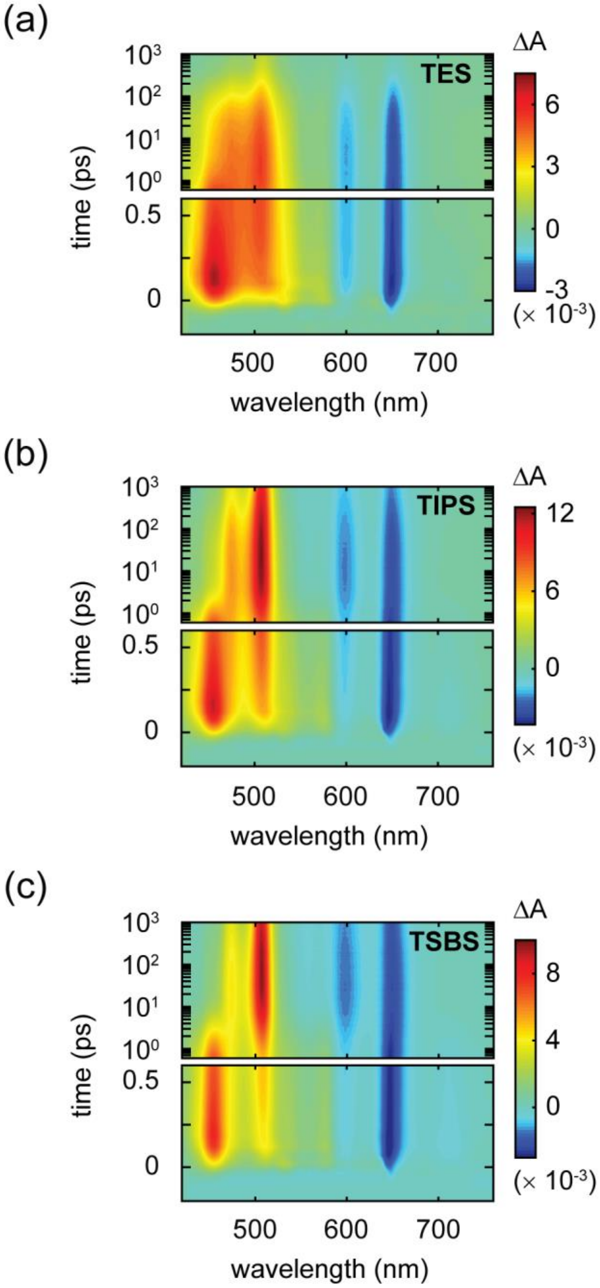

Figure S11. (a-c) Transient visible absorption of the TES-, TIPS-, and TSBS-pentacene nanoparticles. These representative measurements were performed with an incident pump fluence of 200 μJ/cm^2.

<u>Section S12:</u> Transient absorption of the pentacene derivative nanoparticles is not influenced by the presence of molecular oxygen

We performed measurements on the pentacene derivative nanoparticle suspensions both in the presence and absence of molecular oxygen. Molecular oxygen was forced out of the aqueous nanoparticle suspensions by bubbling nitrogen gas through the aqueous nanoparticle suspensions for an extended period of time (i.e., ca. 30 min to 1 hr). We observed indistinguishable transient absorption in the case of both measurements. **Figure S12** displays the transient absorption kinetics of the TES-, TIPS-, and TSBS-Pn nanoparticles averaging over wavelengths associated with overlapping ground-state bleach and stimulated emission features. The representation of the transient kinetics is analogous to that displayed in **Fig. 4a** in the main text. Clearly, the presence of molecular oxygen has no effect on the observed triplet pair losses.

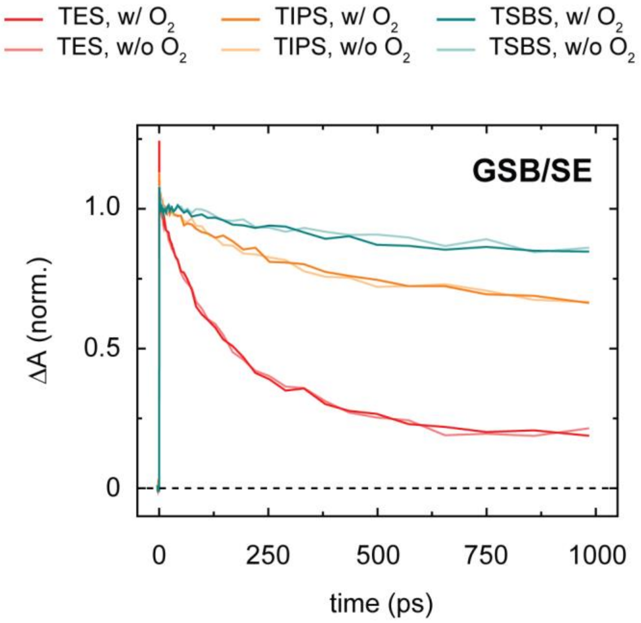

Figure S12. Transient absorption kinetics of TES-, TIPS-, and TSBS-pentacene nanoparticles in the visible spectral region. The transient absorption kinetics were obtained by averaging over wavelengths associated with overlapping ground-state bleach and stimulated emission features. Specifically, the data were averaged over wavelengths ranging from 630 to 690 nm. The data were normalized to the maximum signal amplitude in the vicinity of ca. 3-5, 5-15, and 10-30 ps for the TES-, TIPS-, and TSBS-pentacene nanoparticle suspensions, respectively. The colors associated with the different compounds are indicated in the legend. The dark colors represent experiments in the presence of molecular oxygen (i.e., without bubbling with nitrogen gas) and the light colors represent experiments in the absence of molecular oxygen (i.e., after bubbling with nitrogen gas).

Section S13: Triplet excitons photogenerated in crystalline TES-pentacene thin films

Triplet excitons were photogenerated in crystalline TES-Pn films via singlet fission to measure their near-infrared photoinduced absorption spectrum. In the present section, we first show that singlet fission is the primary excited-state decay pathway in the crystalline TES-Pn films, and subsequently proceed to measure the near-infrared photoinduced absorption spectrum of triplet excitons in the crystalline TES-Pn films.

Figure S13.1 shows that the molecular-level packing of solvent-vapor annealed (crystalline) TES-Pn films is nearly equivalent to that of solvent-vapor annealed (crystalline) TIPS-Pn films, less some additional mutual displacement along the molecular long axis.[20] Given that in crystalline TIPS-Pn it is well accepted that the primary decay pathway is singlet fission (see e.g. ref. 2 and refs. therein), it is not surprising that singlet fission is the primary decay pathway in the crystalline TES-Pn films. In fact, Pensack *et al.* have reported a number of pentacene derivatives which adopt similar molecular-level packing and that decay primarily via singlet fission.[1,17]

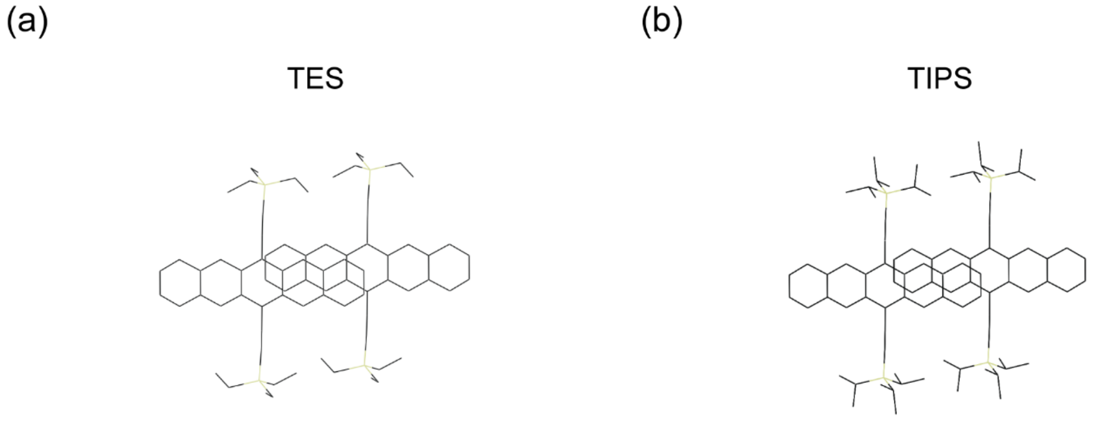

Figure S13.1. Birds-eye view of the molecular packing arrangement extracted from the crystal structures of (a) TES- and (b) TIPS-pentacene. For simplicity, only the molecular pair exhibiting the most orbital overlap is shown.

In contrast to the amorphous TES-Pn nanoparticles that exhibit substantial triplet pair losses at long time, the crystalline TES-Pn films exhibit quantitative (i.e., lossless) triplet pair formation and separation. We showed previously that crystalline films of TIPS-Pn exhibit quantitative triplet pair yields at exceedingly low incident pump fluence.[2] In order to avoid artifacts associated with bimolecular annihilation in the crystalline TES-Pn films so as to be able to highlight differences in the intrinsic singlet fission dynamics in the amorphous TES-Pn nanoparticles and crystalline films, we performed measurements on the crystalline TES-Pn films at exceedingly low incident fluence. **Figure S13.2** shows the transient visible absorption of crystalline TES-Pn films, selected transient spectra, and selected transient kinetics associated with

ground-state bleach and triplet photoinduced absorption features. The results clearly evidence quantitative triplet pair formation and separation in the crystalline TES-Pn films. This is to be contrasted with the significant losses observed on the equivalent timescale in the amorphous TES-Pn nanoparticle suspensions (**Figs. 3f and 4a**).

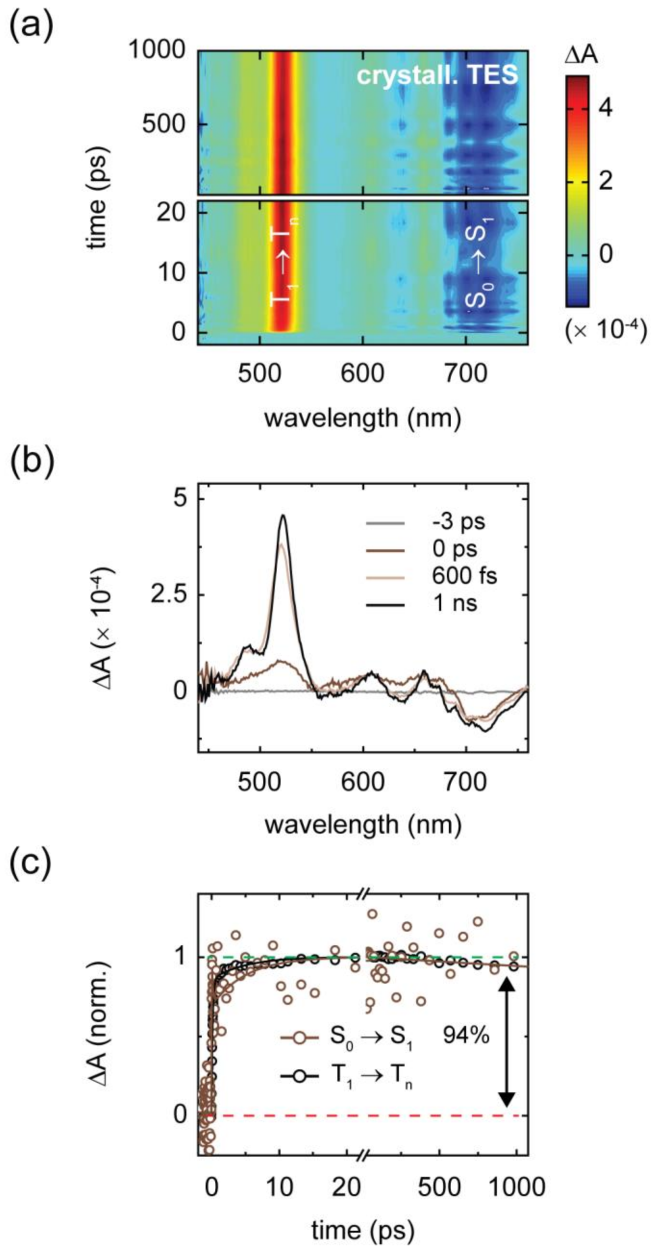

Figure S13.2. (a) Surface plot of transient visible absorption of a crystalline TES-Pn film. The experiment was performed with an incident pump wavelength and fluence of 700 nm and 10 μJ/cm^2, respectively. The scale bar is indicated beside the plot. (b) Selected transient spectra for the same crystalline TES-Pn film. Spectra time delays are indicated in the legend. (c) Selected transient kinetics for the same crystalline TES-Pn film. The kinetics of the triplet photoinduced absorption and ground-state bleach features were taken as the average over the spectral range 510

to 539 nm and 698 to 703 nm, respectively. These data have been normalized over the time range from 20 to 80 ps. A fit from a global analysis overlays these transient absorption data (see ref. 17). It is found that triplet pairs form with a time constant of ca. 200 fs, triplet pair separation occurs with a time constant of ca. 5 ps, and the triplet pair lifetime is >>5 ns (i.e., the duration of the measurement).

It should be noted that there is a slight drop of signal in the crystalline TES-Pn films on the nanosecond timescale, likely resulting from a small residual amount of bimolecular triplet-triplet annihilation. Bimolecular annihilation is particularly problematic in crystalline material that exhibits especially rapid triplet transfer (see e.g. above and ref. 2).

Having demonstrated that singlet fission is the primary decay pathway in the crystalline TES-Pn films, and that both triplet pair formation and separation are quantitative, we are now in a position to measure the transient near-infrared absorption spectrum of triplet excitons photogenerated in the crystalline TES-Pn films. **Figure S13.3** shows the transient near-infrared absorption of a crystalline TES-Pn film, along with the transient spectrum obtained at a time delay of ca. 1 ns.

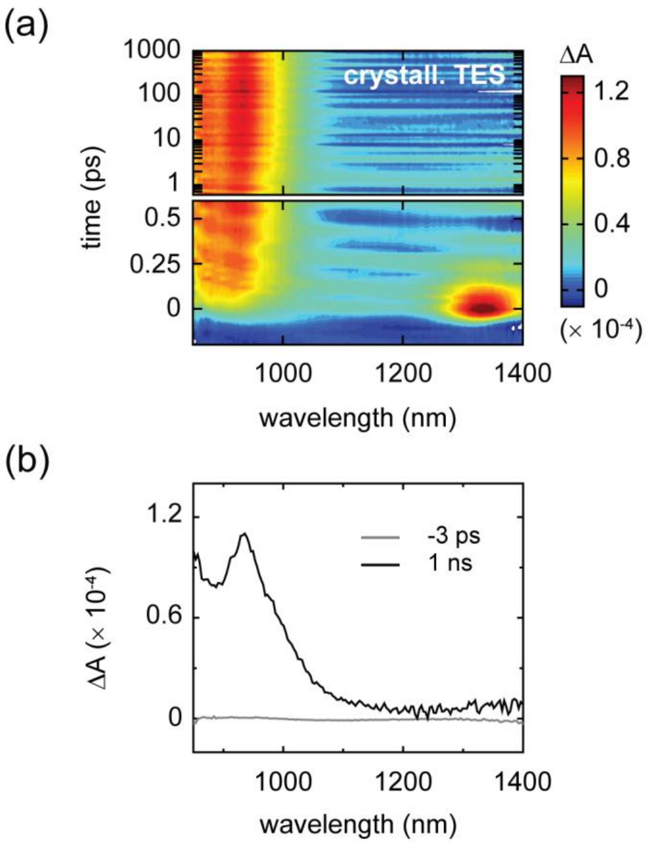

Figure S13.3. (a) Surface plot of transient near-infrared absorption spectra of crystalline TES-Pn

films. The experiment was performed with an incident pump wavelength and fluence of ca. 700 nm and 20 μJ/cm^2, respectively. (b) Selected transient spectra for the same crystalline TES-Pn film. Spectra time delays are indicated in the legend.

We previously showed that in crystalline material of pentacene derivatives the spectrum measured at a timescale of ca. 1 ns is representative of both separated triplet pairs and independent triplet excitons.[17] The transient near-infrared spectrum in **Figure S13.3b** is therefore taken to be representative of both separated triplet pairs and independent triplet excitons and is used in the main text to facilitate spectral assignments.

<u>Section S14:</u> Transient absorption spectrum of intermediate population in TIPS-pentacene nanoparticles can be modeled with corresponding TES- and TSBS-pentacene data

We modeled the transient near-infrared absorption of the intermediate population in the TIPS-Pn nanoparticles using the corresponding transient near-infrared absorption in the TES- and TSBS-Pn nanoparticles as basis spectra (**Figure S14a**). These measurements were performed at identical pump wavelength and fluence, namely, 643 nm and 75 μJ/cm^2. The transient absorption spectra for the TES- and TSBS-Pn nanoparticles were scaled relative to that of the TIPS-Pn nanoparticles taking into account differences in sample optical densities at the pump wavelength, which were 0.32, 0.28, and 0.28 for the TIPS-, TES-, and TSBS-Pn nanoparticles, respectively. The pump fluence was chosen because it falls within a linear scaling regime where singlet-singlet annihilation does not affect the efficiency of the conversion of parent singlet excitons into the nascent triplet pair intermediate, which for the purpose of this estimation we assume is quantitative.

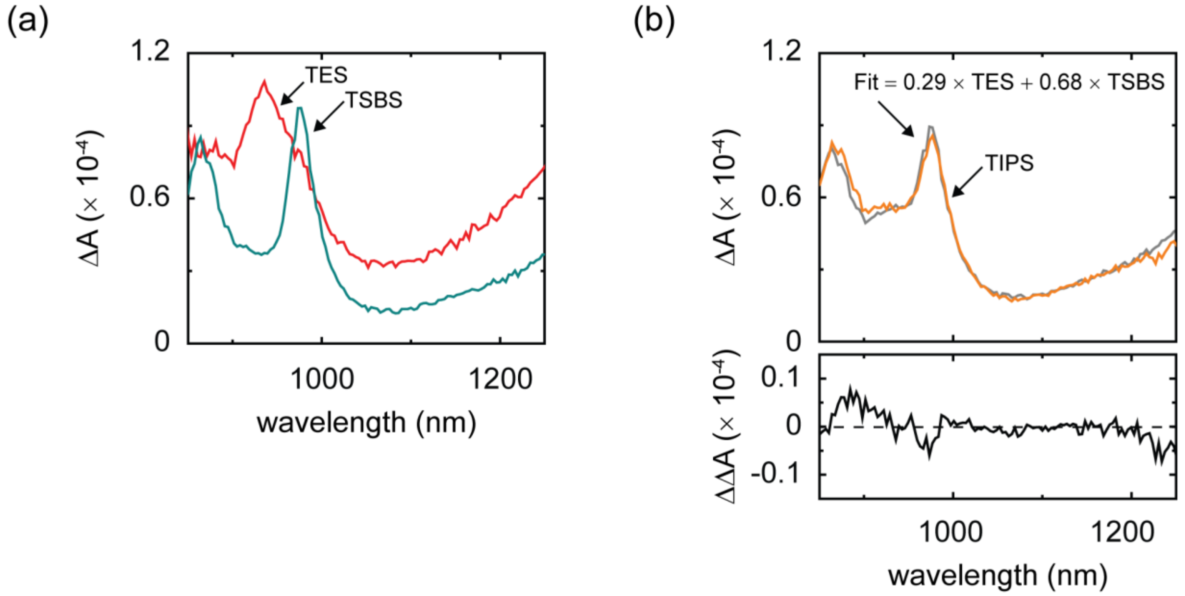

Figure S14. (a) Transient absorption spectra of TES- and TSBS-pentacene nanoparticles time-averaged over the range 3-5 and 20-40 ps, respectively. These measurements were performed at identical pump wavelength and fluence of 643 nm and 75 μJ/cm^2, respectively. (b) Transient absorption spectrum of TIPS-pentacene nanoparticles time-averaged over the range 10-20 ps, and fit using TES- and TSBS-pentacene nanoparticle transient spectra in panel a as basis spectra. The data were fit within the spectral window from 850-1250 nm, which exhibited the highest signal-to-noise ratio in these relatively low fluence (and low signal) measurements. The residual of the fit is indicated below this plot.

Figure S14b shows that the transient near-infrared absorption of the intermediate population in the TIPS-pentacene nanoparticles can be well described in this manner. The fit indicates that intermediate population in the TIPS-pentacene nanoparticles is “mixed”, and consists of 30% and 70% of the intermediate population observed in the TES- and TSBS-pentacene nanoparticles, respectively.

<u>Section S15:</u> Modeling the extinction spectrum of the TIPS-pentacene nanoparticles returns similar population fraction as modeling the transient absorption spectra

The extinction spectrum of the TIPS-Pn nanoparticles was modeled in a similar manner as above, i.e., as a linear combination using the extinction spectra of the TES- and TSBS-Pn nanoparticles as basis spectra (**Figure S15a**). The extinction spectrum of the TIPS-Pn nanoparticles is well modeled by the extinction spectra of the TES- and TSBS-Pn nanoparticles, and the results indicate that the fraction of primary excitations (or parent singlet excitons) in the TIPS-Pn nanoparticles is well described by 50% that of the TES-Pn nanoparticles and 50% that of the TSBS-Pn nanoparticles (**Figure S15b**).

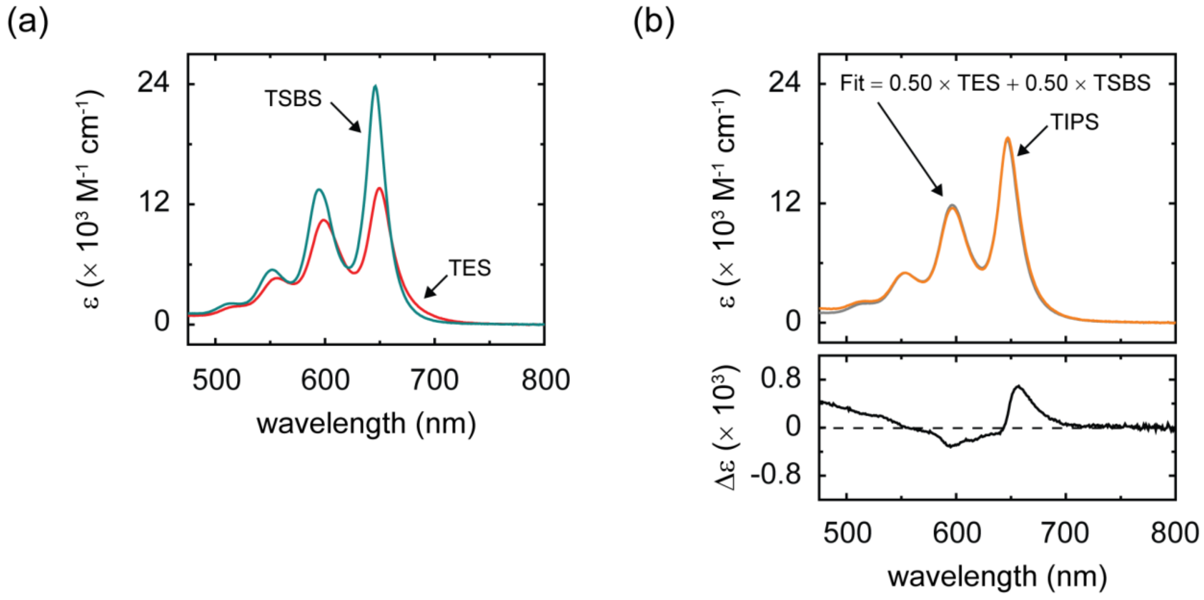

Figure S15. (a) Extinction spectra of TES- and TSBS-pentacene nanoparticles. (b) Extinction spectrum of TIPS-pentacene nanoparticles, and fit using TES- and TSBS-pentacene nanoparticle extinction spectra in panel a as basis spectra. The data were fit within the spectral window from 475-800 nm, which encompasses the lowest-energy singlet transition for these materials. The residual of the fit is indicated below this plot.

We consider the slight discrepancy in the population fraction determined for parent singlet excitons (i.e., in the present supporting section, where we derived a 50/50 modeling ratio) and the intermediate population (i.e., in **Section S14**, where we derived a 30/70 modeling ratio) as likely resulting from: (i) inaccuracies in the measurement of the absolute magnitude transient absorption spectra in **Section S14**, (ii) inaccuracies in the measurement of the extinction spectra in the present section, and/or (iii) energy migration dynamics that are unaccounted for in the present work.

<u>Section S16:</u> A linear equation analysis returns the pure spectra of the parent singlet excitons associated with the two distinct dimer pair sites

In this section, we derive the pure spectra of the parent singlet excitons associated with the two distinct dimer pair sites in the amorphous pentacene derivative nanoparticles.

We first assume that the absolute population fractions for the different dimer pair sites can be determined from the triplet pair losses (**Tbl. 2**). Thus, the lossy and lossless dimer pair sites are determined as reported in **Table S16.1**.

Table S16.1. Absolute Population Fractions for Dimer Pair Sites in Amorphous Pentacene Derivative Nanoparticles

Compound	Φ_{lossy} (%)	$\Phi_{lossless}$ (%)
TES-Pn	81	19
TIPS-Pn	64	36
TSBS-Pn	17	83

Taking these values as representative of the absolute amount of lossy and lossless dimer pair sites in the different amorphous pentacene derivative nanoparticles, we are now in a position to derive the pure spectra associated with the different dimer pair sites. In order to accomplish this, we performed a linear equation analysis using the TES- and TSBS-Pn extinction spectra (**Fig. 2a** and **Figure S15a**). The results of the analysis are displayed in **Figure S16**.

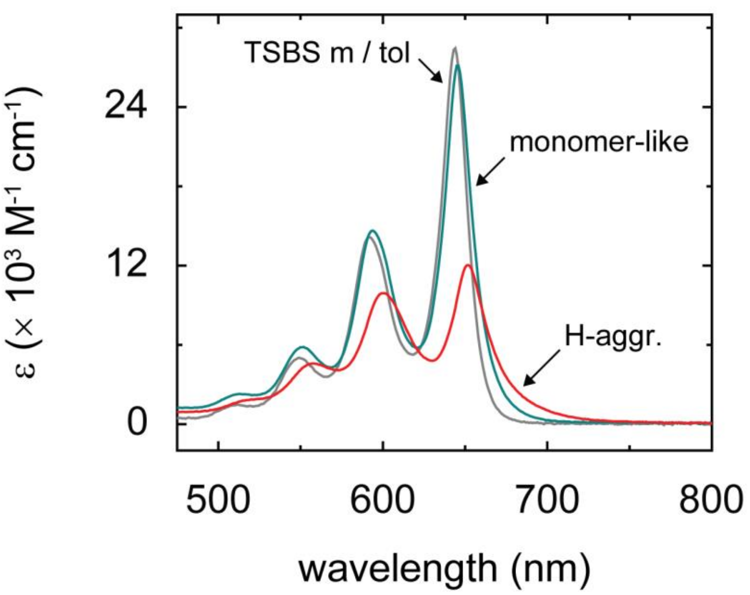

Figure S16. Extinction spectrum of a dilute solution of TSBS-pentacene in toluene along with the "pure" extinction spectra associated with dimer pair sites in the amorphous pentacene derivative nanoparticles comprising monomer-like and weakly-coupled H-aggregated chromophores.

Figure S16 shows the pure spectrum of the parent singlet excitons associated with lossless dimer pair sites which looks remarkably similar to that of the isolated chromophore in dilute toluene solutions. The pure spectrum of the parent singlet excitons associated with lossy dimer pair sites, in contrast, does not resemble that of the isolated chromophore in dilute solution but more strongly resembles the absorption spectrum of the amorphous TES-Pn nanoparticles (see e.g. **Fig. 2a** and **Figure S15.2a**). This latter result is perhaps unsurprising, as the amorphous TES-Pn nanoparticles comprise a large fraction of the lossy dimer pair sites.

With the pure spectra of the parent singlet excitons associated with the lossly and lossless dimer pair sites on hand, it is now possible to learn more about the lossly and lossless dimer pair sites. As we demonstrated in **Section S4**, the relative ratio of the origin band and first vibronic peak can be used to evaluate differences in the average effective excitonic coupling in the different pentacene derivative nanoparticle samples. Having decomposed these spectra into the underlying linear combination spectra, we can gain direct physical insight on the different dimer pair sites from the pure spectra. We find that the relative ratio of the origin band and first vibronic peak is larger than that of the amorphous pentacene derivative nanoparticles in the case of the spectrum of the parent singlet excitons associated with the lossless dimer pair sites and that the ratio is smaller than that of the amorphous TES-Pn nanoparticles in the case of the spectrum of the parent singlet excitons associated with the lossy dimer pair sites. Thus, we can assign the lossless dimer pair sites as originating from weakly-coupled monomer-like chromophores, and the lossy dimer pair sites as comprising H-aggregated chromophores.

Table S16.2. Ratio of the 0-0 and 0-1 Vibronic Bands and Shift of the Lowest-Energy Singlet Transition [a]

**Phase**	$\boldsymbol{A_{0\text{-}0}/A_{0\text{-}1}}$	**Ratio (%)**	$\boldsymbol{E_{\text{singlet}}}$ **(cm^{-1})**	$\boldsymbol{\Delta E_{\text{singlet}}}$ **(cm^{-1})**
m / toluene	2.0	N/A	15,530	N/A
Amorph m	1.9	110	15,500	30
Weak cpl H	1.2	170	15,330	200

[a] The toluene solution monomer data reported in this table were taken as the average of the results for the three different trialkyl-substituted pentacene derivatives.

The trends reported in **Table S16.2** obviously mirror those already discussed in the main text and in **Section S4**. Namely, with more compact side chains, a larger fraction of chromophores exhibiting stronger electronic coupling in an H-aggregate-type and more dense packing configuration are populated. In contrast, as side chain bulkiness increases, a larger fraction of chromophores exhibiting monomer-like properties are populated.

Section S17: Orbital overlap has a profound effect on the triplet near-infrared induced absorption

Empirical evidence suggests that the triplet near-infrared induced absorption of unsubstituted pentacene is sensitive to orbital overlap effects.[21,22] We independently verified that the triplet near-infrared induced absorption of pentacene derivatives is additionally influenced by orbital overlap effects. The chemical compounds that we studied, along with the solid-state order of the associated nanoparticles, are shown in **Figure S17a**.

In the nanoparticles studied in this section, the materials exhibit varying extent of orbital overlap (**Figure S17b**). Nanoparticles of TSBS-Pn are amorphous (**Section S3**), and are assumed to exhibit the least amount of orbital overlap. The nanoparticles of TIBS- and TIPS-Pn reported in this section are both crystalline, and are assumed to exhibit more extensive orbital overlap relative to the amorphous TSBS-PN nanoparticles, although their molecular-level packing compared with one another is different.[1,23] In TIBS-Pn, adjacent pentacene cores are displaced to the extent that they are non-interacting. In this case, two neighboring molecules interact through triple bonds that overlap adjacent pentacene cores. TIPS-Pn, in contrast, exhibits a packing with adjacent pentacene cores directly situated above one another, giving rise to more extensive orbital overlap.

(a)

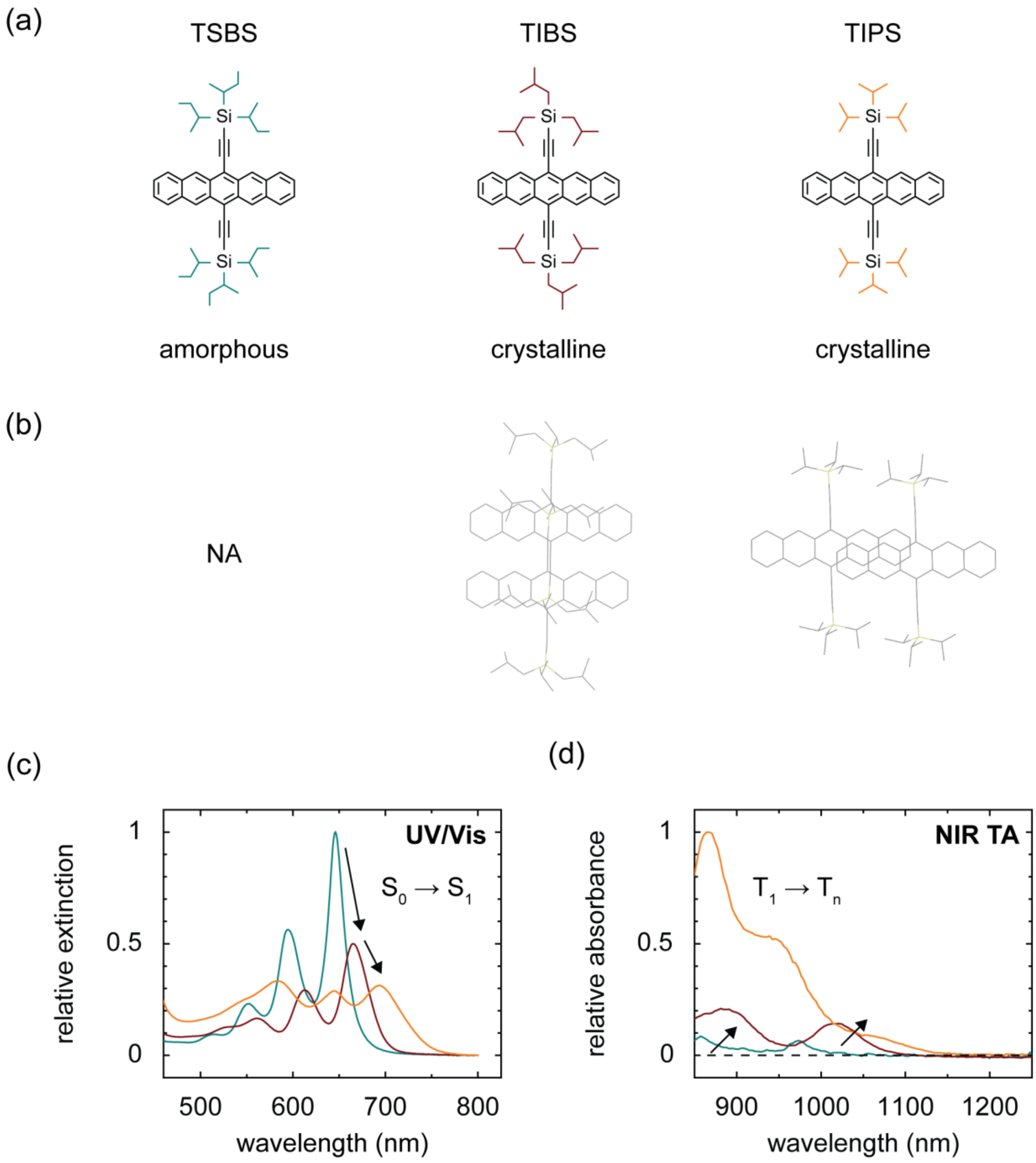

Figure S17. (a) Chemical structures of pentacene derivatives and solid-state order of materials used to study the influence of orbital overlap on ground- and excited-state absorption spectra. (b) Birds-eye view of the molecular packing arrangement extracted from the crystal structures of TIBS- and TIPS-pentacene. For simplicity, only the molecular pair exhibiting the most orbital overlap in crystalline TIPS-pentacene is shown. Molecular pairs in amorphous TSBS-pentacene are not shown, but are assumed to exhibit the least amount of orbital overlap. (c) Relative extinction spectra of the pentacene derivative nanoparticles. The absorption spectrum of the amorphous TSBS-pentacene nanoparticles was normalized to the origin band of its lowest-energy

singlet transition, and the absorption spectra of the crystalline TIBS-pentacene and TIPS-pentacene nanoparticles were scaled according to the relative extinction of the origin band of their lowest-energy singlet transition with respect to that of the amorphous TSBS-pentacene nanoparticles. (d) Transient near-infrared absorption spectra of the nanoparticles obtained at time delays associated with separated triplet pairs (and representative of independent triplet excitons; see, e.g., ref. 17). The transient spectrum of the amorphous TSBS-pentacene nanoparticles was obtained at a time delay of 3 ns whereas the transient spectra of the crystalline TIBS- and TIPS-pentacene nanoparticles were obtained at a time delay of 30 ps. The measurements were performed at pump wavelengths of 645, 665, and 695 nm and fluences of ca. 120, 75, and 40 μJ/cm² for the amorphous TSBS-pentacene, crystalline TIBS-pentacene, and crystalline TIPS-pentacene nanoparticles, respectively. The path length of the spectrophotometer cell was the same for each sample. The amplitude of the transient spectrum of the crystalline TIPS-pentacene nanoparticles was normalized, and the amplitude of the transient spectra of the amorphous TSBS-pentacene and crystalline TIBS-pentacene nanoparticles were scaled relative to that of the crystalline TIPS-pentacene nanoparticles taking into account differences in sample optical densities at the respective pump wavelengths. A scaling factor of 1.33 was applied to the transient spectrum of the amorphous TSBS-pentacene nanoparticles to account for losses accumulated on this timescale.

Figure S17c shows that the extinction spectra of these materials drastically changes with solid-state order, molecular-level packing, and orbital overlap. As shown above, the TSBS-Pn nanoparticles are amorphous and consist of weakly-coupled chromophores **(Section S3)**. For crystalline material, i.e., the TIBS- and TIPS-Pn nanoparticles, the lowest-energy singlet transition becomes redshifted, broadened, and exhibits reduced extinction at its peak maximum. These spectra also exhibit increasingly more complex vibronic structure. The differences are most apparent in the crystalline TIPS-Pn nanoparticles. Given the extremely different molecular-level packing of these crystalline materials (**Figure S17b**), we conclude that increased orbital overlap accounts for differences in their extinction spectra.

Increased orbital overlap also clearly influences the near-infrared absorption spectrum of separated triplet pairs (**Figure 17d**). While the absorption spectrum of separated triplet pairs (that are representative of independent triplet excitons; see, e.g., ref. 17) present in the amorphous TSBS-Pn nanoparticles is very similar to that of isolated-chromophore triplets (**Section S6**), the absorption spectra of separated triplet pairs in crystalline TIBS- and TIPS-Pn become more redshifted, broadened, intense, and exhibit more complex vibronic structure. Overall, the near-infrared absorption spectra of separated triplet pairs are more redshifted, broadened, intense and exhibit more complex vibronic structure in the series amorphous TSBS-Pn < crystalline TIBS-Pn < crystalline TIPS-Pn, consistent with the expectation that orbital overlap strongly influences this triplet excited-state absorption.

Section S18: Detailed justification of six-component kinetic scheme

We previously showed that a five-component kinetic scheme is that which is simplest and best describes the transient absorption of the amorphous TIPS-Pn nanoparticle suspensions.[2] In the present work, the application of this model is extended to amorphous nanoparticle suspensions of two additional pentacene derivatives, TES- and TSBS-Pn. Further, it is demonstrated that an additional component is necessary to accurately model the data, and that the additional component can be attributed to intermolecular structural relaxation immediately following the formation of short-lived nascent triplet pairs.

Figure S18.1 displays the six-component kinetic scheme (also displayed in **Fig. 6a** in the main text) that is used to model the transient absorption of the amorphous pentacene derivative nanoparticle suspensions.

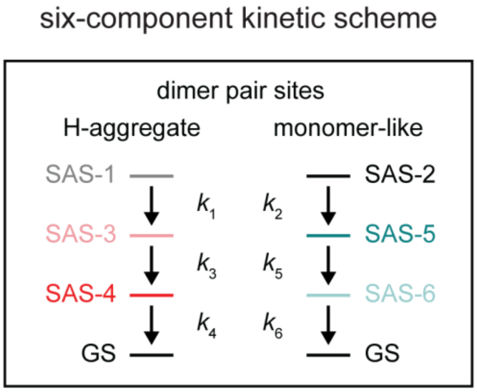

Figure S18.1. Six-component kinetic scheme used for global and target analysis of amorphous pentacene derivative nanoparticles. The six-component kinetic scheme accounts for: (i) two distinct sets of parent singlet exciton and nascent triplet pair populations, i.e., SAS-1/SAS-4 and SAS-2/SAS-5, (ii) short-lived nascent triplet pairs with excess of nuclear kinetic energy, i.e., SAS-3, and (iii) long-lived, spatially separated triplet pairs, i.e., SAS-6.

Figure S18.2 displays the data, fits, and residuals for the amorphous TES-Pn nanoparticle suspensions when applying either a five- or six-component kinetic scheme.

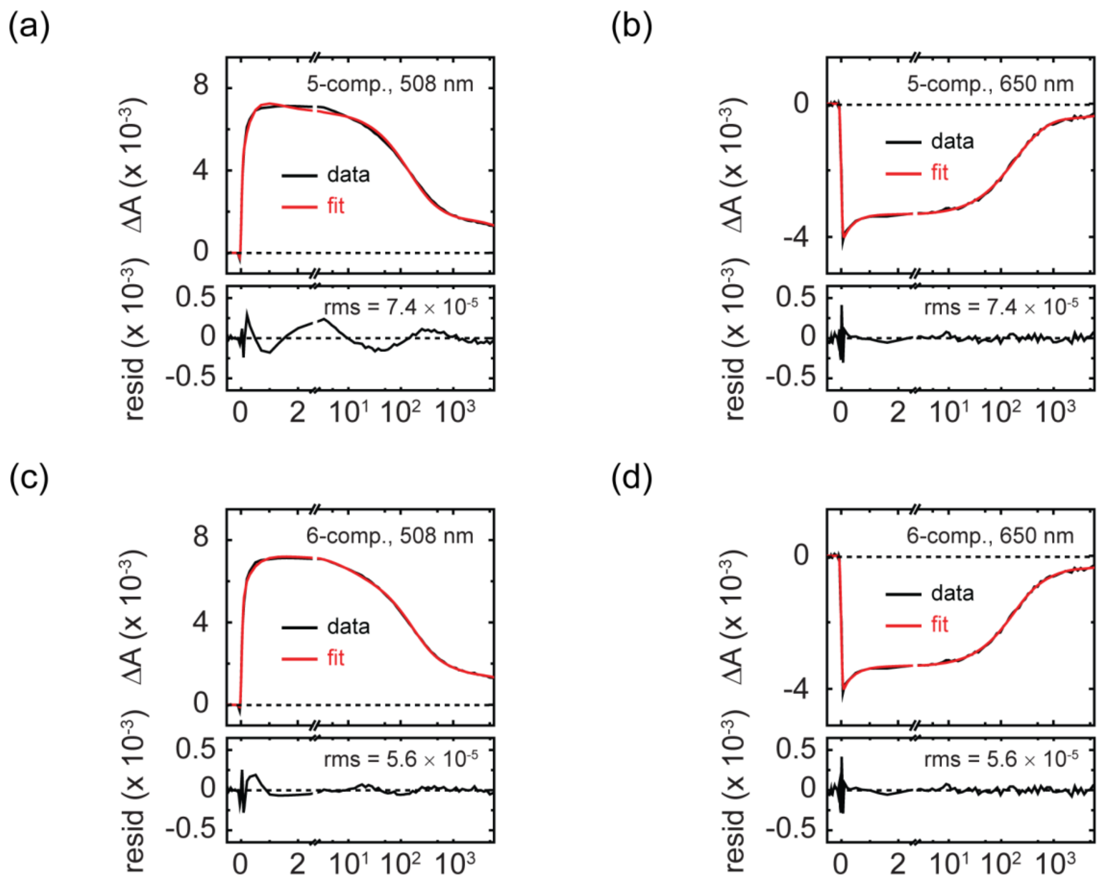

Figure S18.2. Selected transient kinetics traces for the amorphous TES-Pn nanoparticle suspensions in the vicinity of the origin band of the $T_1 \rightarrow T_n$ photoinduced absorption and $S_0 \rightarrow S_1$ ground-state bleach feature at 508 and 650 nm, respectively, along with fits, rms values, and residuals from the global and target analysis. (a,b) Fits, rms values, and residuals associated with the five-component kinetic scheme. (c,d) Fits, rms values, and residuals associated with the six-component kinetic scheme.

The inclusion of an additional component in the model results in an appreciably reduced rms value, and an improved residual especially noticeable in the transient absorption signal in the vicinity of ca. 508 nm. Thus, an additional component is justified on mathematical grounds.

Figures S18.3 and S18.4 show the data, fits, and residuals for the amorphous TIPS- and TSBS-Pn nanoparticle suspensions and show that this conclusion is general to amorphous nanoparticle suspensions of this series of pentacene derivatives.

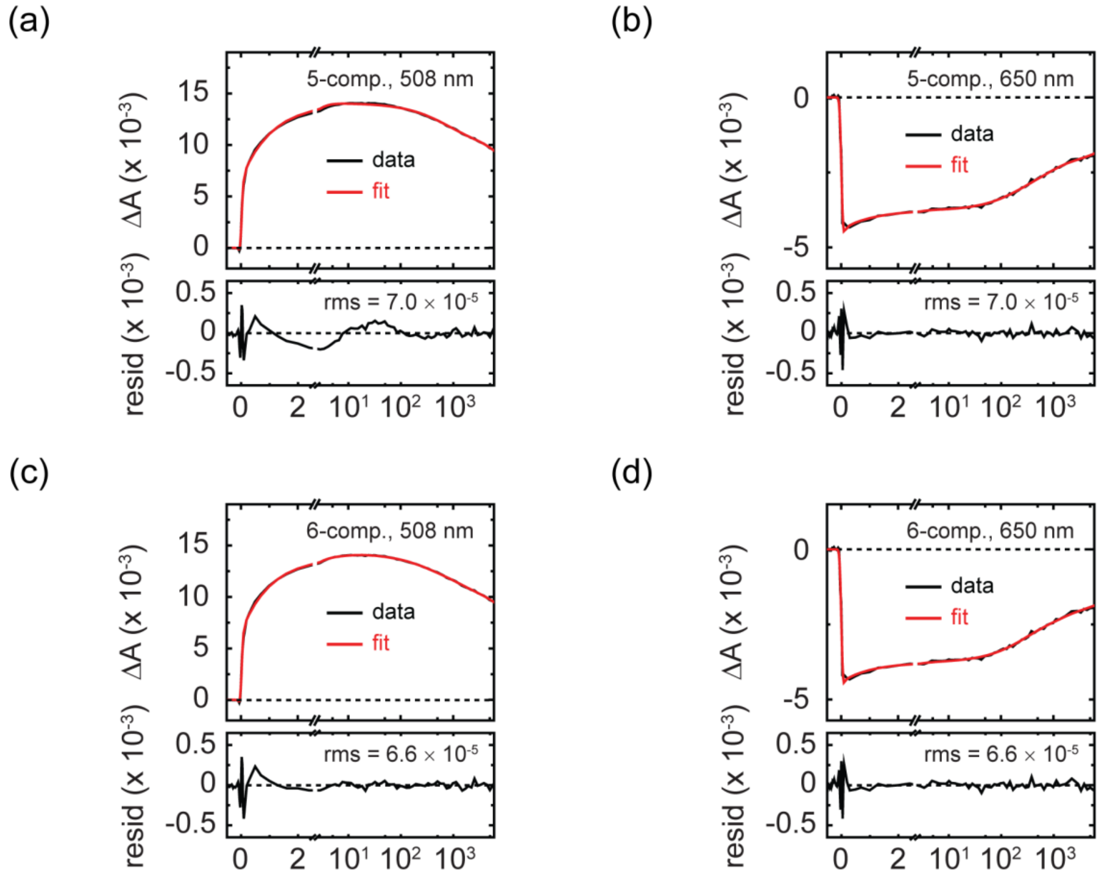

Figure S18.3. Selected transient kinetics traces for the amorphous TIPS-Pn nanoparticle suspensions in the vicinity of the origin band of the $T_1 \rightarrow T_n$ photoinduced absorption and $S_0 \rightarrow S_1$ ground-state bleach feature at 508 and 650 nm, respectively, along with fits, rms values, and residuals from the global and target analysis. (a,b) Fits, rms values, and residuals associated with the five-component kinetic scheme. (c,d) Fits, rms values, and residuals associated with the six-component kinetic scheme.

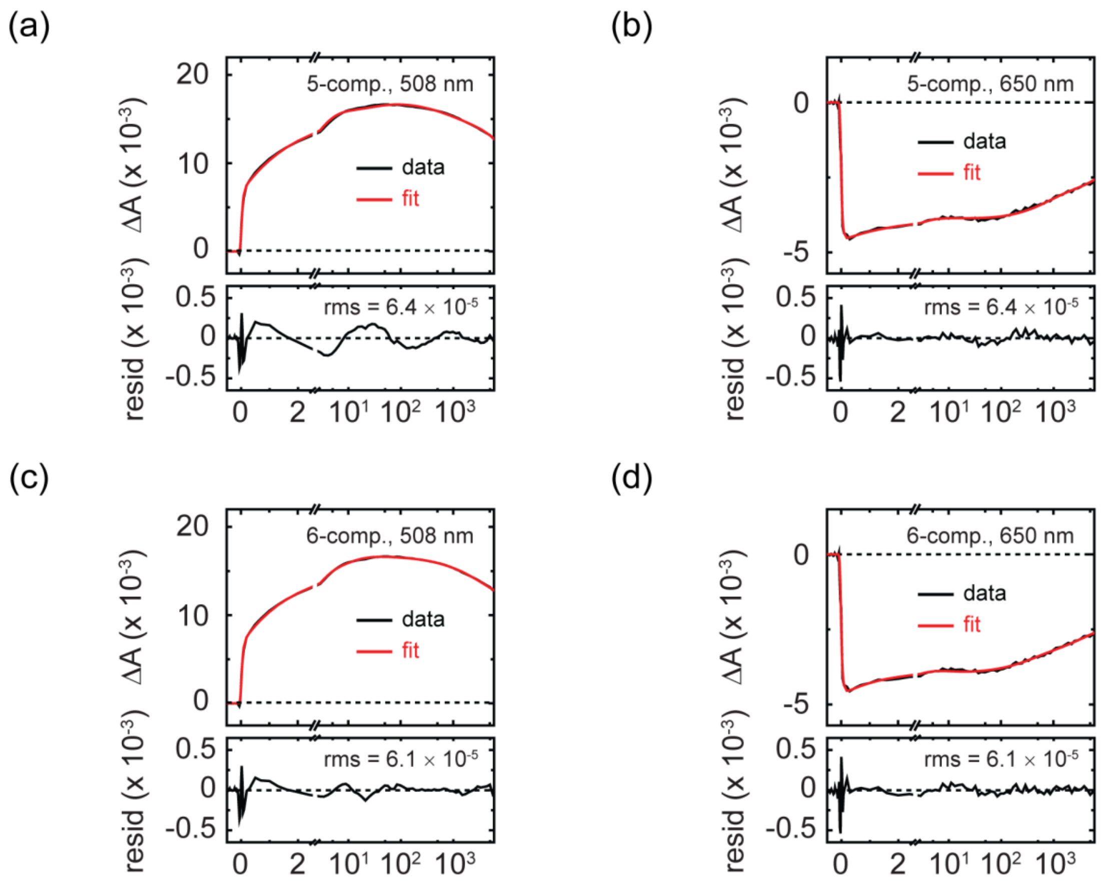

Figure S18.4. Selected transient kinetics traces for the amorphous TSBS-Pn nanoparticle suspensions in the vicinity of the origin band of the $T_1 \rightarrow T_n$ photoinduced absorption and $S_0 \rightarrow S_1$ ground-state bleach feature at 508 and 650 nm, respectively, along with fits, rms values, and residuals from the global and target analysis. (a,b) Fits, rms values, and residuals associated with the five-component kinetic scheme. (c,d) Fits, rms values, and residuals associated with the six-component kinetic scheme.

To explain the physical origin of the additional component, it is necessary to consider the species-associated spectra derived for the two short-lived, nascent triplet pair intermediates, i.e., SAS-3 and SAS-4. **Figure S18.5** displays the SAS-3 and SAS-4 for the amorphous TES-Pn nanoparticles where the comparison is most obvious because the amorphous TES-Pn nanoparticles have the largest population of short-lived nascent triplet pairs. **Figure S18.5** shows that SAS-3 and SAS-4 are not especially dissimilar, differing essentially only in relative peak amplitudes. The comparable but dissimilar spectra can be explained by considering that an appreciable excess of electronic energy is deposited into nuclear kinetic energy in the system over the course of forming short-lived nascent triplet pairs (i.e., short-lived nascent triplet pairs represent a low-energy state). As this internal nuclear kinetic energy is dissipated into the bath, we hypothesize that the mutual displacement of the molecules, which sensitively influences the electronic structure, changes. This is likely a general phenomenon in transient experiments on excitonic systems,[24] and can be considered in some sense similar to the non-monoexponential dynamics reported by Fleming and

co-workers in solutions to explain solvation dynamics;[25,26] that is, the non-monoexponential dynamics observed here can be attributed to microscopic aspects of the system.

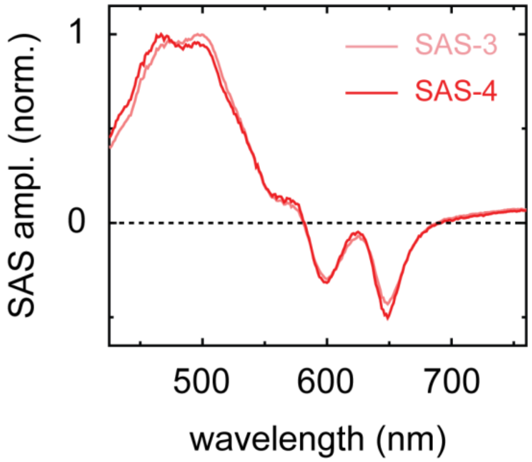

Figure S18.5. Species-associated spectra derived from a six-component global and target analysis of the transient absorption of the amorphous TES-pentacene nanoparticles. Species-associated spectra SAS-3 (pink) and SAS-4 (red) are attributable to “hot” and structurally-relaxed short-lived nascent triplet pairs, respectively.

In summary, the inclusion of an additional component in the kinetic scheme is justified on both mathematical *and* physical grounds.

<u>Section S19:</u> Complete set of species-associated spectra returned from a six-component global target analysis of the transient absorption of all samples

We report here the complete set of species-associated spectra derived from the transient absorption data of the amorphous TES-, TIPS-, and TSBS-Pn nanoparticles via a six-component global and target analysis. We note that due to the complexity of the model, care must be taken in interpreting the entire series of spectra in a quantitative manner. For example, populations with similar spectra and overlapping time constants and small populations with weak signal amplitudes can exhibit severe artifacts due to mixing between components. Thus, while many aspects of these data can be evaluated quantitatively, other aspects should be treated fully qualitatively.

Figure S19.1 displays the species-associated spectra for the two distinct parent singlet exciton populations (SAS-1 and SAS-2). The spectra are qualitatively comparable to the transient spectra of isolated-chromophore singlet excitations (**Figure S5**). Taking SAS-2 as representative, we observe two prominent induced absorption bands in the visible and near-infrared spectral regions at ca. 455 and 1400 nm, respectively, and a distinct stimulated emission band at ca. 730 nm. These spectral features are consistent with those of isolated-chromophore singlet excitations (**Figure S5**). The SAS-1, while qualitatively comparable, exhibit significant artifacts likely as a result of mixing between components due to the similarity of SAS-1 and SAS-2 and the short lifetime of SAS-1. This is especially obvious for the SAS-1 of the amorphous TSBS-Pn nanoparticles, which below ca. 540 nm is essentially a mirror image of SAS-2.

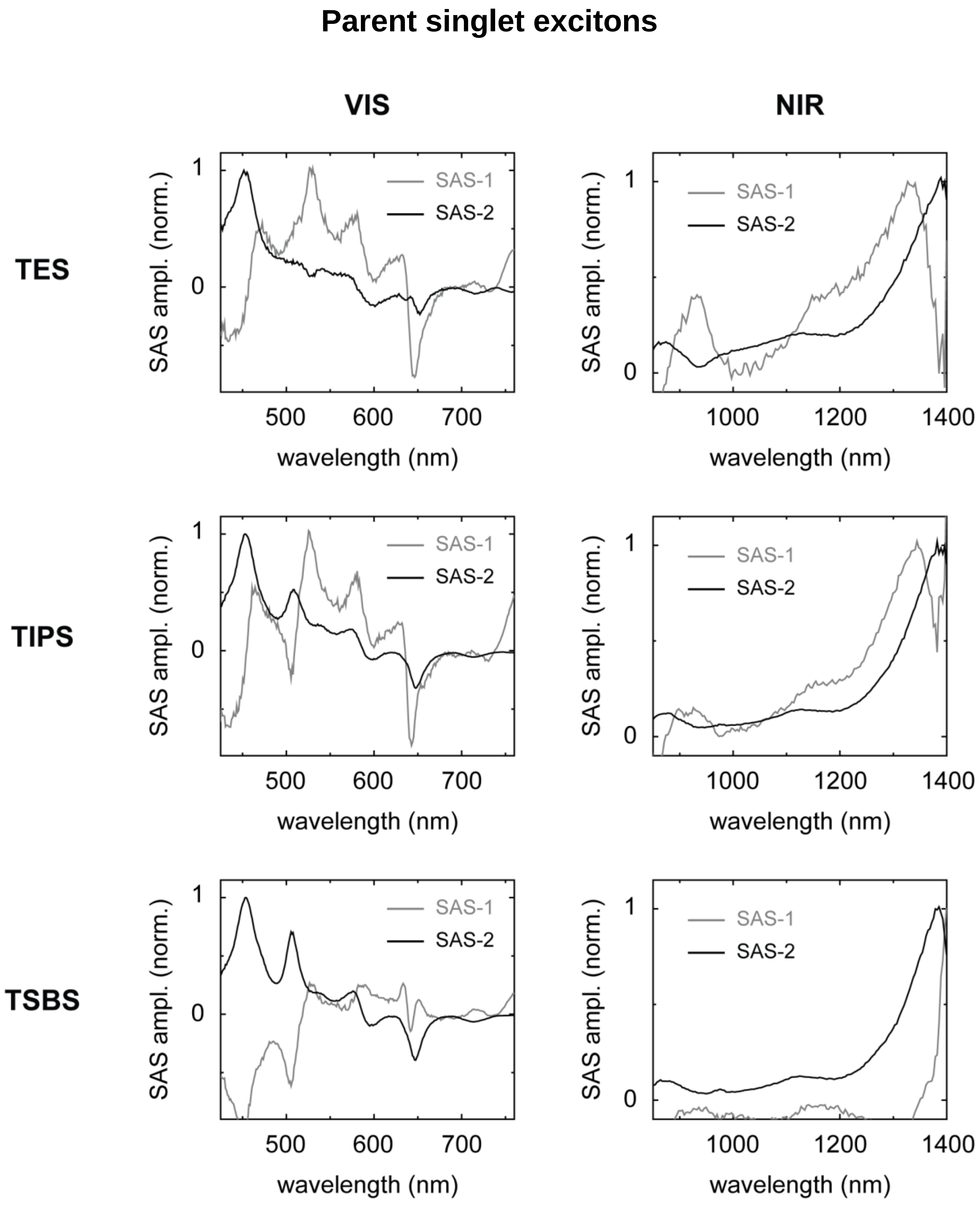

Figure S19.1. Species-associated spectra derived from a six-component global and target analysis of the transient absorption of the amorphous TES-, TIPS-, and TSBS-pentacene nanoparticles. Species-associated spectra SAS-1 (grey) and SAS-2 (black) are attributable to parent singlet excitons populated at dimer pair sites comprising weakly-coupled H-aggregate and monomer-like chromophores, respectively.

Figure S19.2 displays the species-associated spectra for the short-lived triplet pair population (SAS-3 and SAS-4). While the datasets are all qualitatively consistent in their profiles, the data for amorphous TES-Pn nanoparticles are the most representative because of the large population of short-lived triplet pairs in this sample. Taking the SAS-3 and SAS-4 of amorphous TES-Pn nanoparticles as those that are best representative, we observe that SAS-3 and SAS-4 are very similar, with only slight modifications to the photoinduced bands between components. As discussed briefly in **Section S18** above, we attribute these spectral changes electronic structure changes that result from intermolecular geometry relaxation that arises from the dissipation of excess nuclear kinetic energy. We therefore assign the third and fourth components to short-lived nascent triplet pairs that are "hot" and structurally relaxed, respectively. The SAS-3 and SAS-4 of TIPS- and TSBS-Pn nanoparticles exhibit severe artifacts in the vicinity of the triplet photoinduced absorption bands as a consequence of the small signal amplitude (resulting from both lower population and extinction) of this component relative to that of the long-lived triplet pairs.

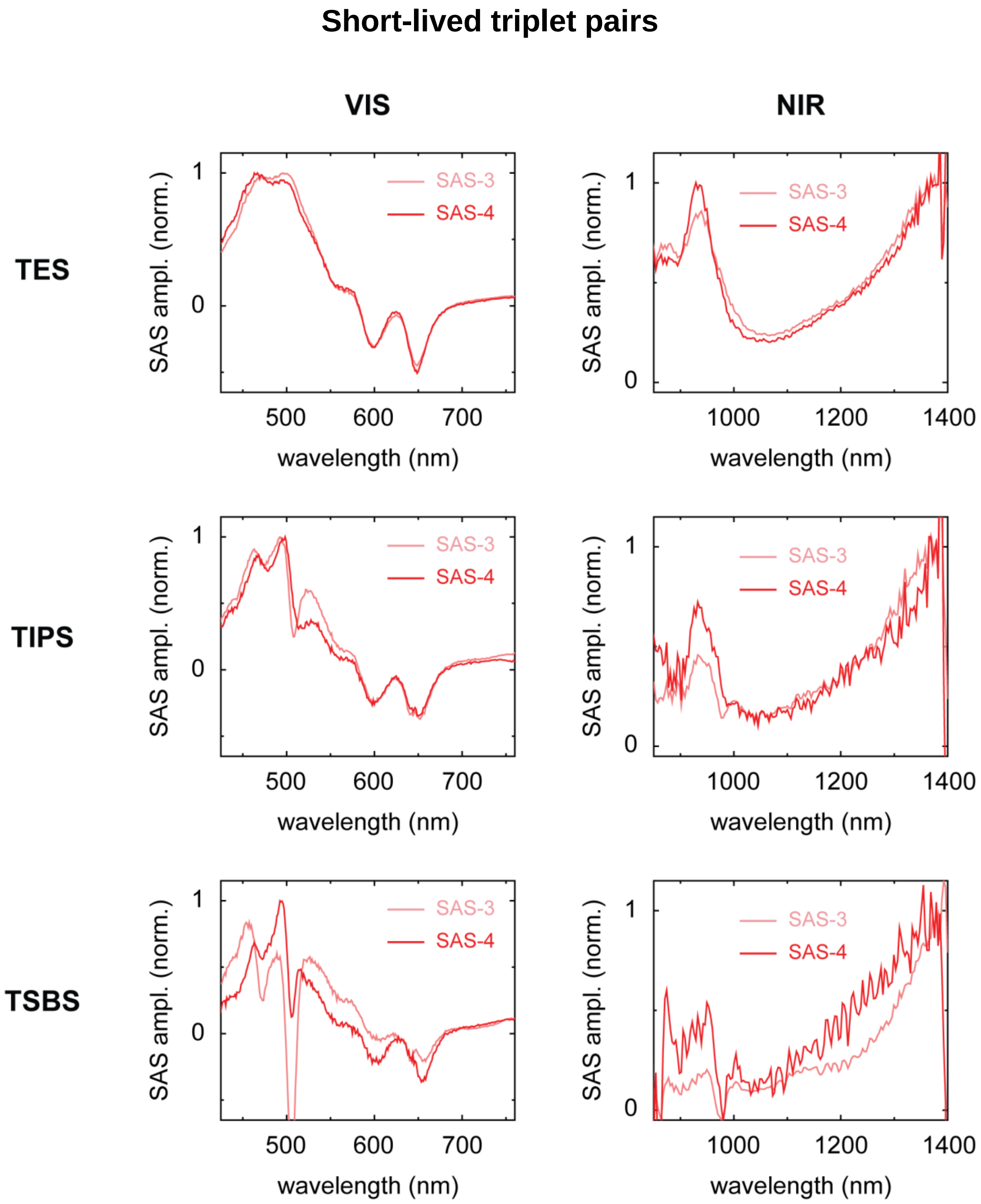

Figure S19.2. Species-associated spectra derived from a six-component global and target analysis of the transient absorption of the amorphous TES-, TIPS-, and TSBS-pentacene nanoparticles. Species-associated spectra SAS-3 (pink) and SAS-4 (red) are attributable to short-lived nascent triplet pairs that are "hot" and structurally relaxed, respectively.

Figure S19.3 displays the species-associated spectra for the long-lived triplet pair population (SAS-5 and SAS-6). Again while the datasets are all qualitatively consistent in their profiles, the data for amorphous TSBS-Pn nanoparticles are the most representative because of the large population of long-lived triplet pairs in this sample. We observe that SAS-5 and SAS-6 are very similar, again with slight modifications to the photoinduced bands between components. Specifically, in the first component (SAS-5) we observe additional photoinduced absorption amplitude in the triplet pair photoinduced absorption spectra in the vicinity of where isolated-chromophore singlet excitations absorb at ca. 1400 and 540 nm and below ca. 500 nm. The second component (SAS-6) exhibits less photoinduced absorption amplitude in these spectral regions, and this is especially apparent when considering the spectral regions at ca. 1400 nm and below ca. 500 nm. These spectral changes were previously assigned to the dissociation of triplet pairs,[17] and the roughly nanosecond time constant associated with the transition between these components (**Tbl. 3**) is consistent with triplet transfer in amorphous material.[2] We therefore assign the fifth and sixth components to long-lived nascent triplet pairs and separated triplet pairs, respectively.

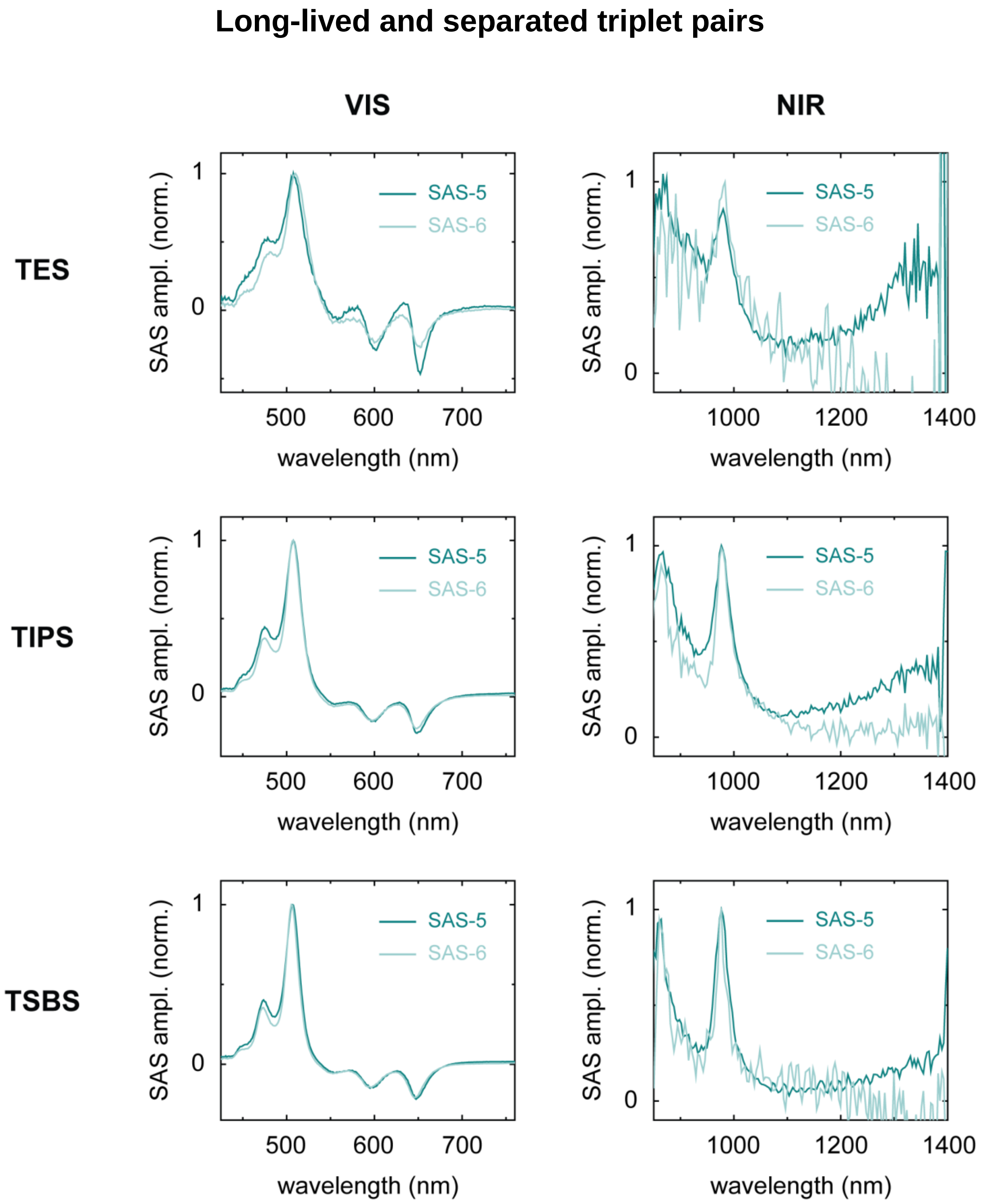

Figure S19.3. Species-associated spectra derived from a six-component global and target analysis of the transient absorption of the amorphous TES-, TIPS-, and TSBS-pentacene nanoparticles. Species-associated spectra SAS-5 (dark cyan) and SAS-6 (light cyan) are attributable to long-lived nascent triplet pairs and separated triplet pairs, respectively.

<u>Section S20:</u> Unimolecular lifetimes of triplet excitations in amorphous TES-, TIPS-, and TSBS-pentacene nanoparticle suspensions

Unimolecular triplet lifetimes were determined from the amorphous TES-, TIPS-, and TSBS-pentacene nanoparticle suspensions by performing fluence-dependent nanosecond transient absorption spectroscopy (**Figure S20**).

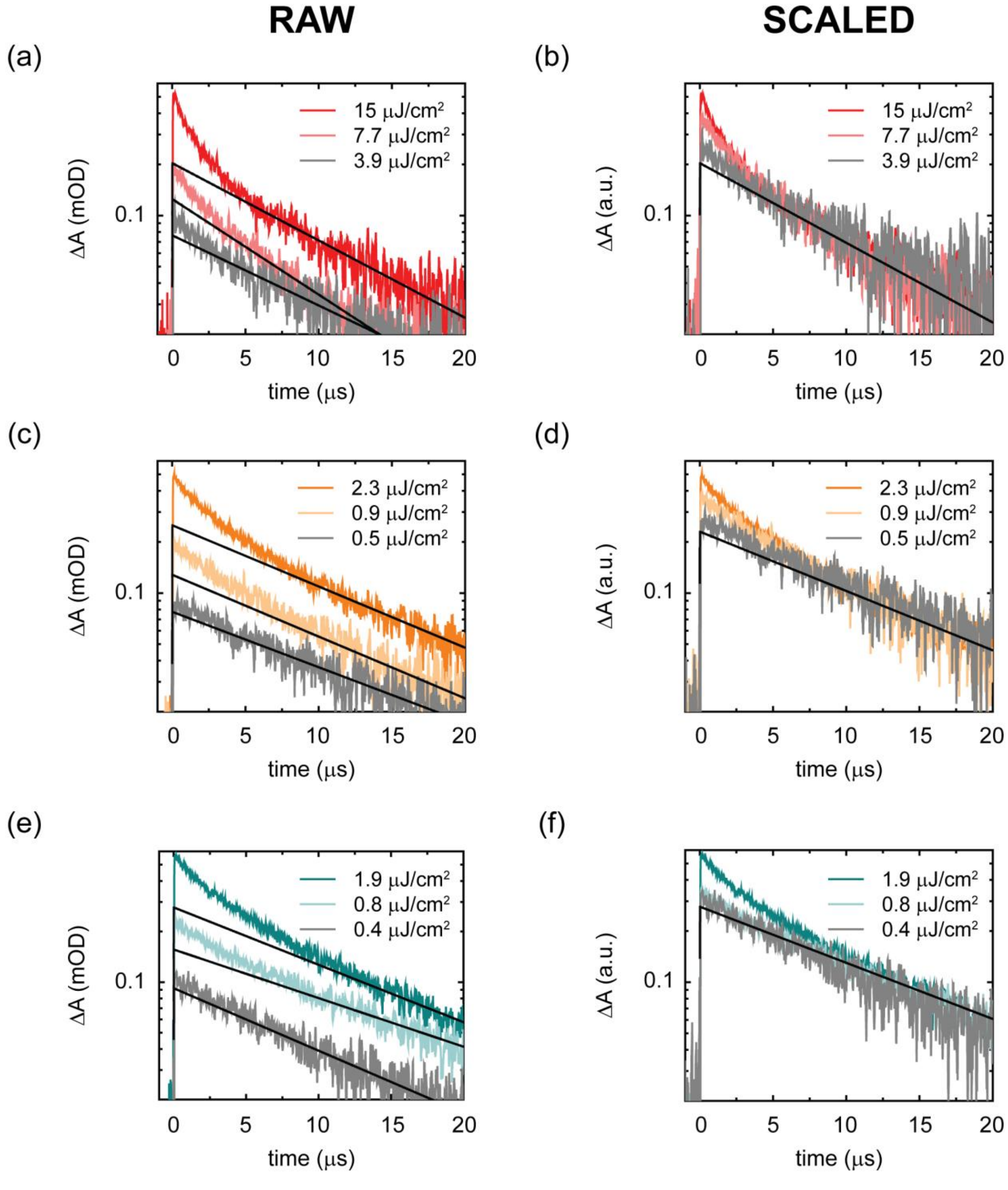

Figure S20. Nanosecond transient absorption kinetics of triplet excitations photogenerated through singlet fission in amorphous pentacene nanoparticle suspensions. The solutions were bubbled with nitrogen gas prior to measurements and were stirred during measurements. The excitation wavelength was 605 nm. The transient absorption kinetics of the triplet excitations were monitored at the origin band of the triplet $T_1 \rightarrow T_n$ photoinduced absorption band peaking at ca. 510 nm (see e.g. refs. 1 and 2). Absorbed energy densities are reported in the legend. The raw data,

including their fits, and the data scaled to the unimolecular component, along with a decay trace according to the average lifetime (see **Table S20**) are plotted for the aqueous nanoparticle suspensions of (a,b) TES-, (c,d) TIPS-, and (e,f) TSBS-pentacene.

The raw data, including fits to the data, for the amorphous TES-, TIPS-, and TSBS-Pn nanoparticle suspensions are displayed in **Figure S20a,c,e**. The average unimolecular lifetime determined according to these fits, along with the standard deviation, is reported in **Table S20**.

Table S20. Unimolecular Triplet Lifetimes for Amorphous Trialkylsilyl-Substituted Pentacene Derivative Nanoparticles [a]

Compound	$\tau_{triplet}$ (μs)
TES-Pn	9 ± 1
TIPS-Pn	12.4 ± 0.8
TSBS-Pn	13 ± 2

[a] Errors values were determined by taking the standard deviation of the fits at three different incident pump fluences.

Figure S20b,d,f displays the fluence-dependent nanosecond transient absorption kinetics scaled so that the unimolecular component in each dataset overlaps. Along with the data are traces plotted that decay according to the average lifetime reported in **Table S20**.

It is interesting to note that the lifetimes measured for triplet excitations in the amorphous pentacene derivative nanoparticles are consistently longer than the isolated-chromophore triplet lifetimes (**Table S10**). It is possible that these slightly increased lifetimes are due to the suppression of nonradiative decay pathways concomitant with the change from the solution to solid state.

<u>Section S21:</u> Complete set of species-associated spectra for the amorphous TIPS-Pn films and time constants derived from the global target analysis

In the present section, we report the complete set of species-associated spectra for the amorphous TIPS-Pn films along with the associated time constants derived from the global target analysis.

Figure S21 displays the complete set of species-associated spectra for the amorphous TIPS-Pn films. Panels a and b display the visible and near-infrared species-associated spectra, respectively, for the short-lived parent singlet exciton and nascent triplet pair populations, and panels c and d display the visible and near-infrared species-associated spectra, respectively, for the long-lived parent singlet exciton and nascent triplet pair populations.

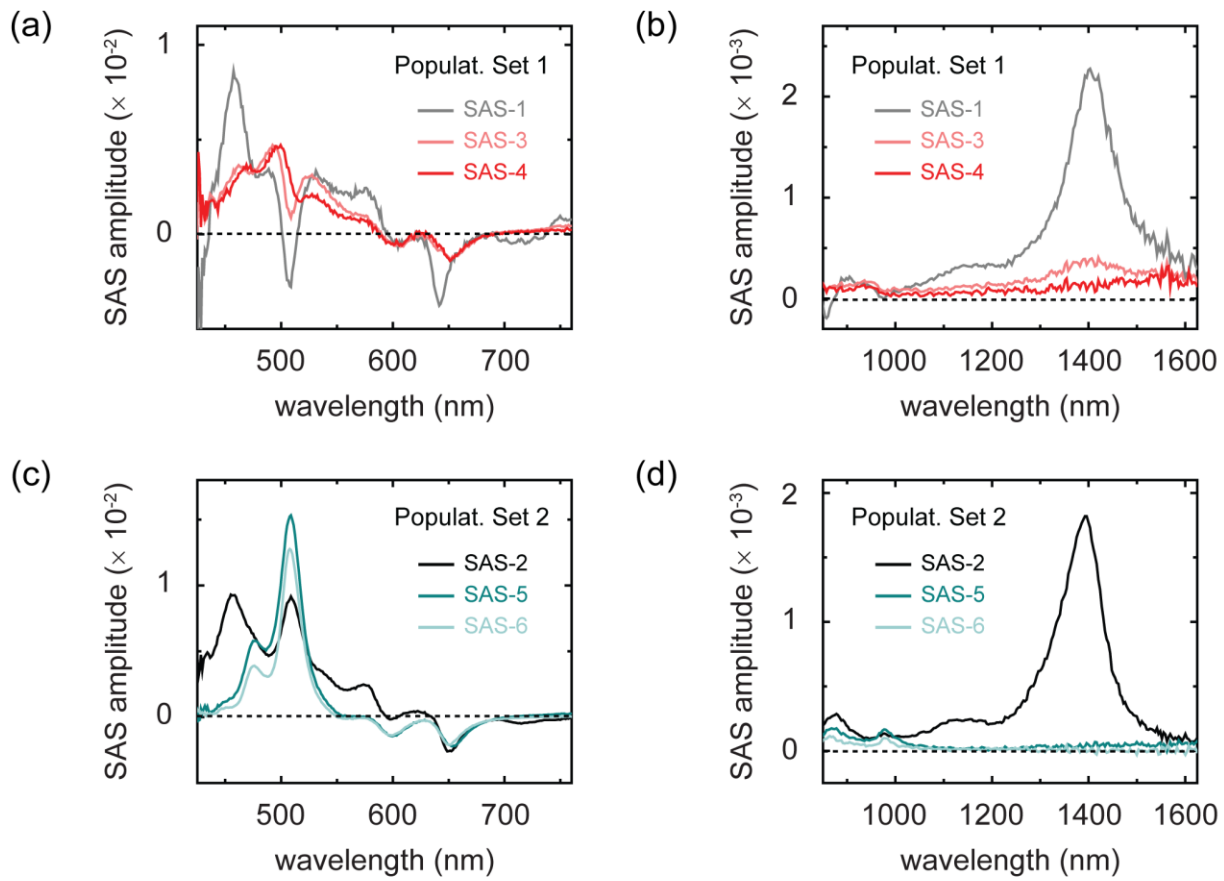

Figure S21. Species-associated spectra derived from a six-component global and target analysis of the transient absorption of the amorphous TIPS-pentacene films. (a) Species-associated spectra SAS-1 (grey), SAS-3 (pink), and SAS-4 (red) attributable to a population set including short-lived parent singlet excitons and "hot" and structurally-relaxed nascent triplet pairs. (b) Species-associated spectra SAS-2 (black), SAS-5 (dark cyan), and SAS-6 (light cyan) attributable to a population set including long-lived parent singlet excitons and nascent triplet pairs and separated triplet pairs.

Many of the artifacts apparent in the species-associated spectra of the amorphous TIPS-Pn nanoparticles are also apparent in that of the amorphous TIPS-Pn films. Namely, component mixing is observed between SAS-1 and SAS-2, which is especially obvious in the visible spectral region, and component mixing is also observed between SAS-1 and SAS-3, which is especially obvious in the near-infrared spectral region. A benefit of the transient absorption of the films is that contributions of nonresonant signals to these species-associated spectra have been minimized.

The time constants derived for the different species-associated spectra are reported in **Table S21**.

Table S21. Six-Component Global Target Analysis of Transient Absorption of Amorphous Trialkylpropylsilyl-Pentacene Films (All Time Constants in Units of ps)

Dimer Pair Sites					
H-Aggregate			Monomer-Like		
τ_{SAS-1}	τ_{SAS-3}	τ_{SAS-4}	τ_{SAS-2}	τ_{SAS-5}	τ_{SAS-6}
0.2	18	180	1.4	860	>> 8000

These values exhibit excellent agreement with those reported in **Tbl. 3** in the main text derived for the amorphous TIPS-Pn nanoparticles. These results indicate that, as we showed previously (see ref. 2), the ultrafast (i.e., sub-10-ns) photophysics of the amorphous TIPS-Pn nanoparticles and films are essentially equivalent.

<u>Section S22:</u> Five Most Basic Two-Molecule Electron Configurations

The five most basic two-molecule electron configurations generally applied to calculate singlet fission rates[21,27] are shown in a schematic form in **Figure S22**.

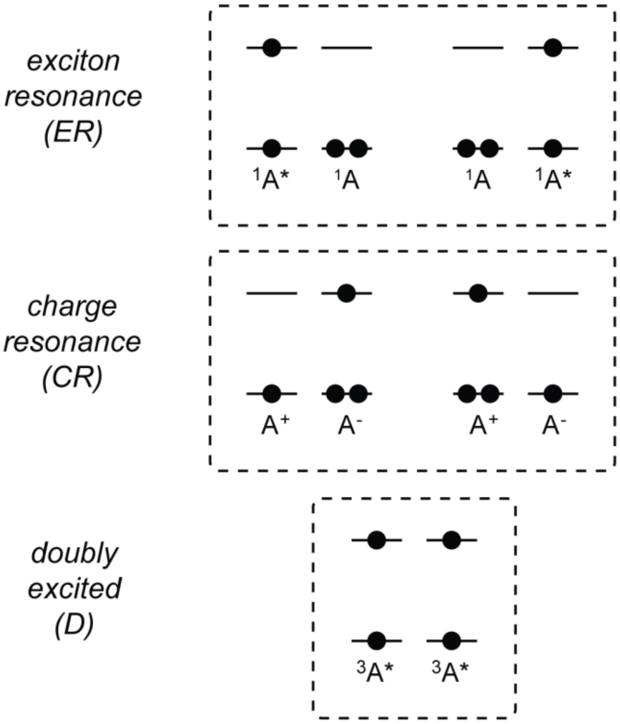

Figure S22. The five most basic two-molecule electron configurations, including two exciton resonance configurations, two charge resonance configurations, and one doubly-excited electron configuration.

As described in detail in the main text, we propose here that these same five two-molecule electron configurations are sufficient to provide a qualitative understanding of the electronic structure of both primary excitations and singlet fission intermediates, i.e., parent singlet excitons, nascent triplet pairs, and spatially separated triplet pairs.

<u>Section S23:</u> How ground-state molecular packing could influence the triplet pair state energy

Short-lived nascent triplet pairs do not resemble isolated-chromophore triplet excitations and do not dissociate, and their distinct dynamics also provide information about the relative triplet pair state energies. Specifically, we showed above that an additional component is required to model the transient absorption data to account for intermolecular structural relaxation following the formation of short-lived nascent triplet pairs (**Fig. 6a**). We highlighted above that this additional component is connected on a microscopic level to the dissipation of a significant amount of excess electronic energy into the surrounding environment. These properties necessarily reflect a highly-stabilized state, as is often observed in classical studies of molecular 'excimer' excitons.[28–31] Thus, the additional component associated with intermolecular structural relaxation following the formation of short-lived nascent triplet pairs that results from the dissipation of a significant amount of excess electronic energy is consistent with an interpretation wherein widely different triplet pair state energies result in a highly-stabilized triplet pair state.

Additional insight into how molecular packing at the different dimer pair sites results in these disparate energy-level orderings within the triplet pair state manifold can be elucidated from the steady-state and transient absorption spectra of the parent singlet excitons and nascent triplet pairs, respectively. We showed, for example, that short-lived nascent triplet pairs are populated at sites comprising H-aggregate dimer pairs (**Fig. 5** and **Section S16**) where the constituent molecules exhibit orbital overlap (**Section S17**). We speculate that the cofacial, contact packing at the sites comprising H-aggregate dimer pairs causes the triplet pair states of lower multiplicity (i.e., the triplet pair states that are overall singlet and triplet that can mix with charge resonance configurations[27]) to become appreciably lower in energy relative to the overall quintet triplet pair states so as to stabilize the overall triplet pair state energy.

Section S24: Nascent triplet pairs do not emit light

In this section, we provide strong evidence indicating that triplet pairs populated in the pentacene derivative nanoparticles are non-emissive. In addition to the data presented in **Fig. 9** of the main text, this interpretation is based on an analysis indicating good agreement between measured fluorescence quantum yields and those estimated for the amorphous pentacene derivative nanoparticles according to a model assuming that triplet pairs do not emit light, and that all light emission is derived from parent singlet excitons.

Table S24.1 shows the fluorescence quantum yields estimated for the amorphous pentacene derivative nanoparticles assuming that triplet pairs do not emit light, and that all light emission is derived from parent singlet excitons. The time constants for the decay of the parent singlet excitons were taken from **Section S9**. The rate constants were determined from the time constants. The fluorescence quantum yields were estimated via a simple kinetic analysis,[32] by evaluating how effective the decay of parent singlet excitons in the amorphous pentacene derivative nanoparticles, which was taken to be equivalent to that of the measured fluorescence decay of isolated chromophores in toluene reported in **Section S8**, is able to compete with decay via singlet fission.

Table S24.1. Fluorescence Quantum Yields Estimated Assuming All Light Emission from Parent Singlet Excitons (and No Light Emission from Triplet Pairs) [a,b]

Compound	τ_{S1S0} (ps)	k_{S1S0} ($\times 10^{12}$ s^{-1})	Φ_{est} ($\times 10^{-4}$)
TES-Pn	0.42 ± 0.03	2.4 ± 0.2	0.35 ± 0.03
TIPS-Pn	1.23 ± 0.06	0.82 ± 0.04	1.02 ± 0.05
TSBS-Pn	2.8 ± 0.5	0.36 ± 0.07	2.3 ± 0.4

[a] The time constant for decay of parent singlet excitons was obtained by averaging over three independent measurements (and sample preparations). The limits represent an analysis of a single standard deviation of the time constants obtained from the fits. See e.g. **Section S9**.
[b] Error limits for the rate constant and estimated fluorescence quantum yield were propagated from the error associated with the time constant derived in **Section S9**.

We additionally measured the FQY for the amorphous pentacene derivative nanoparticles, according to methods described in **Section S1**. In brief, relative fluorescence quantum yield measurements were performed. Oxazine-1 was used as a relative standard. The samples had an optical density of ≤ 0.1 at the peak maximum of the vibronic origin band of the lowest-energy singlet transition and an optical density of ≤ 0.05 at the excitation wavelength of 596 nm. The FQY values measured in this manner are reported in **Table S24.2**.

Table S24.2. Fluorescence Quantum Yields Measured for Amorphous Pentacene Derivative Nanoparticles [a]

Compound	Φ_{meas} ($\times 10^{-4}$)
TES-Pn	0.3 ± 0.1
TIPS-Pn	0.5 ± 0.2
TSBS-Pn	0.9 ± 0.5

[a] The fluorescence quantum yield for the amorphous pentacene derivative nanoparticle suspensions was obtained by averaging over three independent measurements (and sample preparations). The limits represent an analysis of a single standard deviation of the measured fluorescence quantum yield values.

The measured and estimated FQY for amorphous pentacene derivative nanoparticles are in excellent agreement. The measure and estimated FQY for TES-Pn nanoparticles match identically, and those measured and estimated for amorphous TIPS- and TSBS-Pn differ in magnitude only by a factor of two. For the latter, in fact, the measured values are slightly lower than those estimated. It is possible that limitations in the relative method used to measure the fluorescence quantum yields from the amorphous nanoparticle suspensions resulted in this error. It is also possible that the choice of Oxazine-1 as the relative standard may have given rise to this error. For example, although the dye was chosen because its emission spectrum overlaps that of the pentacene derivatives, the two sets of molecules have appreciably different vibronic structure. Irrespective of these details, we consider the good agreement between the measured fluorescence quantum yields and those estimated assuming all light is emitting by parent singlet excitons to indicate that triplet pairs do not emit light in pentacene derivative solids. These results are consistent with strongly exoergic singlet fission, and no equilibrium between the parent singlet exciton and the nascent triplet pair.

Section S25: **No bimolecular annihilation in amorphous TIPS- or TSBS-Pn nanoparticles**

We previously demonstrated that the transient absorption of amorphous TIPS-Pn nanoparticle suspensions is largely independent of incident pump fluence on the timescale of the transient absorption measurements in the present work (i.e., sub-10-ns). See e.g. Section S18 of ref. 2.

Figure S25 shows that the transient absorption of the amorphous TSBS-Pn nanoparticle suspensions also is essentially independent of fluence over a range of ca. 15 – 240 μJ/cm^2, again clearly demonstrating that bimolecular triplet-triplet annihilation is not responsible for the decay of the transient absorption signal of triplet excitations.

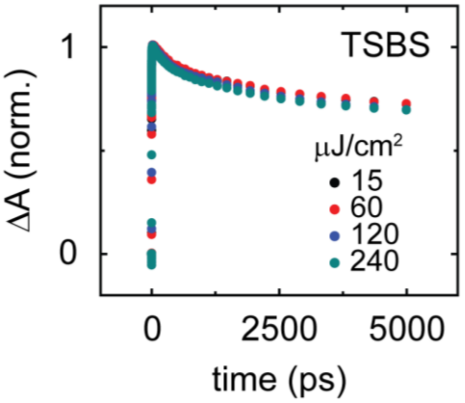

Figure S25. Decay kinetics for the triplet photoinduced absorption feature in amorphous TSBS-pentacene nanoparticles measured over fluences ranging from 15 – 240 μJ/cm^2.

It is interesting to compare the relative insensitivity of the transient absorption of triplet excitations of the amorphous TES-, TIPS-, and TSBS-Pn nanoparticles to that of crystalline material. For example, we previously showed that in contrast to the amorphous pentacene derivative nanoparticles, crystalline pentacene derivative solids measured over a similar range of fluences exhibit a striking fluence dependence consistent with substantial bimolecular triplet-triplet annihilation.[1,2,4] This comparison should not appear too surprising, given that the time constant for triplet transfer in crystalline pentacene derivatives, including TIPS-Pn, has been measured to be of the order of ca. 1 ps,[1,2,17,33] whereas in the amorphous phase this time constant has been measured to be three orders-of-magnitude slower, i.e., of the order of ca. 1 ns (ref. 2 and this work). Thus, it is unsurprising that bimolecular triplet-triplet annihilation is not observed in the amorphous pentacene derivative nanoparticles on the several nanosecond timescale in the transient absorption measurements reported here. Indeed, some signatures of bimolecular triplet-triplet annihilation on the 10s to 100s of nanoseconds timescale are evident in the transient absorption measurements reported out to a timescale of several 10s of microseconds in **Section S20**.

Section S26: Why geminate triplet-triplet annihilation does not explain losses

First, the mechanism of triplet-triplet annihilation in the pentacene derivative nanoparticles is discussed. Interested readers are also referred to Poletayev *et al.* who have already discussed this topic in some detail.[34] In brief, there are two possible ways triplets can annihilate in the pentacene derivative solids studied in this work:

(S26.1) $T_1 + T_1 \rightarrow S_1 + S_0 \rightarrow S_0 + S_0$
(triplet fusion to lowest singlet state, followed by internal conversion)

(S26.2) $T_1 + T_1 \rightarrow T_n + S_0 \rightarrow T_1 + S_0$
(triplet annihilation to high-lying triplet state, followed by internal conversion)

Equation S26.1 is ruled out, however, because it is highly energetically unfavorable (i.e., S_1 ~ 1.91-1.92 eV compared with 2 x T_1 ~ 1.54 eV, using the T_1 value of Zirzlmeier *et al.*[35]). The argument that S_1 does not reform is consistent with the complete lack of fluorescence from these amorphous nanoparticles (ref. 1 and **Section S24**), and solids of pentacene and its derivatives more generally.

This leaves **Equation S26.2** to account for the triplet-triplet annihilation, that Poletayev et al. also concluded as the most likely annihilation pathway.[34] These authors noted a high-lying triplet state, calculated by taking the sum of the energy of the lowest-energy triplet and the transition energy associated with the triplet induced absorption observed in the near-infrared in crystalline pentacene (e.g., see **Sections S6 and S17** above); for the present compounds, i.e., trialkylsilylethynyl-substituted pentacenes, this high-lying triplet state has a value of 0.77 + 1.27 = 2.04 eV. This value is even higher in energy than that of the parent singlet exciton, and again is highly energetically unfavorable, as also noted by Poletayev *et al*. We posit here that triplet states with intermediate energy, which are not optically coupled with the lowest-energy triplet state, or which are observable through lower-energy excited-state transitions that have not yet been reported, likely mediate the triplet-triplet annihilation.

We can now relate this triplet-triplet annihilation to expected transient absorption signals. According to **Equation S26.2**, and considering the complete geminate annihilation of pairs of triplet excitations, the transient absorption signal should asymptotically reach a value of 50%.

We next consider the timescale associated with geminate triplet-triplet annihilation. Given that geminate triplet-triplet annihilation assumes the presence of two independent triplet excitations, we can take the elementary step associated with geminate triplet-triplet annihilation to be that associated with triplet transfer. That is, we can take the timescale of triplet transfer to be representative of the fastest timescale that geminate triplet-triplet annihilation could take place. For the amorphous TES-, TIPS-, and TSBS-Pn nanoparticles, we derived time constants of ca. 0.6, 1, and 2 ns, respectively, for triplet pair separation, which we assigned to a triplet transfer process.[1,17] Thus, a single geminate triplet-triplet annihilation event can occur no faster than ca. 0.6, 1, and 2 ns in the amorphous TES-, TIPS-, and TSBS-Pn nanoparticles, respectively.

Taking 0.6 ns as the timescale for triplet transfer in the amorphous TES-Pn nanoparticles, assuming the annihilation occurs according to **Equation S26.2**, and additionally accounting for the relative population fraction of short- and long-lived nascent triplet pairs (**Tbl. 2**), we can predict the extent to which the signal should decay and compare it with the observed decay.[36] **Figure S26** shows that while the predicted decay for geminate triplet-triplet annihilation reaches its asymptotic limit of 59% by ca. 5 ns, the observed signal has decayed substantially more, i.e., to a value of ca. 25% within a timescale of ca. 0.6 ns. We therefore rule out geminate triplet-triplet annihilation as the origin of the decay of the signal.

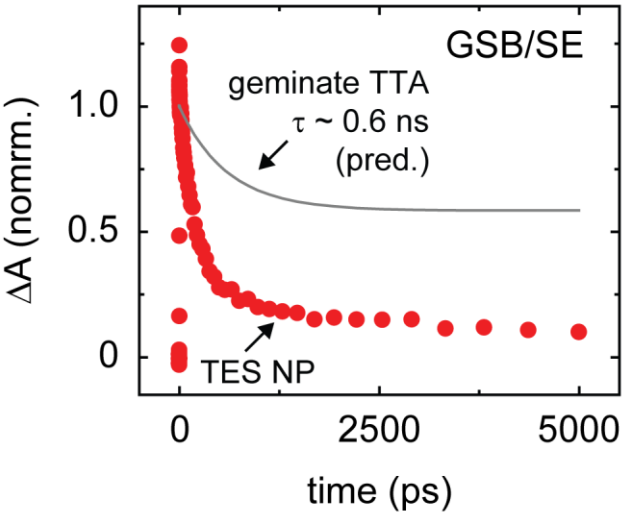

Figure S26. A comparison of the decay of the ground-state bleach / stimulated emission feature of the amorphous TES-pentacene nanoparticles with that predicted for geminate triplet-triplet annihilation to high-lying triplet states with a time constant of ca. 0.6 ns.

<u>Section S27:</u> Amorphous TSBS-Pn nanoparticles transient absorption is also temperature independent

As we showed in **Fig. 10b** in the main text, the TES-Pn nanoparticles do not exhibit an appreciable temperature-dependent transient absorption signal.

Figure S27 shows that the transient absorption signal of the TSBS-Pn nanoparticles is also temperature independent.

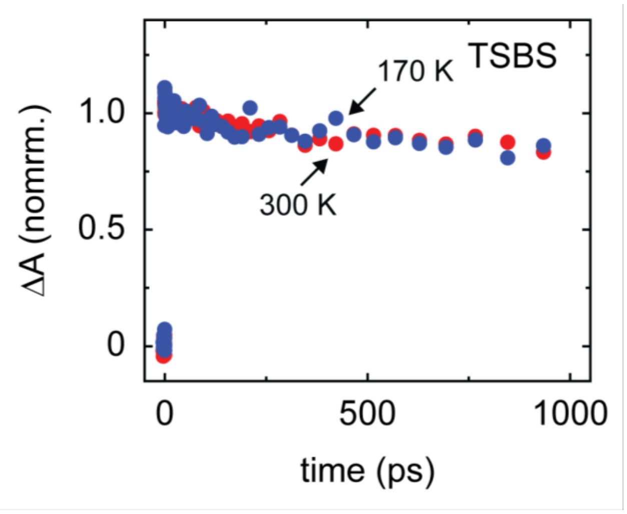

Figure S27. Temperature-dependent transient absorption of the TSBS-pentacene nanoparticles. Specifically, the ground-state bleach kinetics obtained at 300 and 172 K are compared.

<u>Section S28:</u> References

1 R. D. Pensack, A. J. Tilley, S. R. Parkin, T. S. Lee, M. M. Payne, D. Gao, A. A. Jahnke, D. G. Oblinsky, P.-F. Li, J. E. Anthony, D. S. Seferos and G. D. Scholes, *J. Am. Chem. Soc.*, 2015, **137**, 6790–6803.
2 R. D. Pensack, C. Grieco, G. E. Purdum, S. M. Mazza, A. J. Tilley, E. E. Ostroumov, D. S. Seferos, Y.-L. Loo, J. B. Asbury, J. E. Anthony and G. D. Scholes, *Mater. Horiz.*, 2017, **4**, 915–923.
3 With strong excitonic effects being defined as exhibiting substantially different peak positions, peak splitting, and/or different absorption intensities when compared with the isolated chromophore.
4 C. Grieco, G. S. Doucette, R. D. Pensack, M. M. Payne, A. Rimshaw, G. D. Scholes, J. E. Anthony and J. B. Asbury, *J. Am. Chem. Soc.*, 2016, **138**, 16069–16080.
5 J. B. Birks, in *Photophysics of Aromatic Molecules*, Wiley-Interscience, London, 1970, pp. 1–28.
6 J. B. Birks, in *Organic Molecular Photophysics*, ed. J. B. Birks, Wiley-Interscience, London, 1973, vol. I, pp. 1–55.
7 F. C. Spano, *Acc. Chem. Res.*, 2010, **43**, 429–439.
8 M. Kasha, *Radiat. Res.*, 1963, **20**, 55–70.
9 This is to be contrasted with the observation expected for J-type or head-to-tail π-stacking in molecular aggregates, namely, an increased ratio of the amplitude of the 0-0 and 0-1 vibronic bands. See, e.g., ref. 7.
10 J. Clark, C. Silva, R. H. Friend and F. C. Spano, *Phys. Rev. Lett.*, 2007, **98**, 206406.
11 A. Köhler and H. Bässler, *Electronic Processes in Organic Semiconductors: An Introduction*, Wiley-VCH, Weinheim, 2015.
12 P. O. Morawska, Y. Wang, A. Ruseckas, C. Orofino-Peña, A. L. Kanibolotsky, R. Santhanagopal, N. Fröhlich, M. Fritsch, S. Allard, U. Scherf, P. J. Skabara, I. D. W. Samuel and G. A. Turnbull, *J. Phys. Chem. C*, 2015, **119**, 22102–22107.
13 K. J. Thorley, T. W. Finn, K. Jarolimek, J. E. Anthony and C. Risko, *Chem. Mater.*, 2017, **29**, 2502–2512.
14 B. J. Walker, A. J. Musser, D. Beljonne and R. H. Friend, *Nat. Chem.*, 2013, **5**, 1019–1024.
15 S. T. Roberts, R. E. McAnally, J. N. Mastron, D. H. Webber, M. T. Whited, R. L. Brutchey, M. E. Thompson and S. E. Bradforth, *J. Am. Chem. Soc.*, 2012, **134**, 6388–6400.
16 P. I. Dron, J. Michl and J. C. Johnson, *J. Phys. Chem. A*, 2017, **121**, 8596–8603.
17 R. D. Pensack, E. E. Ostroumov, A. J. Tilley, S. Mazza, C. Grieco, K. J. Thorley, J. B. Asbury, D. S. Seferos, J. E. Anthony and G. D. Scholes, *J. Phys. Chem. Lett.*, 2016, **7**, 2370–2375.
18 S. N. Sanders, E. Kumarasamy, A. B. Pun, M. T. Trinh, B. Choi, J. Xia, E. J. Taffet, J. Z. Low, J. R. Miller, X. Roy, X.-Y. Zhu, M. L. Steigerwald, M. Y. Sfeir and L. M. Campos, *J. Am. Chem. Soc.*, 2015, **137**, 8965–8972.
19 G. B. Piland and C. J. Bardeen, *J. Phys. Chem. Lett.*, 2015, **6**, 1841–1846.
20 For solvent-vapor annealed (crystalline) TES-pentacene films, see CCDC # 1028702. For solvent-vapor annealed (crystalline) TIPS-pentacene films, see CCDC # 172476. It is interesting to note that while the molecular-level packing is essentially the same for the solvent-vapor annealed (crystalline) films of the two compounds, the long-range order is quite different.
21 M. B. Smith and J. Michl, *Annu. Rev. Phys. Chem.*, 2013, **64**, 361–386.

22 M. W. B. Wilson, A. Rao, J. Clark, R. S. S. Kumar, D. Brida, G. Cerullo and R. H. Friend, *J. Am. Chem. Soc.*, 2011, **133**, 11830–11833.
23 J. E. Anthony, J. S. Brooks, D. L. Eaton and S. R. Parkin, *J. Am. Chem. Soc.*, 2001, **123**, 9482–9483.
24 Pensack *et al.* **2018**, submitted.
25 E. Castner, M. Maroncelli and G. Fleming, *J. Chem. Phys.*, 1987, **86**, 1090–1097.
26 M. Maroncelli and G. Fleming, *J. Chem. Phys.*, 1987, **86**, 6221–6239.
27 M. B. Smith and J. Michl, *Chem. Rev.*, 2010, **110**, 6891–6936.
28 J. M. Lim, P. Kim, M.-C. Yoon, J. Sung, V. Dehm, Z. Chen, F. Würthner and D. Kim, *Chem. Sci.*, 2013, **4**, 388–397.
29 E. A. Margulies, L. E. Shoer, S. W. Eaton and M. R. Wasielewski, *Phys. Chem. Chem. Phys.*, 2014, **16**, 23735–23742.
30 K. E. Brown, W. A. Salamant, L. E. Shoer, R. M. Young and M. R. Wasielewski, *J. Phys. Chem. Lett.*, 2014, **5**, 2588–2593.
31 C. M. Mauck, R. M. Young and M. R. Wasielewski, *J. Phys. Chem. A*, 2017, **121**, 784–792.
32 N. J. Turro, J. C. Scaiano and V. Ramamurthy, *Modern Molecular Photochemistry of Organic Molecules*, University Science Books, Sausalito, CA, 2010.
33 See also **Section S13** where we derive a time constant of ca. 5 ps for triplet pair separation in crystalline thin films of TES-Pn.
34 A. D. Poletayev, J. Clark, M. W. B. Wilson, A. Rao, Y. Makino, S. Hotta and R. H. Friend, *Adv. Mater.*, 2014, **26**, 919–924.
35 J. Zirzlmeier, D. Lehnherr, P. B. Coto, E. T. Chernick, R. Casillas, B. S. Basel, M. Thoss, R. R. Tykwinski and D. M. Guldi, *Proc. Natl. Acad. Sci.*, 2015, **112**, 5325–5330.
36 We note and highlight that this analysis presumes that geminate triplet-triplet annihilation occurs with a 100% probability with each triplet transfer event, and this is not necessarily likely to be the case.
